# Isoquinoline Antimicrobial Agent: Activity against Intracellular Bacteria and Effect on Global Bacterial Proteome

**DOI:** 10.3390/molecules27165085

**Published:** 2022-08-10

**Authors:** Caroline W. Karanja, Nimishetti Naganna, Nader S. Abutaleb, Neetu Dayal, Kenneth I. Onyedibe, Uma Aryal, Mohamed N. Seleem, Herman O. Sintim

**Affiliations:** 1 Department of Chemistry, Purdue University, 560 Oval Drive, West Lafayette, IN 47907, USA; 2 Department of Comparative Pathobiology, Purdue University College of Veterinary Medicine, 625 Harrison Street, West Lafayette, IN 47907, USA; 3 Department of Biomedical Sciences and Pathobiology, Virginia-Maryland College of Veterinary Medicine, Virginia Polytechnic Institute and State University, 1410 Prices Fork Rd, Blacksburg, VA 24061, USA; 4 Purdue Institute of Inflammation, Immunology and Infectious Disease, West Lafayette, IN 47907, USA; 5 Purdue Proteomics Facility, Bindley Bioscience Center, Purdue University, West Lafayette, IN 47907, USA; 6 Institute for Drug Discovery, Purdue University, 720 Clinic Drive, West Lafayette, IN 47907, USA

**Keywords:** antimicrobial resistance, allkynyl isoquinolies, Sonogashira coupling, intracellular clearance

## Abstract

A new class of alkynyl isoquinoline antibacterial compounds, synthesized via Sonogashira coupling, with strong bactericidal activity against a plethora of Gram-positive bacteria including methicillin- and vancomycin-resistant *Staphylococcus aureus (S. aureus)* strains is presented. HSN584 and HSN739, representative compounds in this class, reduce methicillin-resistant *S. aureus* (MRSA) load in macrophages, whilst vancomycin, a drug of choice for MRSA infections, was unable to clear intracellular MRSA. Additionally, both HSN584 and HSN739 exhibited a low propensity to develop resistance. We utilized comparative global proteomics and macromolecule biosynthesis assays to gain insight into the alkynyl isoquinoline mechanism of action. Our preliminary data show that HSN584 perturb *S. aureus* cell wall and nucleic acid biosynthesis. The alkynyl isoquinoline moiety is a new scaffold for the development of potent antibacterial agents against fatal multidrug-resistant Gram-positive bacteria.

## 1. Introduction

Many compounds, which inhibit bacterial growth, have been reported since the discovery of penicillin. Yet, the multitude of reported compounds with antibacterial activities have not provided an insurance against an era in which high mortality due to infections by multi-drug resistant bacteria will be the norm [1,2,3]. Millions of drug-resistant infections are reported annually in the U.S., with fatalities from these infections well over 30,000 [4]. Additionally, the financial burden associated with treatment of bacterial infections runs into USD billions [5]. The overuse of traditional antibiotics, coupled with the slow introduction of new classes of antibacterial agents, have contributed to the rising incidences of multidrug-resistant bacterial infections [6]. In its bid to guide development of new antibacterial agents, the World Health Organization (WHO) announced a priority list of drug-resistant bacteria for which new antibacterial agents are vital for human health [7]. Methicillin-resistant *Staphylococcus aureus* (MRSA), vancomycin-resistant *S. aureus* (VRSA) and vancomycin-resistant *Enterococcus faecium* (VRE *faecium*) were the highest ranked Gram-positive bacteria [7]. Most of the antibacterial agents used today are from the ‘golden age’ of antibiotics, or are analogs thereof, and the indiscriminate use of these agents have led to the emergence of bacterial strains that are resistant to these staple drugs [8,9,10]. The introduction of new classes of antibiotics, such as oxazolidinones and cyclic lipopeptides, have a significantly improved treatment of resistant Gram-positive bacterial infections, such as multidrug-resistant staphylococci, and VRE [11]. However, the emergence of linezolid-resistant staphylococci highlights the need for new antibacterial classes to keep up with resistance evolution [12].

In addition to the emergence of multidrug-resistant strains, the survival of bacteria inside mammalian cells also contributes to poor clinical outcomes. Intracellular bacteria are deemed to be responsible for the clinical failures of antibiotics and infections relapse [13,14,15,16]. Twaites and Gant referred to leukocytes as “trojan horses” for metastatic *S. aureus* infections [13]. Consequently, for the successful treatment of *S. aureus* infections, it is vital that the antibacterial agents are active against both extracellular and intracellular bacteria. Mammalian cells offer a protective niche for bacteria, and the efficacy of many antibiotics are significantly reduced when tested against intracellular bacteria [16,17]. This is mainly due to the poor mammalian cell permeability of many antibiotics [16]. As a result, several strategies have been proposed to improve the intracellular accumulation of antibacterial agents. Lehar and co-authors used a protease-sensitive antibody–antibiotic conjugate to deliver rifampicin into mammalian cells [16]. On the other hand, Xiong et.al used nanogels to facilitate vancomycin uptake by macrophages [18]. While these approaches significantly increased drug cellular uptake, there is a poor correlation between drug accumulation and activity when it comes to intracellular clearance [17]. This antibiotic mammalian cell permeation issue, coupled with the emergence of drug-resistant bacteria [19], calls for new molecular entities that have potent activities against bacteria and are also active against intracellular pathogens.

**Figure 1:**
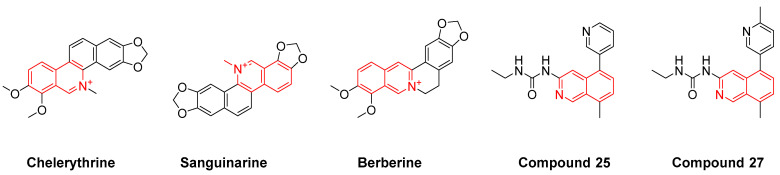
Structures of antimicrobial compounds with an isoquinoline moiety.

Antibiotic drug discovery has followed two paths: target-based or phenotypic screening. Phenotypic screening, however, has the potential of identifying compounds with a unique mode of action and could also deliver drugs that target more than one essential pathway, which appears to be the preferred mode of killing to prevent bacterial resistance development. Our group has been developing novel isoquinoline moiety chemical libraries for drug screening [20,21,22]. Considering that several compounds with an isoquinoline moiety have been reported to possess antimicrobial activity [23,24,25], for example, compounds 25 and 27 (Figure 1) potently kill *S. aureus* with a minimal inhibitory concentration (MIC) of 0.5 µg/mL [25], we decided to screen our proprietary isoquinoline-based library against *S. aureus* to identify antimicrobial agents. In this paper, we report a new class of alkynyl isoquinolines active against a plethora of Gram-positive bacteria. Surprisingly, these compounds exhibit low frequency of resistance and also clear intracellular bacteria.

## 2. Results and Discussion

### 2.1. Screening for Antibacterial Active Alkynyl Isoquinolines

**Figure 2:**
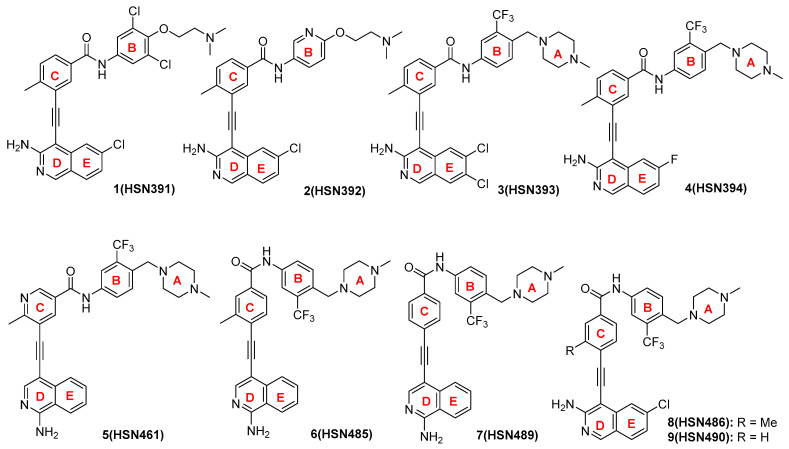
Structures of alkynyl isoquinolines with antibacterial activity.

**Figure 3:**
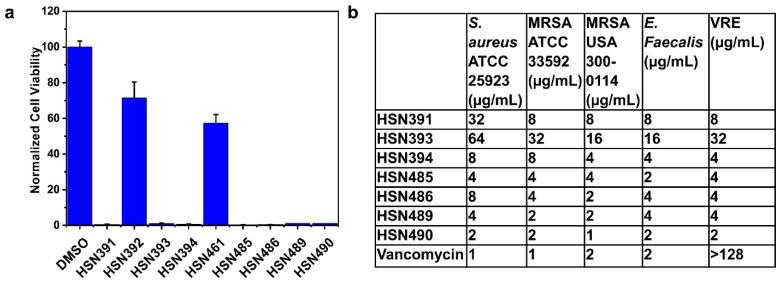
Alkynyl isoquinoline inhibition of bacteria growth. (**a**) *S. aureus* cell viability after treatment with 16 µg/mL alkynyl isoquinolines. (**b**) Minimal inhibitory concentrations for alkynyl isoquinolines compounds against methicillin-sensitive *S. aureus*, methicillin-resistant *S. aureus* (MRSA)*,* vancomycin-sensitive *E. faecalis* and vancomycin-resistant *E. faecalis* (VRE).

In our efforts to identify a new class of antibacterial agent, we screened our alkynyl isoquinoline library for antibacterial active compounds against *S. aureus*. The *S. aureus* cells were incubated at 37 °C overnight with 16 µg/mL of compounds. Cell viability was determined via optical density measurements. We identified several compounds that completely inhibited bacterial growth (Figure 2 and Figure 3A). For a more precise depiction of the compounds’ antibacterial activity, the minimal inhibitory concentration (MIC) was determined for those compounds with over 80% growth inhibition. The significant difference in activity levels between HSN391 and HSN392 indicates the substitution pattern of ring B (see Figure 2 for ring labeling) is vital for activity. The variance of MICs between HSN393 and HSN394 shows that substitution of the isoquinoline ring is also important for activity. On the other hand, by comparing the MICs of HSN485 versus HSN489, and those of HSN486 versus HSN490, we can conclude that the methyl substitution of ring C does not affect activity. However, adding a nitrogen to ring C significantly affects the activity. HSN490 exhibited the best antibacterial activity against *S. aureus,* and *Enterococcus faecalis* (Figure 3B). Hence, for further studies, HSN490 was chosen as the lead compound.

**Figure 4:**
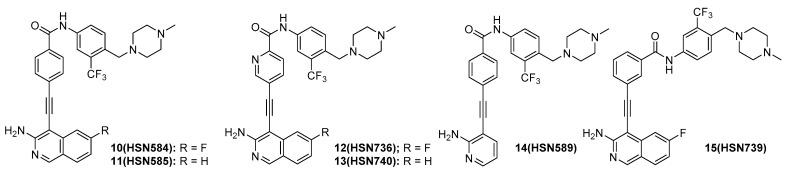
HSN490 analogs used for the structure activity relationship study.

Next, we sought to determine which moieties were important for antibacterial activity. The replacement of the chloro group on the isoquinoline moiety with a fluoro group (HSN584) did not change activity (Figure 4 and Figure S1 and Table 1). The loss of the halide group all together did not significantly change the bioactivity as HSN585 showed only a 2× increase in MIC compared to HSN490 and HSN584 (Figure 4 and Table 1). This result indicated that the halide group was not crucial for binding to the biological target in the selected bacterial strains. However, the replacement of the isoquinoline group with a pyridine (HSN589) resulted in a loss of 50% activity (Figure 4 and Figure S1), indicating the isoquinoline group was vital for potency. The substitution of the phenyl group with a pyridine (HSN736 and HSN740) led to a complete loss of activity (Figure 4 and Figure S1). From our structure activity relationship study, we concluded that the isoquinoline and the phenyl moieties were important for antibacterial activity.

### 2.2. HSN584 and HSN739 Exhibit Broad Spectrum Bactericidal Activity against Gram-Positive Bacteria

Next, we chose three of our most potent compounds (HSN490, HSN584 and HSN739) and screened them against various bacterial strains to examine the spectrum of their antibacterial activity (Table S1). HSN584 and HSN739 exhibited moderate antibacterial activity (MIC range = 4–16 µg/mL) against clinically important Gram-positive bacteria, including *Staphylococcus epidermidis, Listeria monocytogenes*, *Streptococcus pneumoniae, Enterococcus faecalis, Clostridium difficile* and *Enterococcus faecium* (Table S1). Importantly, both HSN584 and HSN739 also exhibited potent (MICs = 4 µg/mL or 8 µg/mL) activity against the drug-resistant strains used in the study, namely, MRSA, VRE *faecium,* VRE *faecalis,* methicillin-resistant *S. pneumoniae,* cephalosporins-resistant *S. pneumoniae,* methicillin-resistant *S. epidermidis* and linezolid-resistant *S. aureus.* HSN490, on the other hand, displayed a weaker antibacterial activity (MIC > 16 µg/mL) against most of the strains except *S. pneumoniae, S. epidermidis* and *Listeria monocytogenes*, against which it exhibited moderate activity (MIC range 4–16 µg/mL) (Table S1). Moreover, the three HSN compounds showed no activity against the wild-type *E. coli* or *tolC*-mutant (strain lacking the AcrAB-TolC multidrug efflux pump) (Table S1). The lack of activity against *tolC*-mutant *E. coli* is an indication our compounds’ lack of activity against *E. coli* is not a result of efflux by the AcrAB-TolC pump, a relevant mechanism of resistance against various clinical antibiotics [26]. The compounds’ minimum bactericidal concentration (MBC) was also determined to examine whether these compounds exert a bactericidal or bacteriostatic activity against the tested bacterial isolates. The compounds’ MBCs were found to be the same as, or one to two-fold higher than, their corresponding MICs, indicating that the compounds are bactericidal agents. In addition, the bactericidal mode of killing of the alkynyl isoquinolines was confirmed via conducting time kill assays (Figure S2). Both HSN584 and HSN739 displayed fast bactericidal activity comparable to vancomycin, the drug of choice for the treatment of MRSA infections. After 2 h, HSN584 completely eradicated the high MRSA USA300 inoculum, while HSN739 resulted in more than 3-log_10_- (99.9%) reduction. 

**Figure 5:**
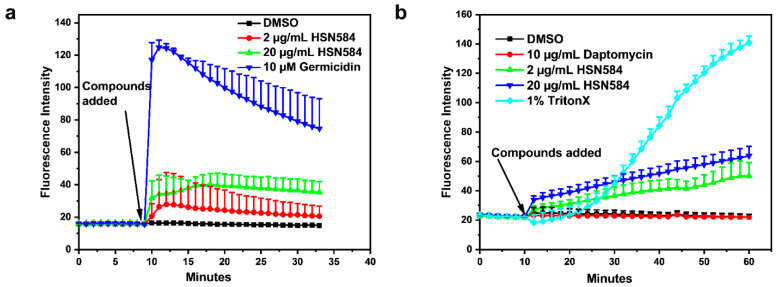
HSN584 effects on *S. aureus* ATCC 25923 membrane integrity. (**a**) HSN584 effects on membrane polarization. Germicidin (positive control); DMSO (negative control). (**b**) HSN584 effects on membrane permeation. A 1% TritonX (positive control); DMSO and Daptomycin (negative controls).

The bactericidal activity of some antibiotics has been correlated with the disruption of membrane integrity, either by degeneration of membrane polarization or via membrane lysing [27,28]. Hence, we investigated the effects of HSN584 on *S. aureus* membrane polarization and permeability. We did not see a significant concentration-dependent enhancement of DISC_3_ or Sytox green fluorescence after treatment with 2 µg/mL and 20 µg/mL of HSN584 (Figure 5). These results indicate that HSN584’s bactericidal activity is not mediated via the disruption of membrane potential or the lysis of the bacterial membrane.

### 2.3. Alkynyl Isoquinolines Are Active against Fluoroquinoline-Resistant Bacteria 

Quinolones derivatives such as ciprofloxacin, levofloxacin and norfloxacin, are one of the largest class of synthetic antibacterial substances used in clinics today [29]. Resistance to fluoroquinolones has been reported to be significantly high in MRSA than in methicillin-sensitive *S. aureus* (MSSA) [30]. Hence, we tested our compounds’ activity against MRSA and vancomycin-resistant *S. aureus* (VRSA) strains, which are also resistant to ciprofloxacin, levofloxacin and norfloxacin. Surprisingly, HSN584 and HSN739 exhibited potent activity (MICs = 4 µg/mL or 8 µg/mL) against fluoroquinolone-resistant *S. aureus* strains (Table 2). HSN490, on the other hand, displayed moderate to weak activity against fluoroquinolone-resistant strains (Table 2).

### 2.4. Alkynyl Isoquinolines Intracellular and In Vivo Effects

One of the main limitations of the conventional antimicrobial agents used in clinics today is the failure to clear intracellular bacteria [16,17]. The ability of an antibacterial compound to clear intracellular bacteria is important because intracellular bacteria are responsible for infection relapse and metastasis [13,14,15,16]. Consequently, we investigated the ability of our lead compounds (HSN584 and HSN739) to clear intracellular bacteria. First, we investigated the HSN584 and HSN739 compounds’ toxicity against murine macrophages (J774 cell line). HSN compounds were non-toxic to the macrophages at concentrations up to 32 µg/mL (Figure S3). We also tested the potential toxicity of HSN584 on *Galleria mellonella* larvae. There was no statistical difference in larvae survival between the groups treated with vehicle (DMSO) and HSN584 after four days. In contrast, the group treated with the chemotherapeutic drug, paclitaxel, had a lower than 30% survival rate after four days (Figure S3B). These findings imply that the isoquinoline moiety could be tolerated in vivo.

**Figure 6:**
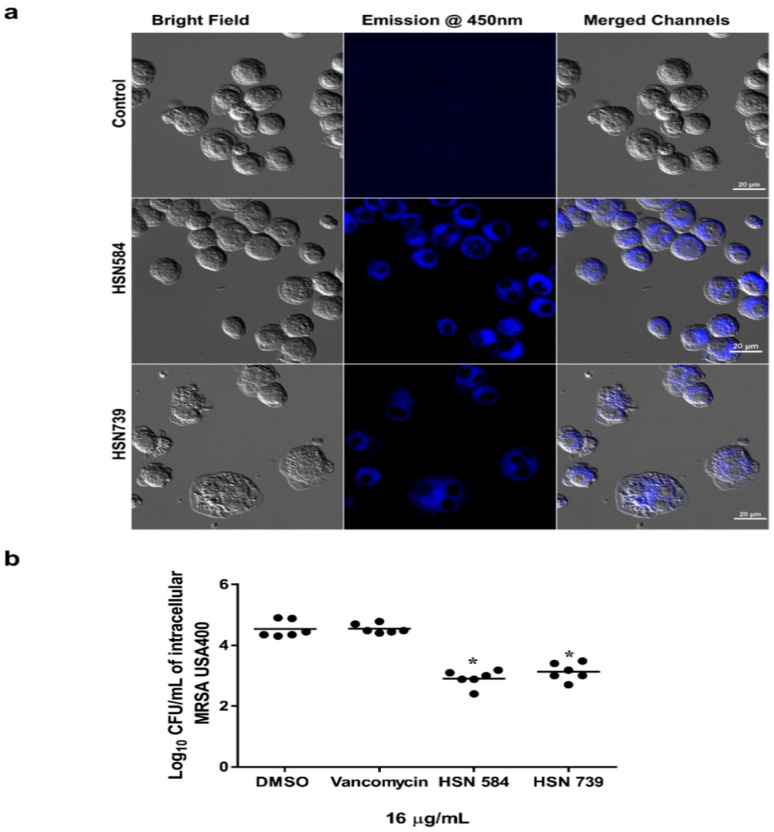
Alkynyl isoquinoline intracellular clearance activity. (**a**) HSN584 and HSN739 permeate murine macrophage cells and localize in the cytoplasm. (**b**) HSN584 and HSN739 significantly reduce MRSA USA400 load in murine macrophage cells after treatment with 16 µg/mL of either drug for 24 h. Data analysis performed with one-way ANOVA with post hoc Dunnett’s test for multiple comparisons. * = *p*-value < 0.05.

Next, we utilized fluorescence microscopy to investigate if the HSN compounds can permeate macrophages. Both our lead molecules are fluorescent; upon excitation at maxima absorbance wavelength (around 400 nm), they emit blue light with maxima emission around 450 nm (Figure S4). Both molecules were found to localize in the cytoplasmic region of the macrophages (Figure 6A). After confirming that the molecules can permeate the macrophage cells, we tested the alkynyl isoquinolines’ efficacy in clearing intracellular MRSA USA 400. We compared our compounds to vancomycin, for a gold standard treatment for MRSA infections. Vancomycin, as reported previously [31,32,33,34], was unable to permeate the infected macrophages and reduce the burden of intracellular MRSA (Figure 6B). On the other hand, HSN584 and HSN739, at the same concentration as vancomycin, showed a significant bacterial burden reduction, an approximately 2 log_10_ reduction (Figure 6B).

### 2.5. S. aureus Does Not Develop Rapid Resistance against HSN584 and HSN789

**Figure 7:**
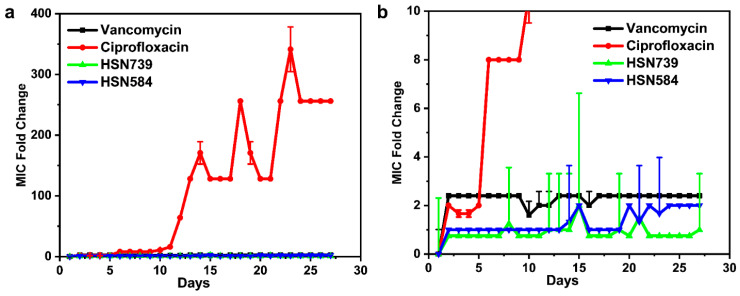
Multistep resistance study. (**a**) Multistep resistance study of vancomycin, ciprofloxacin, HSN584 and HSN739 against *S. aureus* ATCC 25923. (**b**) Magnified version of part a. *S. aureus* was serially passaged over a period of 27 days and the broth microdilution assay was used to determine the MIC of each compound after each successive passage.

The bacterial development of resistance against antibacterial agents is a major contributor to poor patients’ outcome and the antibiotic crisis in which we find ourselves today. Thus, we sought to determine how fast *S. aureus* would develop resistance against the HSN584 and HSN789 via a multi-step resistance selection assay (Figure 7). There was no significant increase in the MICs of vancomycin, HSN584 and HSN739 throughout the 27 days (Figure 7). Only a two-fold increase in the MICs was observed for them throughout the entire study. On the other hand, ciprofloxacin’s MIC increased by 8-fold by day 6, and the MIC continually increased with an observed fold change greater than 200 at the end of the study. Our results are consistent with previous reports that indicate *S. aureus* develop rapid resistance to ciprofloxacin and not vancomycin [35].

### 2.6. Elucidating HSN584 Mechanism of Action

To gain insight into how alkynyl isoquinolines kill bacteria, we investigated the effects of HSN584 on *S. aureus* macromolecule synthesis and global protein expression. The effects on macromolecule synthesis were determined via the quantification of the uptake of isotope labelled macromolecule biosynthesis precursors, i.e., [^3^H] thymidine for DNA synthesis, [^3^H] uridine for RNA synthesis, [^3^H] leucine for protein synthesis and [^3^H] N-acetylglucosamine for cell wall synthesis, in the presence and absence of HSN584. The percent incorporation was calculated with respect to the control group (DMSO). To probe the effects of HSN584 on *S. aureus* proteome, cells were incubated with 1 µg/mL HSN584 or DMSO for 2 h at 37 °C, lysed and a comparative global proteomics analysis conducted.

**Figure 8:**
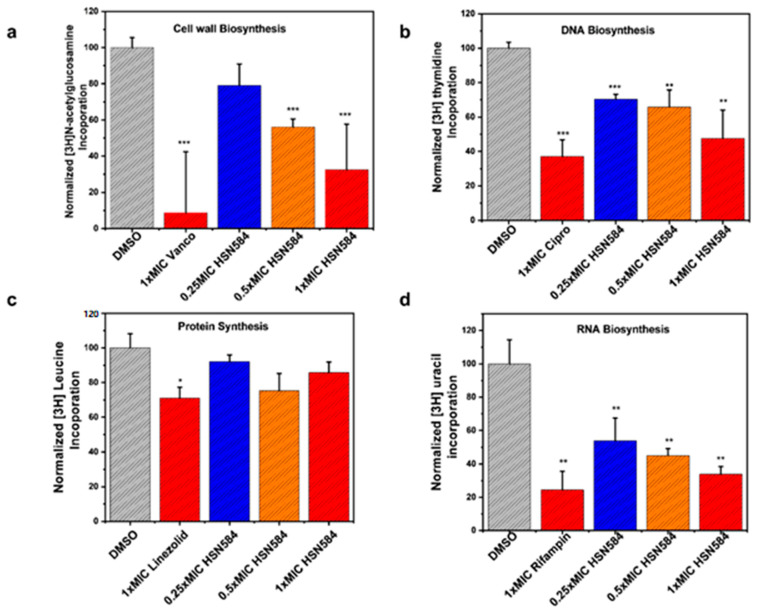
HSN584 effects on *S. aureus* ATCC 25923 macromolecule synthesis. (**a**) HSN584 effects on [^3^H] N-acetylglucosamine incorporation into *S. aureus* cell wall. (**b**) HSN584 effects on [^3^H] thymidine incorporation into *S. aureus* DNA. (**c**) HSN584 effects on [^3^H] leucine incorporation into *S. aureus* protein. (**d**) HSN584 effects on [^3^H] uridine incorporation into *S. aureus* RNA. Student’s *t*-test was used for statistical analysis. * *p*-value < 0.05, ** *p*-value < 0.01, *** *p*-value < 0.001.

For the macromolecule biosynthesis experiment, *S. aureus* cells were treated with three different HSN584 concentrations (0.25 × MIC, 0.5 × MIC and 1 × MIC (MIC = 2 µg/mL)). HSN584 exhibited a concentration-dependent inhibition of the cell wall, DNA and RNA synthesis (Figure 8a,b,d). However, HSN584 did not exhibit a concentration-dependent inhibition of protein synthesis (Figure 8c). For the cell wall biosynthesis, HSN584 significantly inhibited the incorporation of [^3^H] *N*-acetylglucosamine only at 0.5 × MIC and 1 × MIC concentrations. On the other hand, HSN584 significantly inhibited the biosynthesis of DNA and RNA at all the three concentrations tested. To confirm the precursors’ incorporation was, indeed, a result of the HSN584 interference of the respective biosynthetic pathways and not because of the reduced cell viability, we incubated the cells under the same conditions and measured the cell viability (Figure S5). For DNA and RNA biosynthesis, cells were exposed to the antibacterial agents for 20 min. This exposure did not significantly affect the cells’ viability even at high concentrations (Figure S5a), suggesting the reduction in the incorporation of the radiolabeled precursors was, indeed, due to interference with the biosynthetic pathways. For the cell wall biosynthesis, cells were exposed to the antibacterial agents for 1 h. Cell viability was only significantly affected at high concentrations (8 × MIC) (Figure S5b). Since HSN584 exhibited a dose-dependent reduction in [^3^H] *N*-acetylglucosamine incorporation at lower concentrations (Figure 8a), we can conclude HSN584 interferes with cell wall biosynthesis. For protein synthesis, cells were exposed to the antibacterial agents for 1.5 h. This exposure significantly affected cell viability at all concentrations, except for the 1 × MIC HSN584 group (Figure S5c). Since there was no concentration-dependent inhibition of protein synthesis, we concluded our compound does not affect de novo protein synthesis.

**Figure 9:**
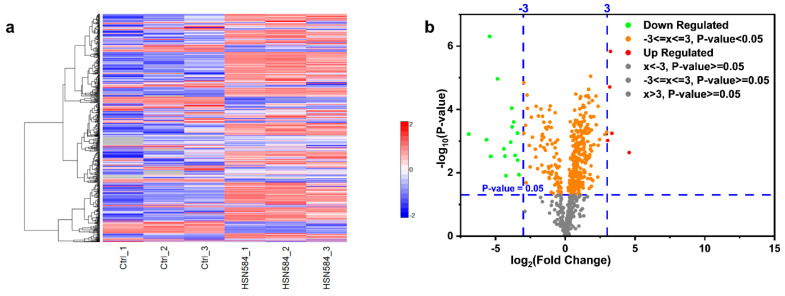
HSN584 effects on *S. aureus* ATCC 25923 protein expression. (**a**) Heat map depicting the expression of *S. aureus* proteins in the absence and presence of HSN584. (**b**) Visualization of *S. aureus* proteins regulation by HSN584 via a volcano plot.

**Figure 10:**
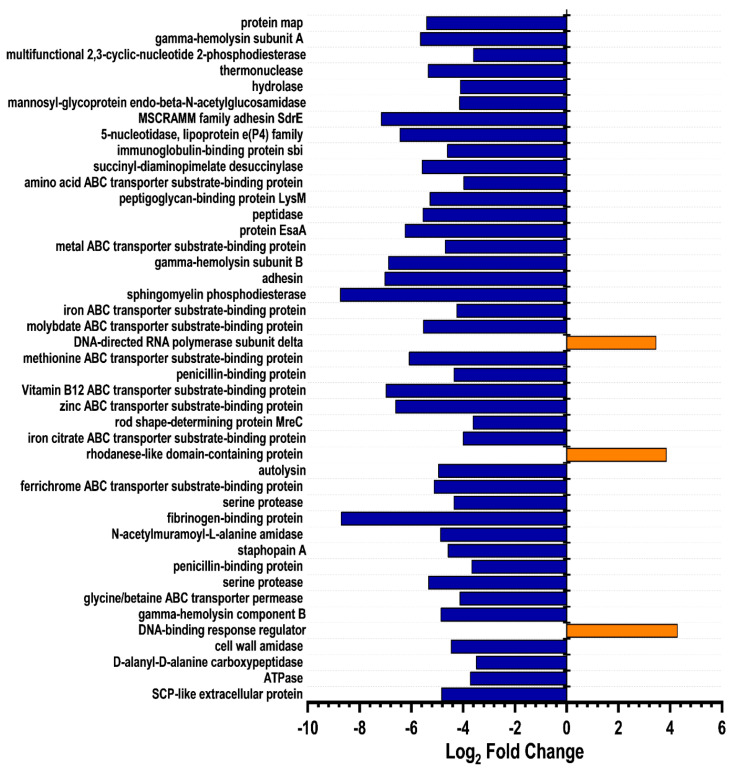
*S. aureus* ATCC 25923 proteins regulated by a log_2_ fold greater than 3 in response to HSN584 treatment.

HSN584 was found to differentially regulate *S. aureus* protein expression (Figure 9a,b). To narrow down the crucially regulated biological processes by HSN584 treatment, we focused on two groups of proteins: unique proteins and significantly regulated proteins, with log_2_ fold change greater than 3.5. Unique proteins are those proteins present in only one sample group. Most of the control group’s unique proteins were ATP-binding cassette (ABC) transporters (Table 3). Additionally, several ABC proteins appeared in the list of significantly regulated proteins (Figure 10). ABC transporters function either as importers, bringing nutrients or exporters that pump toxins or lipids across the membrane. ABC transporters have been implicated in antibiotic resistance (involved in efflux of antibiotics) and bacterial virulence (required for uptake of nutrients that are crucial for bacteria colonization) [36]. Hence, the inhibition of ABC transporters can be detrimental for the cell. One interesting ABC transporter affected by HSN584 is the osmoprotectant ABC transporter (Table 3), which protects *S. aureus* against the damaging effects of high salt concentration environments. *S. aureus* has ability to survive high-salt conditions, a characteristic that enables it to colonize the human skin [37]. To validate our proteomics data, we hypothesized that the killing effect of HSN584 will be potentiated in the presence of salts (e.g., NaCl) due to the inhibition of the osmoprotectant ABC transporter. We investigated the ability of *S. aureus* to grow in different concentrations of NaCl and in the presence and absence of HSN584. In the absence of NaCl, significant growth inhibition was only evident in cells treated with 4 µg/mL HSN584. On the other hand, we saw a correlation between the lowest concentration of HSN584 that exhibited a significant growth inhibition and NaCl concentration. In the group supplemented with 0.8 M NaCl the lowest HSN584 concentration that exhibited significant growth inhibition was 1 µg/mL whilst in 2 M NaCl group there was a significant growth inhibition at 0.25 µg/mL HSN584 (Figure S6).

Another interesting group of proteins downregulated by HSN584 treatment are the cell wall (CW) hydrolases, namely, N-acetylmuramoyl-L-alanine amidase, mannosyl-glycoprotein endo-beta-*N*-acetylglucosamidase and peptidase M23B (Figure 10). During growth and cell division, the peptidoglycan (PG) is actively remodeled to allow the expansion of the PG and the accommodation of cell shape changes, a process that is facilitated by the CW hydrolases [38]. Consequently, CW hydrolases are important for cell wall maturation. CW amidases hydrolyze the amide bond connecting the glycan chain and the stem peptide, while CW peptidases cleave the amide bond between amino acids in the stem peptide [38]. We also observed the downregulation of penicillin-binding proteins (PBPs) d-alanyl-d-alanine carboxypeptidase and two other PBPs (Figure 10). PBPs are transpeptidase enzymes, which are involved in the cross linking of peptides, which is the final step of PG synthesis. The downregulation of CW hydrolases and PBPs could affirm our macromolecular biosynthesis results, which show HSN584 affects the incorporation of the isotope labelled [^3^H] *N*-acetylglucosamine into the PG. However, according to the proteomics data, this effect is likely from the inhibition of PG maturation rather than the inhibition of UDP-[^3^H] *N*-acetylglucosamine (UDP-GlcNAC), which happens in the first step of PG biosynthesis.

Next, we looked at the expression of proteins involved in nucleic acid metabolism to see if our proteomics data could explain the inhibition of DNA and RNA biosynthesis exhibited by HSN584. Several proteins involved in DNA metabolism were overexpressed in the treated group, namely, nuclease SbcCD subunit C, DNA mismatch repair protein MutT, DNA mismatch repair protein MutL, DNA repair protein RadA, Holliday junction resolvase RuvX, Holliday junction branch migration protein RuvA and transcription-repair coupling factor (Table 4). The nuclease SbcCD subunit C cleaves DNA hairpin structures, which are known to halt DNA replication [39]. The overexpression of nuclease SbcCD subunit C upon treatment with HSN584 could be a mechanism by which the cell adapts to counteract HSN584’s inhibitory effects on DNA replication. MutT repairs mismatch during replication, while MutL is part of a methyl-directed mismatch repair system that repairs post-replicative errors [40]. The DNA repair protein RadA, Holliday junction resolvase RuvX and Holliday junction branch migration protein RuvA are all involved in homologous recombination. Homologous recombination is a vital pathway for DNA damage repair. Transcription-repair coupling factor terminates transcription at site of stalled RNA polymerase and recruit nucleotide excision repair (NER) to the site of the damage [41,42]. The upregulation of a transcription-repair coupling factor could explain the downregulation of RNA biosynthesis as cells are known to shut down transcription as a response mechanism to the presence of transcription-blocking DNA lesions [43].

Furthermore, several virulence factors were downregulated by HSN584 treatment, including staphopain B, protein map, MSCRAMM (microbial surface components recognizing adhesive matrix molecules) family adhesin SdrE, gamma-hemolysin and sphingomyelinase (Figure 10 and Table 3). These virulence factors allow bacterial cells to adhere to host surfaces, invade host, circumvent host immune system, and induce cytotoxicity to host cells. Staphopain B and protein map proteins have been shown to regulate immune response [44,45,46]. Gamma-hemolysin and sphingomyelinase are membrane-damaging exotoxins that facilitate *S. aureus* pathogenesis [47].

### 2.7. HSN584 Synergizes with Trimethoprim against Trimethoprim-Resistant MRSA

The combination of two or more drugs for the treatment of bacterial infections has been adapted as an alternative treatment for bacterial infections. Combinational therapy advantages include the prevention of resistance emergence, lower toxicity, and more rapid antibacterial activity [48]. Therefore, we sought to find if HSN584 synergizes with any known antibacterial agent against MRSA. Using a checkerboard assay, we examined HSN584 interactions with 19 antibacterial agents against MRSA ATCC 33592. Synergy was determined by calculating sum of fractional inhibitory concentrations (εFIC) [49]. HSN584 did not exhibit any antagonistic interactions with any of the antibacterial agents (εFIC < 2). Interestingly, it exhibited a synergistic effect with trimethoprim (εFIC < 0.28) (Table S2, Figure S7). In the presence of 1 µg/mL of HSN584, trimethoprim MIC dropped from >128 µg/mL to 4 µg/mL.

## 3. Materials and Methods

The bacterial strains used in the study were obtained from the American Type Culture Collection (ATCC) or the Biodefense and Emerging Infections Research Resources Repository (BEI Resources), Table 2.

### 3.1. Screening of Isoquinoline Compounds for Antibacterial Activity against S. aureus

Bacterial cells, at the exponential phase of growth, were incubated overnight at 37 °C, with 16 µg/mL of lead compounds and DMSO for the control. Then, 100 µL aliquots were transferred to a 96-well microtiter plate and optical density (*OD*_600_) was measured. Experiments were performed in triplicates. The percent growth inhibition was calculated as follows:

(1) $$\%Normalized\;OD_{600}=\left(\frac{X_{T}-X_{o}}{X_{c}-X_{o}}\right)\times 100$$

where *X_T_* is the *OD*_600_ of culture with the compound, $X_{o}$ is that of media only and $X_{C}$ is the *OD*_600_ of the DMSO control.

### 3.2. Determination of the MICs and MBCs against Clinically Important Gram-Positive Bacteria

The antibacterial activity of the HSN compounds was evaluated using the broth microdilution method as outlined previously [50,51,52,53,54]. Briefly, a 0.5 McFarland standard solution from each strain was prepared and diluted in cation-adjusted Mueller–Hinton broth to reach a concentration of ~5 × 10^5^ CFU/mL. *C. difficile* was diluted in BHIS. Serial dilutions of test agents were incubated with bacteria before recording the MICs. MBCs were determined by spotting the wells showed no growth onto tryptic soy agar (TSA) plates. The MBC was determined as the lowest concentration that resulted in 99.9% reduction in bacterial growth [55,56,57].

### 3.3. HSN584 Effects on Membrane Polarization and Membrane Permeation

A membrane polarization assay was conducted as previously described [58] with some modifications. Briefly, *S. aureus* ATCC 25923 cells were grown to exponential phase using TSB media, harvested by centrifugation, and resuspended in 5 mM HEPES, pH 7.2 supplemented with 5 mM glucose to *OD*_600_ = 0.05. Cells were then treated with 0.5 µM cyanine dye DiS-C3-(5) (Tokyo Chemical Industry), followed by 15 min incubation at room temperature. Cells were then treated with 100 mM KCl. A total of 100 µL of the cell suspension was aliquoted into a 96-well black plate; fluorescence emission (λ_ex_ 622 nm, λ_em_ 670 nm) was tracked for 10 min before addition of the control and test compounds. For membrane permeation, assays were conducted as previously reported [59,60] with slight modifications. Briefly, 10 mL culture of *S. aureus* ATCC 25923 was grown to exponential phase using TSB media, washed 3× with 1× PBS and resuspended in 10 mL of 1× PBS supplemented with 10% TSB media. Cells were treated with 5 µM SYTOX^TM^ Green nucleic acid (Invitrogen (Thermo Fisher), Waltham, MA, USA), before 100 µL of the cell suspension was aliquoted into a 96-well black plate. Fluorescence emission (λ_ex_ 504 nm, λ_em_ 522 nm) was tracked for 10 min before the addition of the control and test compounds. Fluorescence measurements were obtained using Biotek Cytation5 image reader (Biotek (now Agilent), Santa Clara, CA, USA).

### 3.4. In Vitro Cytotoxicity Analysis of HSN Compounds against J774 Cells

Cytotoxicity assessment for HSN compounds was determined against a murine macrophage J 774 cell line (ATCC, Manassas, VA, USA), as described previously [33,34,61]. Briefly, cells were incubated with the compounds (in triplicates) for 24 h. DMSO was included as a control. MTS reagent (Promega, Madison, WI, USA) was added and incubated for four hours, and the *OD*_490_ was measured. A one-way ANOVA, with post hoc Dunnett’s test (*p* < 0.05), was employed for data analysis.

### 3.5. Florescent Imaging

Murine macrophage (J774) cells were seeded in a glass bottom 15 mm Petri dish and grown to 80% confluence. Cells were grown in DMEM supplemented with 10% FBS. Cells were then incubated in medium supplemented with 8 µg/mL HSN584 or HSN739 and incubated for 2 h at 37 °C. After 2 h, the medium was removed, and cells washed three times with 1× PBS. Cells were incubated in 16% formalin for 1 h at room temperature. Formalin solution was then removed, and cells washed three times with 1× PBS. Then, cells were covered with 1× PBS for imaging. Before imaging, absorbance spectra for HSN584 and HSN739 were obtained with Biotek Cytation5 image reader. Excitation at the highest absorbance wavelength was then used to generate emission spectra for both compounds (Figure S4). Images were captured with a Nikon Ti microscope with a plan Apo 20 × DIC M N2 objective lens at Purdue Bioscience Imaging Facility.

### 3.6. Examination of Clearance of Intracellular MRSA Present in Murine Macrophage (J774) Cells

The intracellular clearance activity of isoquinoline compounds was evaluated as described earlier [62,63]. J774 cells were infected with MRSA USA 400 (10:1) for 60 min. Thereafter, the cells were washed with gentamicin to remove extracellular MRSA. Then, HSN compounds and vancomycin (16 µg/mL) were added (in triplicates) and incubated for 24 h. Afterwards, cells were washed and lysed with 0.1% Triton-X before dilution and plating. A one-way ANOVA, with post hoc Dunnett’s test (*ρ* < 0.05), was employed for data analysis.

### 3.7. Resistance Selection

A multi-step resistance selection experiment as described elsewhere [57,64,65] was used to assess *S. aureus* ATCC 25923’s ability to develop resistance against the alkynyl isoquinolines (HSN 584 and HSN 739), vancomycin and ciprofloxacin. MIC was determined daily over a course of 27 days using the broth microdilution method.

### 3.8. Macromolecule Synthesis Assay

To measure macromolecule biosynthesis, cells were incubated in TSB supplemented with HSN584 or control compounds (ciproflaxicin (DNA), vancomycin (cell wall), rifampicin (RNA) and linezolid (proteins)) for 10 min (DNA and RNA) or 30 min for cell wall and 1.5 h for protein biosynthesis. A total of 0.5µCi total concentration of radiolabeled macromolecule synthesis precursors ([^3^H] thymidine (DNA), [^3^H] uridine (RNA), [^3^H] leucine (protein) and [^3^H] N-acetylglucosamine (cell wall)) were added to the culture media and cells incubated further at 37 °C. For DNA, RNA and cell wall biosynthesis, cells were incubated for an additional 10 min with the radiolabeled precursors. For cell wall, cells were incubated with [^3^H] N-acetylglucosamine for 30 min. For protein synthesis, cells were incubated with [^3^H] leucine for 1 h. The reaction was stopped by adding equal volume of 10% trichloroacetic acid. Cells were incubated on ice for 30 min to precipitate the macromolecules. The precipitate was then collected via vacuum filtration and washed three times with 5% trichloroacetic acid. Filter papers were dried on a 95 °C hotplate. The experiment was performed in triplicates. Then, filter paper was then transferred to a scintillation vial and after the addition of scintillation liquid, activity was determined with a liquid scintillation counter.

### 3.9. S. aureus Growth in the Presence of High Salt Concentration

Exponentially growing cells were transferred to TSB media supplemented with different concentrations of NaCl and HSN 584. Bacterial growth was monitored by measuring the optical density as described in the antibacterial screening section. 

### 3.10. Global Proteomics

Exponentially growing *S. aureus* ATCC 25923 cells were treated with HSN584 (1 µg/ML) or DMSO for 2 h at 37 °C. Cells were then washed three times with 1× PBS. Soluble proteins were first extracted by suspending the pellet in 500 µL of 20 Mm Tris-HCl buffer containg 100 mM NaCl, 1 mM EDTA, 5% glycerol and 0.5 mM DTT. The cells were transferred to lysis tubes and lysed using Precellys^®^ 24 Bead Mill Homogenizer (Bertin Corp., Rockville, MD, USA). The lysate was centrifuged at 14,000 rpm for 5 min at 4 °C. The supernatant that contained the soluble proteins was transferred to a clean tube. For the extraction of insoluble proteins, the pellet was suspended with 8M urea and vortexed for 2 h at room temperature. The sample preparation for insoluble proteins proceeded as outlined in our previous publication [66]. For soluble proteins, we skipped the precipitation step and quantified protein concentration of the supernatants. Reduction, acetylation, digestion and column steps were the same for both soluble and insoluble samples. LC-MS/MS acquisition and data analysis were conducted as described in our previous publication [66]. However, Inferno software was used to generate the heat map and origin software used to generate the volcano plot.

### 3.11. Checkerboard Assay

The checkerboard assay [67,68] was used to determine antibiotics compounds interactions against MRSA ATCC 33592. Nineteen antibiotics were tested in combination with HSN584. Fractional inhibitory concentration indices were calculated for each interaction as follows:

(2) $$FIC_{HSN584/Antibiotic}=\frac{HSN584/\left(Antibiotic\right)MIC\;in\;combination}{HSN584\left(Antibiotic\right)MIC\;Alone}$$

The cumulative *FIC* (∑*FIC*) was then calculated as:

(3) $$\varepsilon FIC=FIC_{compound}+FIC_{antibiotic}$$

Interactions where the ∑*FIC* was ≤0.5 were categorized as synergistic [69].

### 3.12. Galleria Mellonella Toxicity Assay

Galleria mellonella (greater wax moth) larvae shown to be a good in vivo model for compound toxicity assay [70] were obtained commercially (Speedy Worm, Alexandria, MN, USA) and maintained in sawdust in a dark cabinet at room temperature until use in the following day. Larvae were weighed and those weighing about 250 ± 50 mg were used. Any larvae showing signs of darkening or black discoloration were discarded. Ten larvae were selected for each treatment condition or control and placed in a Petri dish using blunt edged forceps. We labelled separate Petri dishes with individual treatment conditions and placed 90 mm filter papers in the 100 mm Petri dishes to serve as a bed for the larvae. A flat Styrofoam surface covered with filter paper was used as a platform for injections. We carefully injected 10 μL of the test compounds formulated in DMSO into the hemocoel of the larvae through the last left proleg using a 10 μL Hamilton glass syringe (801RN, Hamilton, Reno, NV, USA) fitted with a 30G needle and returned the larvae to the filter paper lined Petri dish. Control larvae were injected with 10μL DMSO. After injections, Petri dishes were kept in a perforated box in the dark for 4 days at room temperature. Survival was evaluated daily by checking for movement in response to prodding with blunt forceps. Darkened or shriveled larvae or those that did not survive the treatments were discarded by freezing at −20 °C overnight and autoclaved in a biohazard leak proof container the following day [71].

## 4. Conclusions

The continuous emergence of antibiotic resistance highlights the need for development of new antibacterial agents to evade the probable post-antibiotic era. The discovery of novel scaffolds with antibacterial activity is vital for successfully managing multidrug-resistant bacterial infections. To this end, we screened our alkynyl isoquinoline chemical library aiming at finding novel active antibacterial agents. We identified alkynyl isoquinoline with potent activity against clinically important Gram-positive bacteria. We showed that the lead compounds can significantly reduce the burden of intracellular MRSA and discovered that *S. aureus* exhibited a low potential for resistance development against our lead compounds. Our preliminary studies showed that the alkynyl isoquinoline molecule exerted its antibacterial activity through inhibiting three major bacterial processes, DNA synthesis, RNA synthesis and cell wall synthesis. In our future studies, we will seek to evaluate the exact mechanisms by which the alkynyl isoquinoline inhibit these processes. Our findings highlight alkynyl isoquinoline scaffolds as ideal lead molecules for the discovery of potent antibacterial agents.

## Figures and Tables

**Table 1:** HSN490 analogs’ minimal growth inhibition concentrations (MICs) against *S. aureus*, MRSA, vancomycin-sensitive *E. faecalis* and VRE *faecalis*.

	*S. aureus* ATCC 25923 (µg/mL)	MRSA ATCC 33592 (µg/mL)	MRSA USA 300-0114 (µg/mL)	*E. faecalis* (µg/mL)	VRE (µg/mL)
HSN584	2	2	2	2	2
HSN738	8	8	4	2	8
HSN739	2	2	2	1	2
HSN585	4	4	4	4	4

**Table 2:** The minimum inhibitory concentrations (MICs in µg/mL) and minimum bactericidal concentrations (MBCs in µg/mL) of HSN compounds against fluoroquinolone-resistant *S. aureus* isolates.

Bacterial Strains	Compounds/Control Drugs													
	HSN 490		HSN 584		HSN 739		Linezolid		Ciprofloxacin		Levofloxacin		Norfloxacin	
	MIC	MBC	MIC	MBC	MIC	MBC	MIC	MBC	MIC	MBC	MIC	MBC	MIC	MBC
MRSA NRS 385 (USA 500)	8	16	4	4	8	8	2	32	>128	>128	32	32	>128	>128
MRSA NRS 383 (USA 200)	16	32	8	8	8	8	2	32	128	128	32	32	>128	>128
MRSA NRS 382 (USA 100)	16	32	4	4	8	8	2	64	32	32	16	16	128	128
VRSA 4	16	16	4	4	8	8	1	16	128	128	32	32	>128	>128
VRSA 7	16	16	4	4	4	4	1	16	128	128	32	32	>128	>128
VRSA 8	16	32	4	4	4	4	0.5	16	128	128	32	32	128	128
VRSA 9	16	16	4	8	4	8	1	64	>128	>128	32	32	>128	>128
VRSA 10	16	16	4	8	8	8	1	64	>128	>128	32	64	>128	>128
VRSA 13	16	32	4	4	4	4	1	64	128	128	16	16	>128	>128

**Table 3:** Control group unique proteins.

Protein Name	Average MS/MS Count
DUF5067 domain-containing protein	73
Multidrug transporter	38
Nickel ABC transporter	35
Peptide ABC transporter substrate-binding protein	30
Phosphonate ABC transporter substrate-binding protein	23
Osmoprotectant ABC transporter substrate-binding protein	21
Tandem-type lipoprotein	19
Staphopain B	17
Protein EssB	14
Gamma-hemolysin component C	13
Glycerophosphodiester Phosphodiesterase	11
Peptidase M23B	10
Iron ABC transporter substrate-binding protein	10
Teichoic acid ABC transporter ATP-binding protein	7
Polysaccharide deacetylase	7
Sodium ABC transporter permease	6
Cell division protein FtsQ	6
Peptide ABC transporter permease	5
Nitrate ABC transporter substrate-binding protein	5

**Table 4:** Treated group’s unique proteins.

Protein Name	Average MS/MS Count
Ribosome silencing factor RsfS	28
DNA mismatch repair protein MutT	13
XRE family transcriptional regulator	13
Mevalonate kinase	12
Ribosome maturation factor	12
DNA repair protein RadA	12
Shikimate dehydrogenase	12
Betaine-aldehyde dehydrogenase	12
Dihydroxyacetone kinase subunit L	11
Glycine cleavage system protein H	11
Endoribonuclease YbeY	11
Molybdoprotein synthase sulfur carrier subunit	11
Phosphomevalonate kinase	10
Cell division protein FtsK	10
Transcriptional-repair coupling factor	10
tRNA pseudouridine(55) synthase TruB	10
Ketol-acid reductoisomerase	10
Two-component sensor histidine kinase	10
HAD family hydrolase	10
FAD-dependent oxidoreductase	9
Signal peptidase II	9
Type II secretion protein	9
3-dehydroquinate synthase	9
DNA polymerase/3-5 exonuclease PolX	9
Polyisoprenoid-binding protein	9
Cytidine deaminase	9
Acylphosphatase	9
Initiation-control protein YabA	9
Exodeoxyribonuclease 7 large subunit	8
Isochorismate synthase	8
LytTR family transcriptional regulator	8
Cell division protein ZapA	8
Nuclease SbcCD subunit C	7
Holliday junction resolvase RuvX	7
3-phosphoshikimate 1-carboxyvinyltransferase	7
Holliday junction branch migration protein RuvA	7
DNA methyltransferase	7
DNA mismatch repair protein MutL	6

## Data Availability

The raw LC-MS/MS data are deposited in the Mass Spectrometry Interactive Virtual Environment (http://massive.ucsd.edu, accessed on 14 July 2022) with the ID MSV000086953.

## Supplementary Materials

The following supporting information can be downloaded at: https://www.mdpi.com/article/10.3390/molecules27165085/s1, Figure S1: HSN490 analogs effects on inhibition of bacterial growth. *S. aureus* cell viability after treatment with 16 μg/mL HSN490 analogs; Figure S2: Time kill profiles. (a) HSN584 time kill profile against MRSA USA 300. (b) HSN739 time kill profile against MRSA USA 300. Vancomycin was used as a positive control and DMSO (compounds’ solvent) as a negative control. Figure S3: Alkynyl Isoquinolines cytotoxicity and in vivo toxicity. (a) HSN490, HSN584 and HSN739 cytotoxicity against murine macrophages (J774 cell line). (b) Galleria mellonella survival curves for HSN584. Galleria worms dosed at 10 mg/Kg with HSN584 and other compounds, survival monitored over 4 days. Figure S4: HSN584 and HSN739 absorbance spectrum and emission spectrum upon excitation at 400 nm Figure S5: Positive controls and HSN584 effects on *S. aureus* ATCC 25923 cell viability. (a) Cells treated for 20 min with respective antibacterial agents. (Cipro = ciproflaxicin) MIC Cipro = 0.125 μg/mL, MIC rifampicin = 7.8 ng/mL, MIC HSN584 = 2 μg/mL. (b) Cells were treated for 1 h with respective antibacterial agents. MIC vancomycin = 2 μg/mL. (c) Cells were treated for 1.5 h with respective antibacterial agents. MIC linezolid = 2 μg/mL. Experiment was done in triplicate. The data were analyzed via the Student’s *t*-test (*p* < 0.05). Asterisks (*) denote statistical significant difference of the corresponding treatments as compared to the DMSO (negative control) treatment; Figure S6: HSN584 effects on S. aureus ATCC 25923 growth in presence of different concentrations of NaCl. Student’s *t*-test was used for statistical analysis. * *p*-value < 0.05, ** *p*-value < 0.01, *** *p*-value < 0.001. Figure S7: HSN584 synergizes with Trimethoprim against methicillin-resistant *S. aureus* ATCC 33592; Table S1: Evaluation of alkynyl Isoquinolines against variety of bacterial isolates. Minimum inhibitory concentrations (MICs in μg/mL) and minimum bactericidal concentrations (MBCs in μg/mL) of the compounds/control drugs against methicillin-resistant Staphylococcus aureus (MRSA), Staphylococcus epidermidis, Streptococcus pneumoniae, Enterococcus faecium, Enterococcus faecalis, Listeria monocytogenes, Escherichia coli, and Clostridium difficile isolates; Table S2: Individual and combination MICs of several commercial antibacterial agents and HSN584 against MRSA ATTC.

## Sample Availability

Samples of the compounds HSN584 and HSN739 are available from the authors. Contact hsintim@purdue.edu for samples.

## Abbreviations

DMSO (Dimethyl sulfoxide), LC-MS/MS (Liquid Chromatography with tandem mass spectrometry), ANOVA (Analysis of variance), DMEM (Dulbecco’s Modified Eagle Medium), DNA (Deoxyribonucleic acid), RNA (Ribonucleic acid).

## References

[1] Naclerio G.A., Sintim H.O. (2020). Multiple ways to kill bacteria via inhibiting novel cell wall or membrane targets. Future Med. Chem..

[2] Laxminarayan R., Van Boeckel T., Frost I., Kariuki S., Khan E.A., Limmathurotsakul D., Larsson D.G.J., Levy-Hara G., Mendelson M., Outterson K. (2020). The Lancet Infectious Diseases Commission on antimicrobial resistance: 6 years later. Lancet Infect. Dis..

[3] Appelbaum P.C. (2012). 2012 and beyond: Potential for the start of a second pre-antibiotic era?. J. Antimicrob. Chemother..

[4] Centers for Disease Control and Prevention (CDC) (2013). Antibiotic Resistance Threats in the United State.

[5] Fair R.J., Tor Y. (2014). Antibiotics and Bacterial Resistance in the 21st Century. Perspect. Medicinal Chem..

[6] Ventola C.L. (2015). The Antibiotic Resistance Crisis: Part 1: Causes and Threats. Pharm. Ther..

[7] Tacconelli E., Carrara E., Savoldi A., Harbarth S., Mendelson M., Monnet L.D., Pulcini C., Kahlmeter G., Kluytmans J., Carmeli Y. (2018). Discovery, research, and development of new antibiotics: The WHO priority list of antibiotic-resistant bacteria and tuberculosis. Lancet Infect. Dis..

[8] Fernandes P. (2006). Antibacterial discovery and development—the failure of success?. Nat. Biotechnol..

[9] Fernandes P., Martens E. (2017). Antibiotics in late clinical development. Biochem. Pharmacol..

[10] Coates A.R.M., Halls G., Hu Y. (2011). Novel classes of antibiotics or more of the same?. Br. J. Pharmacol..

[11] Watkins R.R., Lemonovich T.L., File T.M. (2012). An evidence-based review of linezolid for the treatment of methicillin-resistant Staphylococcus aureus (MRSA): Place in therapy. Core Evid..

[12] Gu B., Kelesidis T., Tsiodras S., Hindler J., Humphries R.M. (2013). The emerging problem of linezolid-resistant Staphylococcus. J. Antimicrob. Chemother..

[13] Thwaites G.E., Gant V. (2011). Are bloodstream leukocytes Trojan Horses for the metastasis of Staphylococcus aureus?. Nat. Rev. Microbiol..

[14] Sandberg A., Hessler J.H.R., Skov R.L., Blom J., Frimodt-Møller N. (2009). Intracellular Activity of Antibiotics against *Staphylococcus aureus* in a Mouse Peritonitis Model. Antimicrob. Agents Chemother..

[15] Gresham H.D., Lowrance J.H., Caver T.E., Wilson B.S., Cheung A.L., Lindberg F.P. (2000). Survival of *Staphylococcus aureus* Inside Neutrophils Contributes to Infection. J. Immunol..

[16] Lehar S.M., Pillow T., Xu M., Staben L., Kajihara K.K., Vandlen R., DePalatis L., Raab H., Hazenbos W.L., Hiroshi Morisaki J. (2015). Novel antibody–antibiotic conjugate eliminates intracellular S. aureus. Nature.

[17] Barcia-Macay M., Seral C., Mingeot-Leclercq M.-P., Tulkens P.M., Van Bambeke F. (2006). Pharmacodynamic evaluation of the intracellular activities of antibiotics against Staphylococcus aureus in a model of THP-1 macrophages. Antimicrob. Agents Chemother..

[18] Xiong M.-H., Li Y.-J., Bao Y., Yang X.-Z., Hu B., Wang J. (2012). Bacteria-Responsive Multifunctional Nanogel for Targeted Antibiotic Delivery. Adv. Mater..

[19] O’Connell K.M.G., Hodgkinson J.T., Sore H.F., Welch M., Salmond G.P.C., Spring D.R. (2013). Combating Multidrug-Resistant Bacteria: Current Strategies for the Discovery of Novel Antibacterials. Angew. Chem. Int. Ed..

[20] Larocque E.A., Naganna N., Opoku-Temeng C., Lambrecht A.M., Sintim H.O. (2018). Alkynylnicotinamide-Based Compounds as ABL1 Inhibitors with Potent Activities against Drug-Resistant CML Harboring ABL1(T315I) Mutant Kinase. ChemMedChem.

[21] Larocque E., Naganna N., Ma X., Opoku-Temeng C., Carter-Cooper B., Chopra G., Lapidus R.G., Sintim H.O. (2017). Aminoisoquinoline benzamides, FLT3 and Src-family kinase inhibitors, potently inhibit proliferation of acute myeloid leukemia cell lines. Future Med. Chem..

[22] Ma X., Zhou J., Wang C., Carter-Cooper B., Yang F., Larocque E., Fine J., Tsuji G., Chopra G., Lapidus R.G. (2017). Identification of New FLT3 Inhibitors That Potently Inhibit AML Cell Lines via an Azo Click-It/Staple-It Approach. ACS Med. Chem. Lett..

[23] Scott J.D., Williams R.M. (2002). Chemistry and Biology of the Tetrahydroisoquinoline Antitumor Antibiotics. Chem. Rev..

[24] Galán A., Moreno L., Párraga J., Serrano Á., Sanz M.J., Cortes D., Cabedo N. (2013). Novel isoquinoline derivatives as antimicrobial agents. Bioorg. Med. Chem..

[25] Panchaud P., Bruyère T., Blumstein A.-C., Bur D., Chambovey A., Ertel E.A., Gude M., Hubschwerlen C., Jacob L., Kimmerlin T. (2017). Discovery and Optimization of Isoquinoline Ethyl Ureas as Antibacterial Agents. J. Med. Chem..

[26] Pérez A., Poza M., Fernández A., del Carmen Fernández M., Mallo S., Merino M., Rumbo-Feal S., Cabral M.P., Bou G. (2012). Involvement of the AcrAB-TolC Efflux Pump in the Resistance, Fitness, and Virulence of *Enterobacter cloacae*. Antimicrob. Agents Chemother..

[27] Tyagi P., Singh M., Kumari H., Kumari A., Mukhopadhyay K. (2015). Bactericidal Activity of Curcumin I Is Associated with Damaging of Bacterial Membrane. PLoS ONE.

[28] Silverman J.A., Perlmutter N.G., Shapiro H.M. (2003). Correlation of daptomycin bactericidal activity and membrane depolarization in Staphylococcus aureus. Antimicrob. Agents Chemother..

[29] Heeb S., Fletcher M.P., Chhabra S.R., Diggle S.P., Williams P., Cámara M. (2011). Quinolones: From antibiotics to autoinducers. FEMS Microbiol. Rev..

[30] Gade N.D., Qazi M.S. (2013). Fluoroquinolone Therapy in Staphylococcus aureus Infections: Where Do We Stand?. J. Lab. Physicians.

[31] Yamaoka T. (2007). The bactericidal effects of anti-MRSA agents with rifampicin and sulfamethoxazole-trimethoprim against intracellular phagocytized MRSA. J. Infect. Chemother..

[32] Elsebaei M.M., Abutaleb N.S., Mahgoub A.A., Li D., Hagras M., Mohammad H., Seleem M.N., Mayhoub A.S. (2019). Phenylthiazoles with nitrogenous side chain: An approach to overcome molecular obesity. Eur. J. Med. Chem..

[33] Hosny Y., Abutaleb N.S., Omara M., Alhashimi M., Elsebaei M.M., Elzahabi H.S., Seleem M.N., Mayhoub A.S. (2020). Modifying the lipophilic part of phenylthiazole antibiotics to control their drug-likeness. Eur. J. Med. Chem..

[34] Elsebaei M.M., Mohammad H., Samir A., Abutaleb N.S., Norvil A.B., Michie A.R., Moustafa M.M., Samy H., Gowher H., Seleem M.N. (2019). Lipophilic efficient phenylthiazoles with potent undecaprenyl pyrophosphatase inhibitory activity. Eur. J. Med. Chem..

[35] Joshi S., Mumtaz S., Singh J., Pasha S., Mukhopadhyay K. (2018). Novel Miniature Membrane Active Lipopeptidomimetics against Planktonic and Biofilm Embedded Methicillin-Resistant Staphylococcus aureus. Sci. Rep..

[36] Garmory H.S., Titball R.W. (2004). ATP-binding cassette transporters are targets for the development of antibacterial vaccines and therapies. Infect. Immun..

[37] Gründling A. (2013). Potassium Uptake Systems in *Staphylococcus aureus*: New Stories about Ancient Systems. mBio.

[38] Vermassen A., Leroy S., Talon R., Provot C., Popowska M., Desvaux M. (2019). Cell Wall Hydrolases in Bacteria: Insight on the Diversity of Cell Wall Amidases, Glycosidases and Peptidases Toward Peptidoglycan. Front. Microbiol..

[39] Connelly J.C., Kirkham L.A., Leach D.R.F. (1998). The SbcCD nuclease of *Escherichia coli* is a structural maintenance of chromosomes (SMC) family protein that cleaves hairpin DNA. Proc. Nat. Acad. Sci. USA.

[40] Fowler R.G., Schaaper R.M. (1997). The role of the mutT gene of Escherichia coli in maintaining replication fidelity. FEMS Microbiol. Rev..

[41] Ho H.N., van Oijen A.M., Ghodke H. (2018). The transcription-repair coupling factor Mfd associates with RNA polymerase in the absence of exogenous damage. Nat. Commun..

[42] Deaconescu A.M., Savery N., Darst S.A. (2007). The bacterial transcription repair coupling factor. Curr. Opin. Struct. Biol..

[43] Gregersen L.H., Svejstrup J.Q. (2018). The Cellular Response to Transcription-Blocking DNA Damage. Trends Biochem. Sci..

[44] Elmwall J., Kwiecinski J., Na M., Ali A.A., Osla V., Shaw L.N., Wang W., Sävman K., Josefsson E., Bylund J. (2017). Galectin-3 Is a Target for Proteases Involved in the Virulence of *Staphylococcus aureus*. Infect. Immun..

[45] Lee L.Y., Miyamoto Y.J., McIntyre B.W., Höök M., McCrea K.W., McDevitt D., Brown E.L. (2002). The Staphylococcus aureus Map protein is an immunomodulator that interferes with T cell-mediated responses. J. Clin. Investig..

[46] Smagur J., Guzik K., Magiera L., Bzowska M., Gruca M., Thøgersen I.B., Enghild J.J., Potempa J. (2009). A new pathway of staphylococcal pathogenesis: Apoptosis-like death induced by Staphopain B in human neutrophils and monocytes. J. Innate Immun..

[47] Nilsson I.M., Hartford O., Foster T., Tarkowski A. (1999). Alpha-toxin and gamma-toxin jointly promote Staphylococcus aureus virulence in murine septic arthritis. Infect. Immun..

[48] Bouza E., Muñoz P. (2000). Monotherapy versus combination therapy for bacterial infections. Med. Clin. N. Am..

[49] Orhan G., Bayram A., Zer Y., Balci I. (2005). Synergy Tests by E Test and Checkerboard Methods of Antimicrobial Combinations against Brucella melitensis. J. Clin. Microbiol..

[50] (2012). Methods for Dilution Antimicrobial Susceptibility Tests f or Bacteria That Grow Aerobically.

[51] Abutaleb N.S., Seleem M.N. (2020). Repurposing the Antiamoebic Drug Diiodohydroxyquinoline for Treatment of Clostridioides difficile Infections. Antimicrob. Agents Chemother..

[52] Naclerio G.A., Abutaleb N.S., Li D., Seleem M.N., Sintim H.O. (2020). Ultrapotent Inhibitor of Clostridioides difficile Growth, Which Suppresses Recurrence In Vivo. J.Med. Chem..

[53] Abutaleb N.S., Seleem M.N. (2020). Auranofin, at clinically achievable dose, protects mice and prevents recurrence from Clostridioides difficile infection. Sci. Rep..

[54] Abutaleb N.S., Seleem M.N. (2020). Antivirulence activity of auranofin against vancomycin-resistant enterococci: In vitro and in vivo studies. Int. J. Antimicrob. Agents.

[55] Kotb A., Abutaleb N.S., Hagras M., Bayoumi A., Moustafa M.M., Ghiaty A., Seleem M.N., Mayhoub A.S. (2019). tert-Butylphenylthiazoles with an oxadiazole linker: A novel orally bioavailable class of antibiotics exhibiting antibiofilm activity. RSC Advances.

[56] Mancy A., Abutaleb N.S., Elsebaei M.M., Saad A.Y., Kotb A., Ali A.O., Abdel-Aleem J.A., Mohammad H., Seleem M.N., Mayhoub A.S. (2020). Balancing Physicochemical Properties of Phenylthiazole Compounds with Antibacterial Potency by Modifying the Lipophilic Side Chain. ACS Infect. Dis..

[57] Shahin I.G., Abutaleb N.S., Alhashimi M., Kassab A.E., Mohamed K.O., Taher A.T., Seleem M.N., Mayhoub A.S. (2020). Evaluation of N-phenyl-2-aminothiazoles for treatment of multi-drug resistant and intracellular Staphylococcus aureus infections. Eur. J. Med. Chem..

[58] Wu M., Hancock R.E.W. (1999). Interaction of the Cyclic Antimicrobial Cationic Peptide Bactenecin with the Outer and Cytoplasmic Membrane. J. Biol. Chem..

[59] Yasir M., Dutta D., Willcox M.D.P. (2019). Mode of action of the antimicrobial peptide Mel4 is independent of Staphylococcus aureus cell membrane permeability. PLoS ONE.

[60] Li L., Shi Y., Su G., Le G. (2012). Selectivity for and destruction of Salmonella typhimurium via a membrane damage mechanism of a cell-penetrating peptide ppTG20 analogue. Int. J. Antimicrob. Agents.

[61] Naclerio G.A., Abutaleb N.S., Onyedibe K.I., Seleem M.N., Sintim H.O. (2019). Potent trifluoromethoxy, trifluoromethylsulfonyl, trifluoromethylthio and pentafluorosulfanyl containing (1,3,4-oxadiazol-2-yl)benzamides against drug-resistant Gram-positive bacteria. RSC Med. Chem..

[62] Hagras M., Abutaleb N.S., Ali A.O., Abdel-Aleem J.A., Elsebaei M.M., Seleem M.N., Mayhoub A.S. (2018). Naphthylthiazoles: Targeting Multidrug-Resistant and Intracellular Staphylococcus aureus with Biofilm Disruption Activity. ACS Infect. Dis..

[63] Dayal N., Opoku-Temeng C., Mohammad H., Abutaleb N.S., Hernandez D., Onyedibe K.I., Wang M., Zeller M., Seleem M.N., Sintim H.O. (2019). Inhibitors of Intracellular Gram-Positive Bacterial Growth Synthesized via Povarov-Doebner Reactions. ACS Infect. Dis..

[64] Singh M.P., Petersen P.J., Weiss W.J., Janso J.E., Luckman S.W., Lenoy E.B., Bradford P.A., Testa R.T., Greenstein M. (2003). Mannopeptimycins, new cyclic glycopeptide antibiotics produced by Streptomyces hygroscopicus LL-AC98: Antibacterial and mechanistic activities. Antimicrob. Agents Chemother..

[65] Opoku-Temeng C., Naclerio G.A., Mohammad H., Dayal N., Abutaleb N.S., Seleem M.N., Sintim H.O. (2018). N-(1,3,4-oxadiazol-2-yl)benzamide analogs, bacteriostatic agents against methicillin- and vancomycin-resistant bacteria. Eur. J. Med. Chem..

[66] Opoku-Temeng C., Onyedibe K.I., Aryal U.K., Sintim H.O. (2019). Proteomic analysis of bacterial response to a 4-hydroxybenzylidene indolinone compound, which re-sensitizes bacteria to traditional antibiotics. J. Proteom..

[67] Eldesouky H.E., Li X., Abutaleb N.S., Mohammad H., Seleem M.N. (2018). Synergistic interactions of sulfamethoxazole and azole antifungal drugs against emerging multidrug-resistant Candida auris. Int. J. Antimicrob. Agents.

[68] Mohammad H., Cushman M., Seleem M.N. (2015). Antibacterial Evaluation of Synthetic Thiazole Compounds In Vitro and In Vivo in a Methicillin-Resistant Staphylococcus aureus (MRSA) Skin Infection Mouse Model. PLoS ONE.

[69] Meletiadis J., Pournaras S., Roilides E., Walsh T.J. (2010). Defining Fractional Inhibitory Concentration Index Cutoffs for Additive Interactions Based on Self-Drug Additive Combinations, Monte Carlo Simulation Analysis, and In Vitro-In Vivo Correlation Data for Antifungal Drug Combinations against Aspergillus fumigatus. Antimicrob. Agents Chemother..

[70] Megaw J., Thompson T.P., Lafferty R.A., Gilmore B.F. (2015). Galleria mellonella as a novel in vivo model for assessment of the toxicity of 1-alkyl-3-methylimidazolium chloride ionic liquids. Chemosphere.

[71] Harding C.R., Schroeder G.N., Collins J.W., Frankel G. (2013). Use of Galleria mellonella as a model organism to study Legionella pneumophila infection. J. Vis. Exp..

