# Supplementary material for: Isoquinoline Antimicrobial Agent: Activity against Intracellular Bacteria and Effect on Global Bacterial Proteome
Source: Molecules. 2022 Aug 10;27(16):5085. doi: 10.3390/molecules27165085 (PMC9416421; doi:10.3390/molecules27165085)

molecules

Article

Isoquinoline Antimicrobial Agent: Activity against Intracellular Bacteria and Effect on Global Bacterial Proteome

Caroline W. Karanja [1], Nimishetti Naganna [1,†], Nader S. Abutaleb [2,3], Neetu Dayal [1], Kenneth I. Onyedibe [1,4], Uma Aryal [2,5], Mohamed N. Seleem [2,3] and Herman O. Sintim [1,4,6,*]

[1] Department of Chemistry, Purdue University, 560 Oval Drive, West Lafayette, IN 47907, USA; cwk4002@med.cornell.edu (C.W.K.); naga06041985@gmail.com (N.N.); ndayal@purdue.edu (N.D.); konyedib@purdue.edu (K.I.O.)
[2] Department of Comparative Pathobiology, Purdue University College of Veterinary Medicine, 625 Harrison Street, West Lafayette, IN 47907, USA; nadershawky@vt.edu (N.S.A.); uaryal@purdue.edu (U.A.); seleem@vt.edu (M.N.S.)
[3] Department of Biomedical Sciences and Pathobiology, Virginia-Maryland College of Veterinary Medicine, Virginia Polytechnic Institute and State University, 1410 Prices Fork Rd, Blacksburg, VA 24061, USA
[4] Purdue Institute of Inflammation, Immunology and Infectious Disease, West Lafayette, IN 47907, USA
[5] Purdue Proteomics Facility, Bindley Bioscience Center, Purdue University, West Lafayette, IN 47907, USA
[6] Institute for Drug Discovery, Purdue University, 720 Clinic Drive, West Lafayette, IN 47907, USA
* Correspondence: hsintim@purdue.edu
† Present address: J. Michael Bishop Institute of Cancer Research, Chengdu 610000, China.

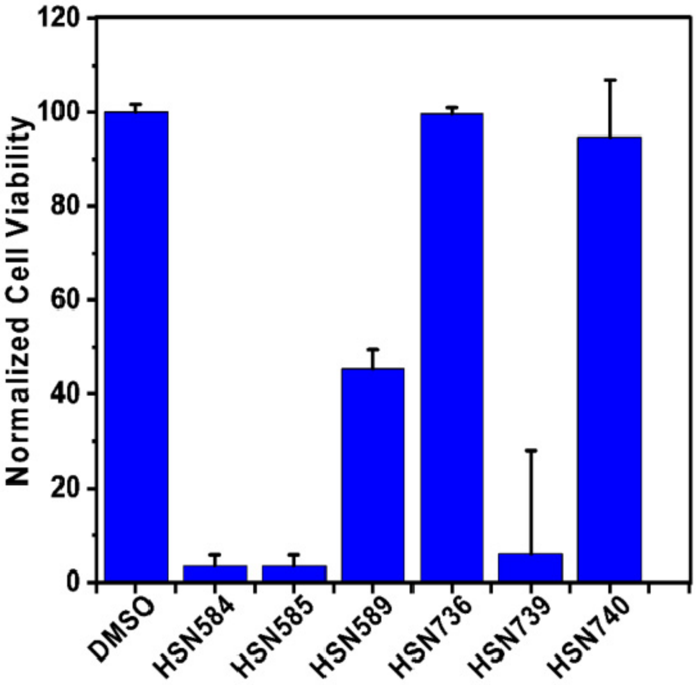

Figure. S1 HSN490 analogs effects on inhibition of bacterial growth. *S. aureus* cell viability after treatment with 16 µg/mL HSN490 analogs

Table S1 Evaluation of alkynyl Isoquinolines against variety of bacterial isolates**.** Minimum inhibitory concentrations (MICs in µg/mL) and minimum bactericidal concentrations (MBCs in µg/mL) of the compounds/control drugs against methicillin-resistant *Staphylococcus aureus* (MRSA), *Staphylococcus epidermidis, Streptococcus pneumoniae, Enterococcus faecium, Enterococcus faecalis, Listeria monocytogenes, Escherichia coli,* and *Clostridium difficile* isolates.

**Bacterial Strains**	**Compounds/Control Drugs**									
	**HSN 490**		**HSN 584**		**HSN 739**		**Linezolid**		**Vancomycin**	
	MIC	MBC	MIC	MBC	MIC	MBC	MIC	MBC	MIC	MBC
**MRSA NRS384 (USA300)**	64	64	4	4	4	4	1	16	1	2
**MRSA NRS119 (Linezolid-resistant)**	32	32	4	4	4	4	64	> 128	2	4
**MRSA NRS123 (USA400)**	64	64	4	4	4	8	1	16	1	2
**Methicillin-resistant *Staphylococcus epidermidis* NRS101**	8	8	4	4	4	4	1	4	1	2

**Cephalosporin-resistant *Streptococcus pneumoniae* ATCC 51916**	4	8	4	4	4	8	1	16	1	2
**Methicillin-resistant *Streptococcus pneumoniae* ATCC 700677**	8	8	4	4	4	4	0.5	8	1	2
***Enterococcus faecalis* ATCC 51299 (VRE)**	64	> 128	8	8	4	4	1	16	64	> 64
***Enterococcus faecium* ATCC 700221 (VRE)**	16	64	4	4	4	8	1	16	> 64	> 64
***Listeria monocytogenes* ATCC 19111**	8	8	4	4	4	4	1	16	1	1
***Escherichia coli* JW55031 (TolC mutant)[a]**	> 128	> 128	> 128	> 128	> 128	> 128	16	64	NT	NT
***Escherichia coli* BW25113[b]**	> 128	> 128	> 128	> 128	> 128	> 128	> 128	> 128	NT	NT
***Clostridium difficile* ATCC BAA-1870**	64	NT	8	NT	8	NT	NT	NT	1	NT
***Clostridium difficile* ATCC 43255**	32	NT	4	NT	8	NT	NT	NT	0.5	NT
***Clostridium difficile* strain I9**	32	NT	16	NT	8	NT	NT	NT	0.5	NT

[a] *E. coli* strain Δ AcrAB-TolC multidrug efflux pump, [b] Wild-type strain of *E. coli* JW55031. NT= not tested.

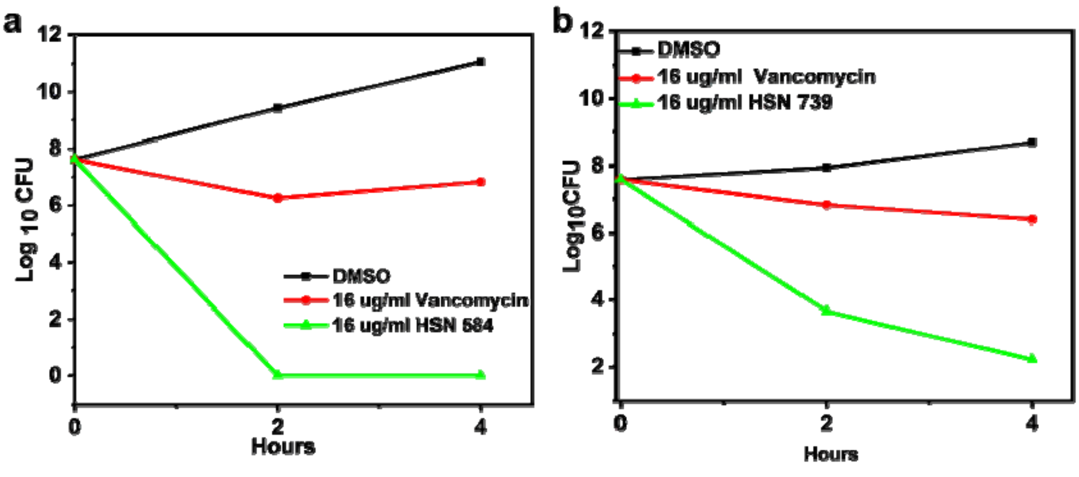

Figure. S2 Time kill profiles. a) HSN584 time kill profile against MRSA USA 300. b) HSN739 time kill profile against MRSA USA 300. Vancomycin was used as a positive control and DMSO (compounds' solvent) as a negative control.

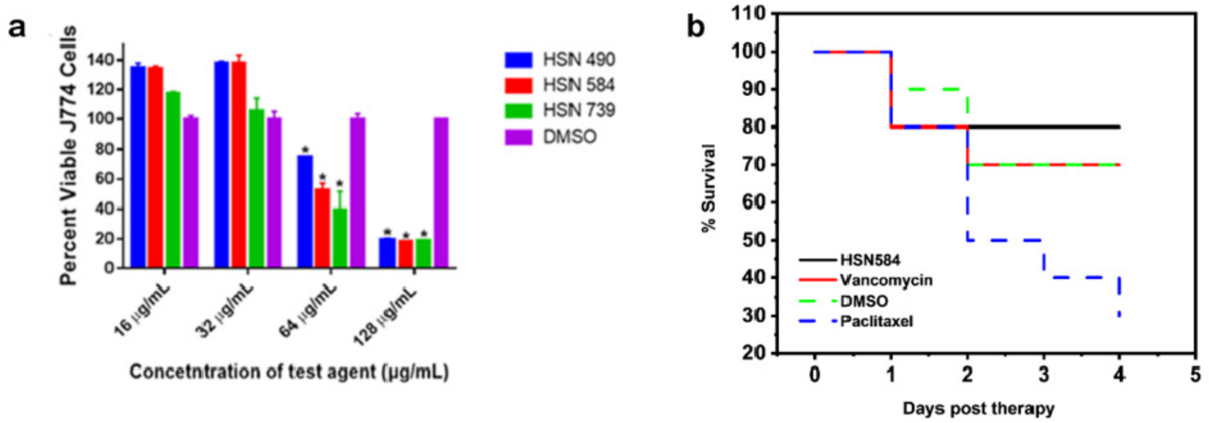

Figure. S3 Alkynyl Isoquinolines cytotoxicity and *in vivo* toxicity. a) HSN490, HSN584 and HSN739 cyto-toxicity against murine macrophages (J774 cell line). b) *Galleria mellonella* survival curves for HSN584. Galleria worms dosed at 10mg/Kg with HSN584 and other compounds, survival monitored over 4 days.

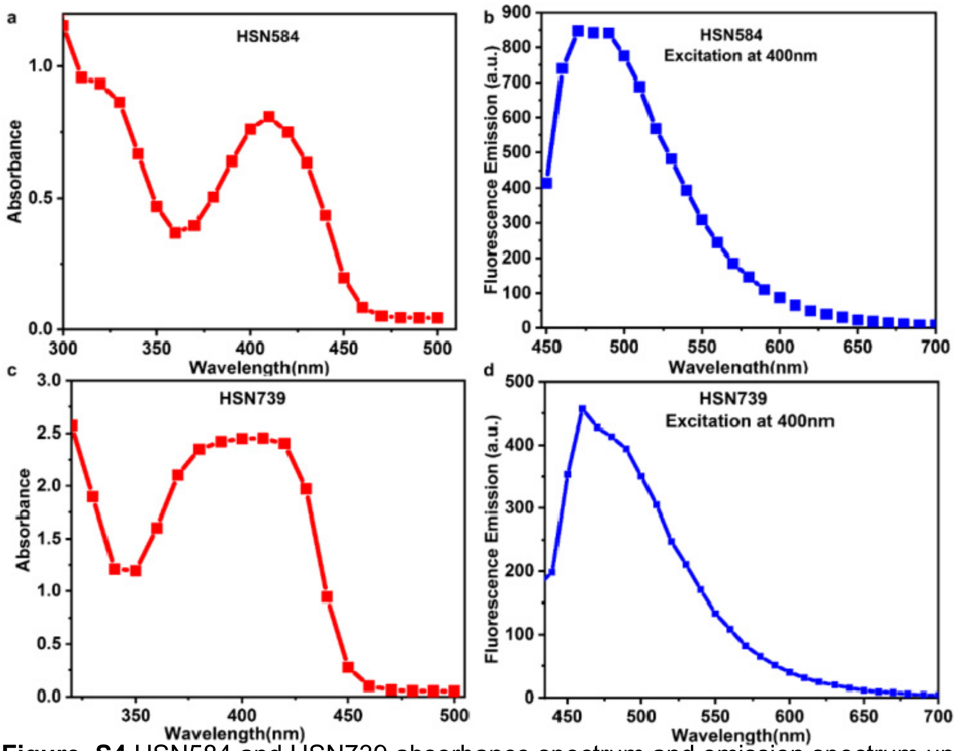

Figure. S4 HSN584 and HSN739 absorbance spectrum and emission spectrum upon excitation at 400 nm

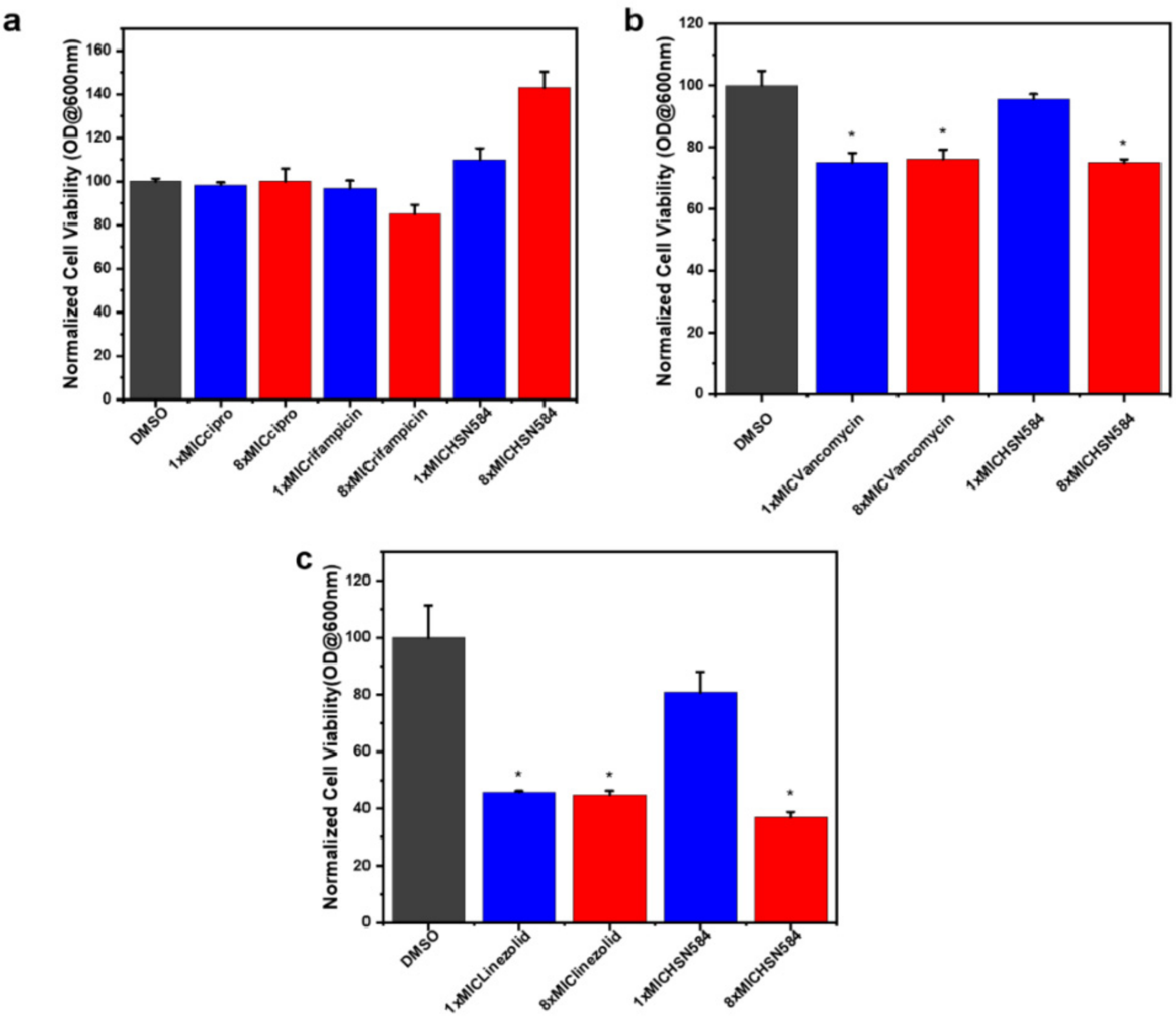

Figure. S5 Positive controls and HSN584 effects on *S. aureus* ATCC 25923 cell viability. a) Cells treated for 20 minutes with respective antibacterial agents. (Cipro = ciproflaxicin) MIC Cipro = 0.125 µg/mL, MIC rifampicin = 7.8 ng/mL, MIC HSN584 = 2 µg/mL. b) Cells were treated for 1 hour with respective antibacterial agents. MIC vancomycin = 2 µg/mL. c) Cells were treated for 1.5 hours with respective antibacterial agents. MIC linezolid = 2 µg/mL. Experiment was done in triplicate. The data were analyzed via the Student's t-test ($P<0.05$). Asterisks (*) denote statistical significant difference of the corresponding treatments as compared to the DMSO (negative control) treatment.

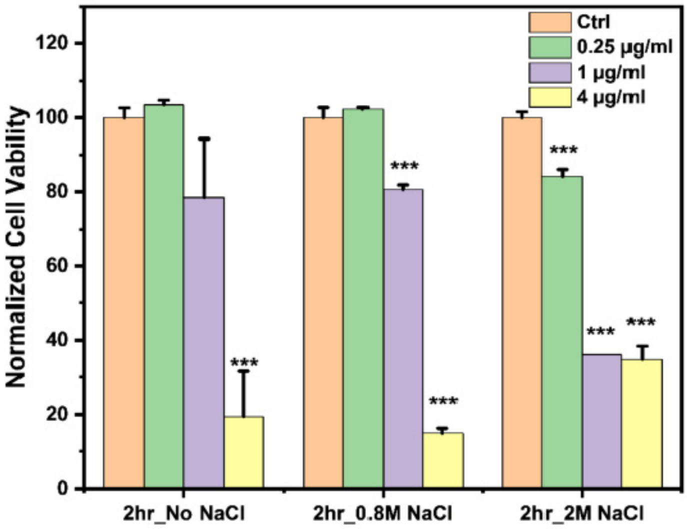

Figure. S6. HSN584 effects on S. aureus ATCC 25923 growth in presence of different concentrations of NaCl. Student's t-test was used for statistical analysis. * p-value < 0.05, ** p- value < 0.01, ***p-value < 0.001.

Table S2 Individual and combination MICs of several commercial antibacterial agents and HSN584 against MRSA ATTC.

Compound	individual MIC (µg/mL)	Combination MIC (µg/mL)	HSN584 individual MIC (µg/mL)	Combination MIC (µg/mL)	εFIC
Methicilllin	128	64	4	2	1
Fosfomycin	128	0.5	4	4	1.003
ceftriaxone	128	32	4	2	0.75
Carbenicillin	>128	64	4	4	1.5
Doxycycline	8	4	4	2	1
Gentamicin	<0.5	0.5	4	4	>2
Bacitracin	128	64	4	2	1
Ampicillin	128	0.5	4	4	1.003
Chloramphenicol	128	32	4	2	0.75
Linezolid	<0.5	0.5	4	0.5	>1.125
Trimethoprim	>128	4	4	1	<0.28
Amikacin	1	0.5	4	2	1
Erythromycin	128	0.5	4	4	1.003
D-cycloserine	32	32	4	2	1.5
Norfloxacin	<0.5	0.5	4	0.5	>1.125
Cloxacin	8	8	4	1	1.25
Clindamycin	<0.5	0.5	4	0.5	>1.125
Kanamycin	>128	128	4	2	<1.5
Danofloxacin	<0.5	0.5	4	0.5	>1.125

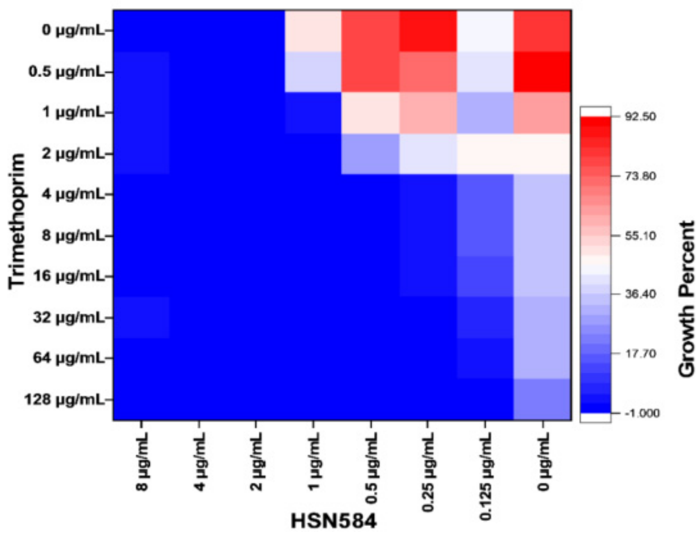

Figure. S7 HSN584 synergizes with Trimethoprim against methicillin-resistant *S. aureus* ATCC 33592.

Chemistry

Unless stated otherwise, all the reagents and solvents were purchased from commercially available suppliers and used without further purification. All the reactions were performed under the blanket of argon. Analytical thin layer chromatography (TLC) was performed on MERCK precoated silica gel 60 F254TLC plates. Compounds were visualized using UV light, Combi Flash was used for the purification of compounds. All NMR spectra were recorded on Bruker 500 MHs spectrometers using Methanol-d_4 or DMSO-d_6 as solvent. The NMR spectra were referenced using residual solvent peaks as the standard. Chemical shifts are denoted in parts per million (δ) and coupling constants (J) are reported in Hertz (Hz). The spin multiplicities are reported as singlet (s), broad singlet (bs), doublet (d), triplet (t), quartet (q), quintet (quint), apparent quintet (app. quint.) and multiplet (m).

General Procedure for the Synthesis of Compounds 1-15:

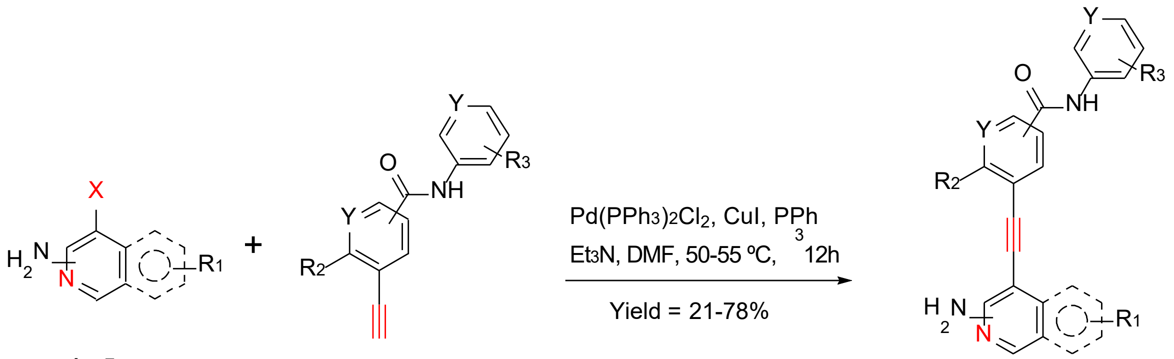

1a-5a
X = Br, I

1b-8b
R2 = H, Me: Y = C, N

1-15

A solution of halo compound **1a-5a**, Pd $(PPh_3)_2Cl_2$ (10 mol%), CuI (5 mol%) and Triphenylphosphine in Triethylamine was de-oxygenated using steam of Argon gas. A de-oxygenated solution of alkyne **1b-8b** in DMF was added slowly over a period of 10 min to the solution and the reaction temperature was increased to (50 – 55) °C and allowed to stir 12 h. The reaction was diluted with ethyl acetate. The organic layer was washed with water, saturated NH_4Cl, and washed with brine. Combined organic layers were dried over anhydrous sodium sulphate, filtered and concentrated in vacuo. The pure product was obtained by flash column chromatography.

Synthesis and Analytical Data of Compounds

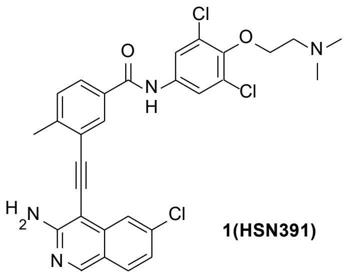

1(HSN391)

Compound 1: 3-((3-Amino-6-chloroisoquinolin-4-yl) ethynyl)-N-(3,5-dichloro-4-(2-(dimethylamino) ethoxy) phenyl)-4-methylbenzamide: A solution of Iodo compound **1a** (50 mg, 0.17 mmol, 1 equiv), Pd $(PPh_3)_2Cl_2$ (10 mol%), CuI (5 mol%) and Triphenylphosphine (5 mg) in Triethylamine (1 mL) was de-oxygenated using steam of Argon gas. A de-oxygenated solution of alkyne **1b** (77 mg, 0.2 mmol, 1.2 equiv) in DMF (3 mL) was added slowly over a period of 10 min to the solution and the reaction temperature was increased to 55 °C and allowed to stir 12 h. The reaction was diluted with ethyl acetate (200 mL). The organic layer was washed with water (5 × 50 mL), saturated NH_4Cl (1 × 50 mL), and washed with brine (1 × 50 mL). Combined organic layers were dried over anhydrous sodium sulphate, filtered and concentrated in vacuo. The pure product **HSN391** was obtained by flash column chromatography. **Yield** = 35%.; **TLC R*f*** = 0.2 (10 % $MeOH/CH_2Cl_2$); **^{1}H NMR** (500 MHz, DMSO-d_6) δ 10.47 (s, 1H), 8.92 (s, 1H), 8.34 (d, *J* = 1.9 Hz, 1H), 7.96 (d, *J* = 8.7 Hz, 1H), 7.94 (s, 2H), 7.88 (d, *J* = 1.9 Hz, 1H), 7.84 (dd, *J* = 8.0, 2.0 Hz, 1H), 7.52 (d, *J* = 8.1 Hz, 1H), 7.28 (dd, *J* = 8.6, 2.0 Hz, 1H), 6.79 (s, 2H), 4.02 (t, *J* = 5.9 Hz, 2H), 2.68 (t, *J* = 5.9 Hz, 2H), 2.63 (s, 3H), 2.23 (s, 6H); **^{13}C NMR** (126 MHz, DMSO-d_6) δ 165.5, 158.5, 153.2, 147.0, 146.9, 143.45, 138.7, 137.4, 136.7, 132.6, 131.6, 131.4, 130.2, 128.6, 128.5, 128.0, 123.5, 121.3, 120.7, 113.7, 98.0, 88.7, 72.0, 58.7, 45.9, 21.2; **HRMS (ESI+):** calcd. for $C_{29}H_{27}Cl_3N_4O_2$ (MH^+) 567.1122, found 567.1118.

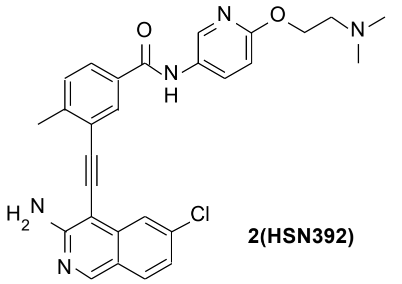

2(HSN392)

Compound 2: 3-((3-Amino-6-chloroisoquinolin-4-yl) ethynyl)-N-(6-(2-(dimethylamino) ethoxy) pyridin-3-yl)-4-methylbenzamide: A solution of Iodo compound **1a** (50 mg, 0.17 mmol, 1 equiv), Pd $(PPh_3)_2Cl_2$ (10 mol%), CuI (5 mol%) and Triphenylphosphine (5 mg) in Triethylamine (1 mL) was de-oxygenated using steam of Argon gas. A de-oxygenated solution of alkyne **2b** (65 mg, 0.2 mmol, 1.2 equiv) in DMF (3 mL) was added slowly over a period of 10 min to the solution and the reaction temperature was increased to 55 °C and allowed to stir 12 h. The reaction was diluted with ethyl acetate (200 mL). The organic layer was washed with water (5 × 50 mL), saturated NH_4Cl (1 × 50 mL), and washed with brine (1 × 50 mL). Combined organic layers were dried over anhydrous sodium sulphate, filtered and concentrated in vacuo. The pure product **HSN392** was obtained by flash column chromatography. **Yield** = 40%.; **TLC R*f*** = 0.1 (10 % $MeOH/CH_2Cl_2$); **^{1}H NMR** (500 MHz, DMSO-d_6) δ 10.32 (s, 1H), 8.92 (s, 1H), 8.50 (d, *J* = 2.7 Hz, 1H), 8.36 (d, *J* = 1.8 Hz, 1H), 8.03 (dd, *J* = 8.9, 2.7 Hz, 1H), 7.96 (d, *J* = 8.6 Hz, 1H), 7.89 (s, 1H), 7.86 (d, *J* = 7.9 Hz, 1H), 7.50 (d, *J* = 8.0 Hz, 1H), 7.28 (dd, *J* = 8.6, 2.0 Hz, 1H),

6.82 (d, *J* = 8.9 Hz, 1H), 6.79 (s, 2H), 4.30 (t, *J* = 5.9 Hz, 2H), 2.63 (s, 3H), 2.59 (t, *J* = 5.9 Hz, 2H), 2.19 (s, 6H); **$_{13}$C NMR** (126 MHz, DMSO-*d*$_6$) δ 165.2, 159.9, 158.5, 153.2, 143.0, 139.3, 138.7, 137.3, 133.0, 132.8, 131.6, 131.4, 130.3, 130.1, 127.9, 123.5, 121.3, 120.6, 110.7, 98.2, 88.8, 88.5, 63.9, 58.1, 45.9, 21.2; **HRMS (ESI+):** calcd. for $C_{28}H_{27}ClN_5O_2$ (MH+) 500.1853, found 500.1856.

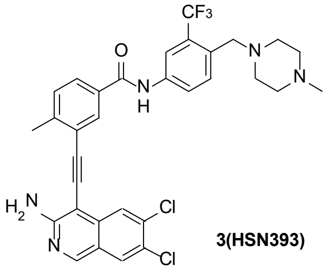

3(HSN393)

Compound 3: 3-((3-Amino-6,7-dichloroisoquinolin-4-yl) ethynyl)-4-methyl-N-(4-((4-methylpiperazin-1-yl) methyl)-3-(trifluoromethyl) phenyl) benzamide: A solution of Iodo compound **1a** (30 mg, 0.09 mmol, 1 equiv), Pd $(PPh_3)_2Cl_2$ (10 mol%), CuI (5 mol%) and Triphenylphosphine (5 mg) in Triethylamine (0.5 mL) was de-oxygenated using steam of Argon gas. A de-oxygenated solution of alkyne **3b** (55 mg, 0.13 mmol, 1.5 equiv) in DMF (1.5 mL) was added slowly over a period of 10 min to the solution and the reaction temperature was increased to 55 ºC and allowed to stir 12 h. The reaction was diluted with ethyl acetate (150 mL). The organic layer was washed with water (5 × 30 mL), saturated NH_4Cl (1 × 30 mL), and washed with brine (1 × 50 mL). Combined organic layers were dried over anhydrous sodium sulphate, filtered and concentrated in vacuo. The pure product **HSN393** was obtained by flash column chromatography. **Yield** = 68%.; **TLC R*f*** = 0.2 (10% MeOH/CH_2Cl_2); **$_1$H NMR** (500 MHz, DMSO-*d*$_6$) δ 10.53 (s, 1H), 8.91 (s, 1H), 8.38 (d, *J* = 1.9 Hz, 1H), 8.27 (s, 1H), 8.20 (d, *J* = 2.2 Hz, 1H), 8.05 (d, *J* = 7.1 Hz, 2H), 7.88 (dd, *J* = 7.9, 2.0 Hz, 1H), 7.70 (d, *J* = 8.5 Hz, 1H), 7.51 (d, *J* = 8.1 Hz, 1H), 6.91 (s, 2H), 3.55 (s, 2H), 2.63 (s, 3H), 2.37 (s, 8H), 2.15 (s, 3H); **$_{13}$C NMR** (126 MHz, DMSO-*d*$_6$) $_{13}$C NMR (126 MHz, DMSO-*d*$_6$) δ 165.5, 158.6, 152.4, 143.3, 138.7, 137.0, 135.4, 132.8, 132.5, 131.7, 131.6, 130.6, 130.2, 128.1, 128.0 (q, *J* = 30.2 Hz), 125.9(q, *J* = 274.6 Hz), 124.9, 124.0, 123.9, 123.3, 121.3, 117.6, 98.3, 88.4, 57.9, 55.1, 53.1, 46.1, 21.2; **HRMS (ESI+):** calcd. for $C_{32}H_{29}Cl_2F_3N_5O$ (MH+) 626.1701, found 626.1698.

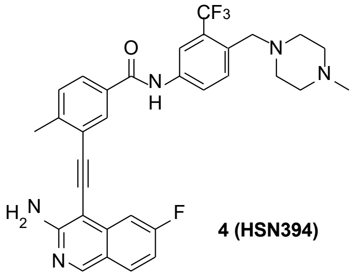

4 (HSN394)

Compound 4: 3-((3-Amino-6-fluoroisoquinolin-4-yl)ethynyl)-4-methyl-N-(4-((4-methylpiperazin-1-yl)methyl)-3-(trifluoromethyl)phenyl)benzamide: A solution of Iodo compound **2a** (30 mg, 0.1 mmol, 1 equiv), Pd$(PPh_3)_2$ Cl_2 (10 mol%), CuI (5 mol%) and triphenylphosphine (4 mg) in Triethylamine (0.5 mL, 3 mmol, 30 equiv) was de-oxygenated using steam of Argon gas. A de-oxygenated solution of alkyne **3b** (65mg, 0.16 mmol, 1.5 equiv) in DMF (2 mL) was added slowly over a period of 10 min to the solution and the reaction temperature was increased to 50 ºC and allowed to stir 12 h. The reaction was quenched by addition of NH_4Cl (2 mL) at room temperature and diluted with ethyl acetate (100 mL). The organic layer was washed with water (5 × 30 mL) and washed with brine (20 mL). Combined organic layers were dried over anhydrous sodium sulphate, filtered and concentrated in vacuo. The pure product **4** was obtained by flash column chromatography. **Yield** = 60%; **TLC R*f*** = 0.2 (10% MeOH/CH_2Cl_2); **$_1$H NMR** (500 MHz, Methanol-*d*$_4$) δ 8.93 (s, 1H), 8.37 (d, *J* = 1.9 Hz, 1H), 8.25 (d, *J* = 2.2 Hz, 1H), 8.10 – 8.02 (m, 2H), 7.95 (dd, *J* = 8.0, 2.0 Hz, 1H), 7.81 (d, *J* = 8.4 Hz, 1H), 7.66 (dd, *J* = 10.6, 2.4 Hz, 1H), 7.57 (d, *J* = 8.0 Hz, 1H), 7.19 (td, *J* = 8.8, 2.5 Hz, 1H), 3.69 (s, 2H), 2.74 (s, 3H), 2.56 (s, 8H), 2.35 (s, 3H); $_{13}$C NMR (126 MHz, Methanol-*d*$_4$ + DMSO-*d*$_6$) δ 165.9, 165.9(d, *J* =

250.74 Hz), 157.9, 152.4, 143.4, 139.8, 138.3, 132.7, 132.4, 131.4, 130.9, 129.9, 128.5, 127.7, 125.7(q, J = 273.4 Hz), 123.7, 123.4, 120.0, 117.6, 113.2, 106.1, 98.0, 88.0, 57.6, 54.7, 52.4, 44.9, 20.4; **HRMS (ESI+):** calcd. for $C_{32}H_{30}F_4N_5O$ (MH^+) 576.2386, found 576.2382.

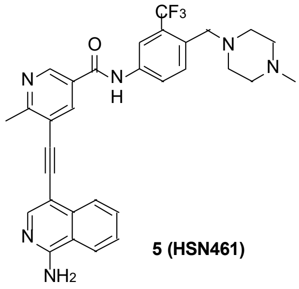

5 (HSN461)

Compound 5: 5-((1-Aminoisoquinolin-4-yl)ethynyl)-6-methyl-N-(4-((4-methylpiperazin-1-yl)methyl)-3-(trifluoromethyl)phenyl)nicotinamide: A solution of Bromo compound **3a** (77 mg, 0.35 mmol, 1.2 equiv), $Pd(PPh_3)_2$ Cl_2 (10 mol%), CuI (5 mol%) and Triphenylphosphine (10 mg) in Triethylamine (1 mL, 7 mmol, 20 equiv) was de-oxygenated using steam of Argon gas. A de-oxygenated solution of alkyne **4b** (120mg, 0.29 mmol, 1 equiv) in DMF (3 mL) was added slowly over a period of 10 min to the solution and the reaction temperature was increased to 50 ºC and allowed to stir 12 h. The reaction was quenched by addition of NH_4Cl (5 mL) at room temperature and diluted with ethyl acetate (300 mL). The organic layer was washed with water (5 × 50 mL) and washed with brine (50 mL). Combined organic layers were dried over anhydrous sodium sulphate, filtered and concentrated in vacuo. The pure product **5** was obtained by flash column chromatography. **Yield** = 31%.; **TLC R$_f$**= 0.2 (10% MeOH/CH_2Cl_2); **^{1}H NMR** (500 MHz, DMSO-d_6) δ 10.66 (s, 1H), 8.93 (d, J = 2.3 Hz, 1H), 8.48 (d, J = 2.3 Hz, 1H), 8.29 (d, J = 8.1 Hz, 1H), 8.22 (s, 1H), 8.20 (d, J = 2.2 Hz, 1H), 8.11 (d, J = 8.1 Hz, 1H), 8.04 (dd, J = 8.4, 2.2 Hz, 1H), 7.81 (ddd, J = 8.1, 6.9, 1.2 Hz, 1H), 7.72 (d, J = 8.5 Hz, 1H), 7.58 (ddd, J = 8.3, 6.9, 1.3 Hz, 1H), 7.45 (s , 2H), 3.56 (s, 2H), 2.81 (s, 3H), 2.37 (s, 8H), 2.14 (s, 3H); **^{13}C NMR** ^{13}C NMR (126 MHz, DMSO-d_6) δ 164.1, 162.2, 158.4, 148.1, 147.2, 138.3, 137.6, 136.3, 132.8, 131.7, 131.7, 128.0 (q, J = 30.2 Hz), 127.9, 126.8, 125.8(q, J = 274.6 Hz), 125.0, 124.7, 124.0, 119.1, 117.7, 116.7, 102.6, 94.2, 89.9, 57.9, 55.2, 53.1, 46.2, 24.3; **HRMS (ESI+):** calcd. for $C_{31}H_{30}F_3N_6O$ (MH^+) 559.2433, found 559.2430.

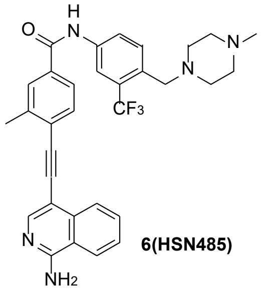

6(HSN485)

Compound 6: 4-((1-Aminoisoquinolin-4-yl) ethynyl)-3-methyl-N-(4-((4-methylpiperazin-1-yl) methyl)-3-(trifluoromethyl) phenyl) benzamide: A solution of Bromo compound **3a** (59 mg, 0.26 mmol, 1.2 equiv), Pd $(PPh_3)_2Cl_2$ (10 mol%), CuI (5 mol%) and Triphenylphosphine (5 mg) in Triethylamine (1 mL) was de-oxygenated using steam of Argon gas. A de-oxygenated solution of alkyne **5b** (90 mg, 0.22 mmol, 1 equiv) in DMF (3 mL) was added slowly over a period of 10 min to the solution and the reaction temperature was increased to 55 ºC and allowed to stir 12 h. The reaction was diluted with ethyl acetate (200 mL). The organic layer was washed with water (5 × 30 mL), saturated NH_4Cl (1 × 30 mL), and washed with brine (1 × 30 mL). Combined organic layers were dried over anhydrous sodium sulphate, filtered and concentrated in vacuo. The pure product **HSN485** was obtained by flash column chromatography. **Yield** = 21%.; **TLC R$_f$** = 0.2 (10% MeOH/CH_2Cl_2); **^{1}H NMR** (500 MHz, Methanol-d_4) δ 8.18 (t, J = 8.7 Hz, 2H), 8.11 (d, J = 21.9 Hz, 2H), 7.93 (d, J = 8.5 Hz, 1H), 7.88 (s, 1H), 7.80 (t, J = 8.4 Hz, 2H), 7.75 (d, J = 8.5 Hz, 1H), 7.65 (d, J = 8.0 Hz, 1H), 7.59 (t, J = 7.8 Hz, 1H), 3.64 (s, 2H), 2.66 (s, 3H), 2.51 (s, 8H), 2.29 (s, 3H);^{13}C NMR (126 MHz, Methanol-d_4) δ 166.8, 157.6, 145.3, 139.5, 137.8, 136.4, 133.4, 132.6, 131.2, 131.1, 128.8 (q, J = 30.2 Hz), 128.3, 127.2, 126.5, 125.4 (q, J = 273.4 Hz), 124.7, 124.7, 123.7, 123.5, 117.7, 116.9, 104.8,

92.1, 91.3, 57.5, 54.5, 52.3, 44.5, 19.8; **HRMS (ESI+):** calcd. for $C_{32}H_{31}F_3N_5O$ (MH+) 558.2481, found 558.2477.

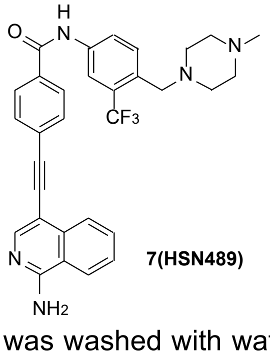

7(HSN489)

Compound 7: 4-((1-Aminoisoquinolin-4-yl) ethynyl)-N-(4-((4-methylpiperazin-1-yl) methyl)-3-(trifluoromethyl) phenyl) benzamide: A solution of Bromo compound **3a** (98 mg, 0.44 mmol, 2 equiv), Pd $(PPh_3)_2Cl_2$ (10 mol%), CuI (5 mol%) and Triphenylphosphine (5 mg) in Triethylamine (1 mL) was de-oxygenated using steam of Argon gas. A de-oxygenated solution of alkyne **6b** (90 mg, 0.22 mmol, 1 equiv) in DMF (3 mL) was added slowly over a period of 10 min to the solution and the reaction temperature was increased to 55 °C and allowed to stir 12 h. The reaction was diluted with ethyl acetate (200 mL). The organic layer was washed with water (5 × 30 mL), saturated NH_4Cl (1 × 30 mL), and washed with brine (1 × 30 mL). Combined organic layers were dried over anhydrous sodium sulphate, filtered and concentrated in vacuo. The pure product **HSN489** was obtained by flash column chromatography. **Yield** = 27%.; **TLC R*f*** = 0.2 (10% MeOH/CH_2Cl_2); **^{1}H NMR** (500 MHz, Methanol-*d*4) δ 8.18 (t, *J* = 9.3 Hz, 2H), 8.13 (d, *J* = 2.3 Hz, 1H), 8.08 (s, 1H), 7.98 (d, *J* = 8.6 Hz, 2H), 7.94 (dd, *J* = 8.4, 2.3 Hz, 1H), 7.81 (ddd, *J* = 8.2, 6.9, 1.2 Hz, 1H), 7.76 (d, *J* = 8.5 Hz, 1H), 7.71 (d, *J* = 8.6 Hz, 2H), 7.60 (ddd, *J* = 8.3, 7.0, 1.3 Hz, 1H), 3.65 (s, 2H), 2.52 (s, 8H), 2.30 (s, 3H); ^{13}C NMR (126 MHz, Methanol-*d*4) δ 166.6, 157.6, 145., 137., 136.5, 133.4, 132.6, 131.2, 131.1, 130.8, 128.8 (q, *J* = 30.2 Hz), 127.5, 127.4, 126.5, 125.4 (q, *J* = 273.4 Hz), 124.7, 123.7, 123.6, 117.7, 116.9, 104.5, 92.4, 88.0, 57.5, 54.5, 52.2, 48.1, 44.5; **HRMS (ESI+):** calcd. for $C_{31}H_{29}F_3N_5O$ (MH+) 544.2324, found 544.2319.

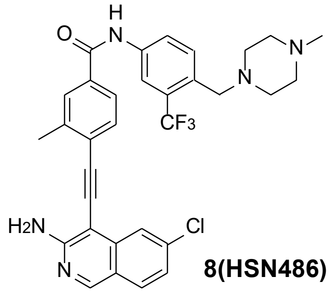

8(HSN486)

Compound 8: 4-((3-Amino-6-chloroisoquinolin-4-yl) ethynyl)-3-methyl-N-(4-((4-methylpiperazin-1-yl) methyl)-3-(trifluoromethyl) phenyl) benzamide: A solution of Iodo compound **1a** (55 mg, 0.18 mmol, 1 equiv), Pd $(PPh_3)_2Cl_2$ (10 mol%), CuI (5 mol%) and Triphenylphosphine (5 mg) in Triethylamine (1 mL) was de-oxygenated using steam of Argon gas. A de-oxygenated solution of alkyne **5b** (90 mg, 0.22 mmol, 1.2 equiv) in DMF (3 mL) was added slowly over a period of 10 min to the solution and the reaction temperature was increased to 55°C and allowed to stir 12 h. The reaction was diluted with ethyl acetate (200 mL). The organic layer was washed with water (5 × 30 mL), saturated NH_4Cl (1 × 30 mL), and washed with brine (1 × 30 mL). Combined organic layers were dried over anhydrous sodium sulphate, filtered and concentrated in vacuo. The pure product **HSN486** was obtained by flash column chromatography. **Yield** = 22%.; **TLC R*f*** = 0.2 (10% MeOH/CH_2Cl_2); **^{1}H NMR** (500 MHz, Methanol-*d*4) δ 8.79 (s, 1H), 8.14 (s, 1H), 7.95 (m, 2H), 7.90 (s, 1H), 7.85 (d, *J* = 8.7 Hz, 1H), 7.81 (dd, *J* = 8.0, 1.8 Hz, 1H), 7.76 (m, 2H), 7.25 (dd, *J* = 8.7, 2.0 Hz, 1H), 3.67 (s, 2H), 2.69 (s, 3H), 2.61 (bs, 8H), 2.40 (s, 3H);^{13}C NMR (126 MHz, Methanol-*d*4) δ 166.7, 157.4, 151.9, 139.4, 138.7, 138.1, 137.9, 133.8, 132.4, 131.5, 131.2, 130.4, 128.6, 128.4, 126.6, 125.4(q, *J* = 274.6 Hz), 124.8, 124.5, 123.7, 123.6, 121.3, 120.8, 117.7, 98.2, 90.5, 88.9, 57.4, 54.4, 51.8, 44.1, 20.0; **HRMS (ESI+):** calcd. for $C_{32}H_{30}ClF_3N_5O$ (MH+) 592.2091, found 592.2085.

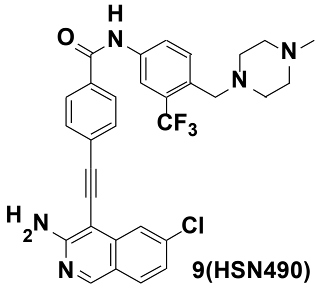

9(HSN490)

Compound 9: 4-((3-Amino-6-chloroisoquinolin-4-yl) ethynyl)-N-(4-((4-methylpiperazin-1-yl) methyl)-3-(trifluoromethyl) phenyl) benzamide: A solution of Iodo compound **1a** (100 mg, 0.33 mmol, 1.5 equiv), Pd $(PPh_3)_2Cl_2$ (10 mol%), CuI (5 mol%) and Triphenylphosphine (5 mg) in Triethylamine (1 mL) was de-oxygenated using steam of Argon gas. A de-oxygenated solution of alkyne **6b** (90 mg, 0.22 mmol, 1 equiv) in DMF (3 mL) was added slowly over a period of 10 min to the solution and the reaction temperature was increased to 55 ºC and allowed to stir 12 h. The reaction was diluted with ethyl acetate (200 mL). The organic layer was washed with water (5 × 30 mL), saturated NH_4Cl (1 × 30 mL), and washed with brine (1 × 30 mL). Combined organic layers were dried over anhydrous sodium sulphate, filtered and concentrated in vacuo. The pure product **HSN490** was obtained by flash column chromatography. **Yield** = 41%.; **TLC** $\mathbf{R}_f$ = 0.4 (10% MeOH/CH_2Cl_2); ^{1}**H NMR** (500 MHz, DMSO-d_6) δ 10.56 (s, 1H), 8.92 (s, 1H), 8.21 (s, 1H), 8.06 (s, 1H), 8.04 (d, *J* = 8.3 Hz, 2H), 7.99 – 7.91 (m, 3H), 7.89 (s, 1H), 7.70 (d, *J* = 8.5 Hz, 1H), 7.28 (dd, *J* = 8.6, 2.0 Hz, 1H), 6.89 (s, 2H), 3.56 (s, 2H), 2.38 (s, 8H), 2.17 (s, 3H); ^{13}C NMR (126 MHz, DMSO-d_6) δ 165.5, 158.8, 153.5, 138.7, 138.6, 137.4, 133.9, 132.5, 131.7, 131.6, 128.3, 128.04 (q, *J* = 30.2 Hz), 126.8, 125.94 (q, *J* = 274.6 Hz), 124.0, 123.5, 121.3, 120.6, 117.7, 99.5, 88.2, 86.5, 57.8, 55.1, 53.0, 46.0.; **HRMS (ESI+):** calcd. for $C_{31}H_{28}ClF_3N_5O$ (MH^+) 578.1934, found 578.1930.

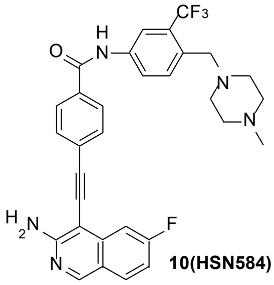

10(HSN584)

Compound 10: 4-((3-Amino-6-fluoroisoquinolin-4-yl) ethynyl)-N-(4-((4-methylpiperazin-1-yl) methyl)-3-(trifluoromethyl) phenyl) benzamide: A solution of Iodo compound **2a** (98 mg, 0.34 mmol, 1 equiv), Pd $(PPh_3)_2Cl_2$ (10 mol%), CuI (5 mol%) and Triphenylphosphine (5 mg) in Triethylamine (1.2 mL) was de-oxygenated using steam of Argon gas. A de-oxygenated solution of alkyne **6b** (150 mg, 0.4 mmol, 1.2 equiv) in DMF (3 mL) was added slowly over a period of 10 min to the solution and the reaction temperature was increased to 55 ºC and allowed to stir 12 h. The reaction was diluted with ethyl acetate (300 mL). The organic layer was washed with water (5 × 30 mL), saturated NH_4Cl (1 × 30 mL), and washed with brine (1 × 30 mL). Combined organic layers were dried over anhydrous sodium sulphate, filtered and concentrated in vacuo. The pure product **HSN584** was obtained by flash column chromatography. **Yield** = 38%.; **TLC** $\mathbf{R}_f$ = 0.5 (10% MeOH/CH_2Cl_2); ^{1}**H NMR** (500 MHz, DMSO-d_6) δ 10.55 (s, 1H), 8.89 (s, 1H), 8.21 (d, *J* = 2.2 Hz, 1H), 8.08 – 7.97 (m, 4H), 7.97 – 7.92 (m, 2H), 7.70 (d, *J* = 8.5 Hz, 1H), 7.58 (dd, *J* = 10.8, 2.5 Hz, 1H), 7.15 (td, *J* = 8.8, 2.5 Hz, 1H), 6.84 (s, 2H), 3.56 (s, 2H), 2.38 (s, 8H), 2.17 (s, 3H); ^{13}C NMR (126 MHz, DMSO-d_6) δ 165.7(d, *J* = 249.4 Hz), 165.5, 158.6, 153.4, 139.8, 139.7, 138.8, 133.7, 132.9, 131.8, 131.6, 128.3, 128.1(q, *J* = 30.2 Hz), 127.0, 125.8(q, *J* = 274.6 Hz), 124.0, 119.7, 117.8, 113.2, 106.3, 99.5, 88.9, 86.8, 57.3, 53.9, 50.9, 43.8; **HRMS (ESI+):** calcd. for $C_{31}H_{28}F_4N_5O$ (MH^+) 562.2224, found 562.2224.

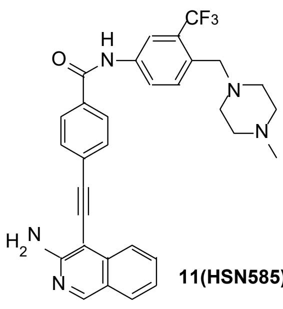

11(HSN585)

Compound 11: 4-((3-Aminoisoquinolin-4-yl) ethynyl)-N-(4-((4-methylpiperazin-1-yl) methyl)-3-(trifluoromethyl) phenyl) benzamide: A solution of iodocompound **4a** (98 mg, 0.44 mmol, 2 equiv), Pd $(PPh_3)_2Cl_2$ (10 mol%), CuI (5 mol%) and Triphenylphosphine (5 mg) in Triethylamine (1 mL) was de-oxygenated using steam of Argon gas. A de-oxygenated solution of alkyne **6b** (90 mg, 0.22 mmol, 1 equiv) in DMF (3 mL) was added slowly over a period of 10 min to the solution and the reaction temperature was increased to 55 ºC and allowed to stir 12 h. The reaction was diluted with ethyl acetate (200 mL). The organic layer was washed with water (5 × 30 mL), saturated NH_4Cl (1 × 30 mL), and washed with brine (1 × 30 mL). Combined organic layers were dried over anhydrous sodium sulphate, filtered and concentrated in vacuo. The pure product **HSN585** was obtained by flash column chromatography. **Yield** = 29%.; **TLC** $\mathbf{R}_f$ = 0.2 (10% MeOH/CH_2Cl_2); ^{1}**H NMR** (500 MHz, DMSO-d_6) δ 10.56 (s, 1H), 8.89 (s, 1H), 8.21 (d, *J* = 2.2 Hz, 1H), 8.04 (d, *J* = 8.5 Hz, 3H), 7.98 (d, *J* = 8.5 Hz, 1H), 7.91 (dd, *J* = 10.5, 8.2 Hz, 3H), 7.72 – 7.63 (m, 3H), 7.31 – 7.24 (m, 1H), 6.68 (s, 2H), 3.56 (s, 2H), 2.38 (s, 8H), 2.17 (s, 3H); ^{13}**C NMR** (126 MHz, DMSO-d_6) δ 165.5, 158.2, 153.4, 138.6, 137.9, 133.7, 132.5, 132.1, 131.7, 131.5, 129.1, 128.3, 128.0 (q, *J* = 30.2 Hz), 127.1, 125.9 (q, *J* = 123.7 Hz), 124.0, 123.1, 122.8, 122.3, 117.7, 99.2, 89.1, 87.4, 57.8, 55.1, 53.0, 46.0, 40.4. **HRMS (ESI+):** calcd. for $C_{31}H_{29}F_3N_5O$ (MH^+) 544.2318, found 544.20

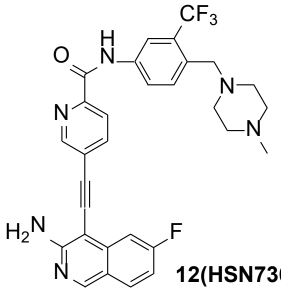

12(HSN736)

Compound 12: 5-((3-Amino-6-fluoroisoquinolin-4-yl) ethynyl)-N-(4-((4-methylpiperazin-1-yl) methyl)-3-(trifluoromethyl) phenyl) picolinamide: A solution of Iodo compound **2a** (85 mg, 0.3 mmol, 1 equiv), Pd $(PPh_3)_4$ (10 mol%), CuI (5 mol%) and Triphenylphosphine (10 mg) in Triethylamine (1 mL) was de-oxygenated using steam of Argon gas. A de-oxygenated solution of alkyne **7b** (142 mg, 0.35 mmol, 1.2 equiv) in DMF (3 mL) was added slowly over a period of 10 min to the solution and the reaction temperature was increased to 55 ºC and allowed to stir 12 h. The reaction was diluted with ethyl acetate (300 mL). The organic layer was washed with water (5 × 50 mL), saturated NH_4Cl (1 × 50 mL) and brine (1 × 50 mL). Combined organic layers were dried over anhydrous sodium sulphate, filtered and concentrated in vacuo. The pure product **HSN736** was obtained by flash column chromatography. **Yield** = 78%; **TLC** $\mathbf{R}_f$= 0.2 (5% MeOH/CH_2Cl_2); ^{1}**H NMR** (500 MHz, DMSO-d_6) δ 10.99 (s, 1H), 9.10 (s, 1H), 8.91 (s, 1H), 8.46 (dd, *J* = 8.1, 2.1 Hz, 1H), 8.39 (d, *J* = 2.3 Hz, 1H), 8.19 (d, *J* = 8.1 Hz, 1H), 8.16 (dd, *J* = 8.4, 2.2 Hz, 1H), 8.02 (dd, *J* = 8.9, 5.9 Hz, 1H), 7.70 (d, *J* = 8.5 Hz, 1H), 7.64 (dd, *J* = 10.7, 2.5 Hz, 1H), 7.16 (td, *J* = 8.8, 2.5 Hz, 1H), 6.99 (s, 2H), 3.56 (s, 2H), 2.38 (s, 8H), 2.14 (s, 3H); ^{13}C NMR (126 MHz, DMSO-d_6) δ 165.8(d, *J* = 250.7 Hz), 163.0, 159.0, 153.9, 150.6, 148.1, 140.4, 139.8, 137.9, 132.8, 131.5, 128.0, 127.3, 125.8 (q, *J* = 273.4 Hz), 124.2, 123.7, 122.5, 119.6, 118.0, 113.2, 106.3, 96.5, 90.2, 88.3, 57.8, 55.1, 53.0, 46.0; **HRMS (ESI+):** calcd. for $C_{30}H_{27}F_4N_6O$ (MH^+) 563.2177, found 563.2171.

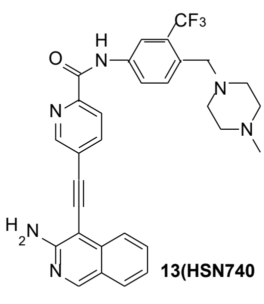

13(HSN740

Compound 13: 5-((3-Aminoisoquinolin-4-yl) ethynyl)-N-(4-((4-methylpiperazin-1-yl) methyl)-3-(trifluoromethyl) phenyl) picolinamide: A solution of Iodo compound **4a** (80 mg, 0.3 mmol, 1 equiv), Pd $(PPh_3)_4$ (10 mol%), CuI (5 mol%) and Triphenylphosphine (10

mg) in Triethylamine (1.5 mL) was de-oxygenated using steam of Argon gas. A de-oxygenated solution of alkyne **7b** (142 mg, 0.35 mmol, 1.2 equiv) in DMF (3 mL) was added slowly over a period of 10 min to the solution and the reaction temperature was increased to 55 ºC and allowed to stir 12 h. The reaction was diluted with ethyl acetate (300 mL). The organic layer was washed with water (5 × 50 mL), saturated NH_4Cl (1 × 50 mL) and brine (1 × 50 mL). Combined organic layers were dried over anhydrous sodium sulphate, filtered and concentrated in vacuo. The pure product **HSN740** was obtained by flash column chromatography. **Yield** = 60%; **TLC R*f*** = 0.2 (5% MeOH/CH_2Cl_2); **^{1}H NMR** (500 MHz, DMSO-*d*$_6$) δ 11.00 (s, 1H), 9.07 (d, *J* = 1.6 Hz, 1H), 8.92 (s, 1H), 8.46 – 8.36 (m, 2H), 8.25 – 8.11 (m, 2H), 8.00 (d, *J* = 8.7 Hz, 1H), 7.91 (d, *J* = 8.1 Hz, 1H), 7.75 – 7.61 (m, 2H), 7.29 (ddd, *J* = 8.0, 6.8, 1.1 Hz, 1H), 6.84 (s, 2H), 3.56 (s, 2H), 2.38 (s, 7H), 2.15 (s, 3H);^{13}C NMR (126 MHz, DMSO-*d*$_6$) δ 163.0, 158., 154., 150.5, 148.0, 140.3, 137.9, 132.8, 132.3, 131.5, 129.2, 128.0 (q, *J* = 28.9 Hz), 125.9(q, *J* = 274.6 Hz), 12 4.2, 123.9, 123.2, 122.9, 122.6, 122.3, 118.0, 96.3, 90.9, 88.4, 57.9, 55.1, 53.0, 46.1; ; **HRMS (ESI+):** calcd. for $C_{30}H_{28}F_3N_6O$ (MH^+) 545.2271, found 545.2275

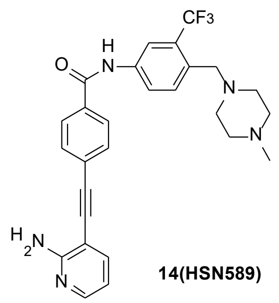

14(HSN589)

Compound 14: 4-((2-Aminopyridin-3-yl) ethynyl)-N-(4-((4-methylpiperazin-1-yl) methyl)-3-(trifluoromethyl) phenyl) benzamide: A solution of Iodo compound **5a** (80 mg, 0.36 mmol, 1 equiv), Pd (PPh_3)$_2$$Cl_2$ (10 mol%), CuI (5 mol%) and Triphenylphosphine (5 mg) in Triethylamine (1.5 mL) was de-oxygenated using steam of Argon gas. A de-oxygenated solution of alkyne **6b** (150 mg, 0.37 mmol, 1 equiv) in DMF (4 mL) was added slowly over a period of 10 min to the solution and the reaction temperature was increased to 55 ºC and allowed to stir 12 h. The reaction was diluted with ethyl acetate (300 mL). The organic layer was washed with water (5 × 50 mL), saturated NH_4Cl (1 × 50 mL), and washed with brine (1 × 50 mL). Combined organic layers were dried over anhydrous sodium sulphate, filtered and concentrated in vacuo. The pure product **HSN589** was obtained by flash column chromatography. **Yield** = 69%.; **TLC R*f*** = 0.5 (10% MeOH/CH_2Cl_2); **^{1}H NMR** (500 MHz, DMSO-*d*$_6$) δ 10.54 (s, 1H), 8.20 (d, *J* = 2.2 Hz, 1H), 8.03 (dd, *J* = 8.5, 2.2 Hz, 1H), 8.02 – 7.97 (m, 3H), 7.80 (d, *J* = 8.4 Hz, 2H), 7.70 (d, *J* = 8.5 Hz, 1H), 7.62 (dd, *J* = 7.5, 1.9 Hz, 1H), 6.57 (dd, *J* = 7.5, 4.9 Hz, 1H), 6.41 (s, 2H), 3.55 (s, 2H), 2.38 (s, 8H), 2.16 (s, 3H); ^{13}C NMR (126 MHz, DMSO-*d*$_6$) δ 165.4, 160.1, 149.3, 140.6, 138.6, 134.1, 132.6, 131.8, 131.7, 128.3, 127.9(q, *J* = 28.9 Hz), 126.5, 125.9(q, *J* = 275.9 Hz), 124.0, 117.7, 112.5, 100.9, 94.3, 88.5, 57.9, 55.1, 53.1, 46.1.**HRMS (ESI+):** calcd. for $C_{27}H_{27}F_3N_5O$ (MH^+) 499.2162, found 499.2162.

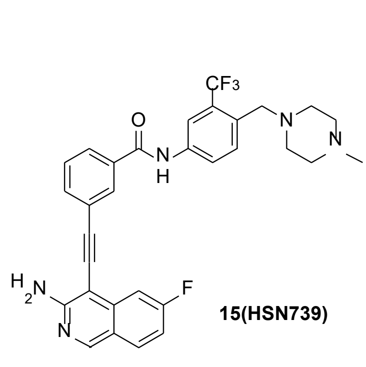

15(HSN739)

Compound 15: 3-((3-Amino-6-fluoroisoquinolin-4-yl) ethynyl)-N-(4-((4-methylpiperazin-1-yl) methyl)-3-(trifluoromethyl) phenyl) benzamide: A solution of Iodo compound **2a** (100 mg, 0.35 mmol, 1 equiv), Pd (PPh_3)$_4$ (10 mol%), CuI (5 mol%) and Triphenylphosphine (10 mg) in Triethylamine (1 mL) was de- oxygenated using steam of Argon gas. A de-oxygenated solution of alkyne **8b** (170 mg, 0.42 mmol, 1.2 equiv) in DMF (4 mL) was added slowly over a period of 10 min to the solution and the re- action temperature was increased to 55 ºC and allowed to stir 12 h. The reaction was diluted with ethyl acetate (300 mL). The organic layer was washed with

water (5 × 50 mL), saturated NH_4Cl (1 × 50 mL) and brine (1 × 50 mL). Combined organic layers were dried over anhydrous sodium sulphate, filtered and concentrated in vacuo. The pure product **HSN739** was obtained by flash column chromatography. **Yield** = 71%; **TLC R*f*** = 0.2 (10% MeOH/CH_2Cl_2); **^{1}H NMR** (500 MHz, DMSO-d_6) δ 10.61 (s, 1H), 8.89 (s, 1H), 8.34 (s, 1H), 8.22 (d, *J* = 2.2 Hz, 1H), 8.09 – 7.95 (m, 3H), 7.93 (d, *J* = 8.0 Hz, 1H), 7.71 (d, *J* = 8.5 Hz, 1H), 7.65 – 7.55 (m, 2H), 7.15 (td, *J* = 8.8, 2.5 Hz, 1H), 6.81 (s, 2H), 3.56 (s, 2H), 2.38 (s, 8H), 2.15 (s, 3H); ^{13}C NMR (126 MHz, DMSO-d_6) δ 165.7, 165.6 (d, *J* = 249.4 Hz), 158.5, 153.0, 139.8, 138.6, 135.4, 134.8, 132.9, 132.6, 131.6, 130.7, 129.2, 127.9, 127.7, 125.9(q, *J* = 274.6 Hz), 123.9, 123.7, 119.7, 117.7, 113.1, 106.3, 99.3, 89.1, 84.9, 57.9, 55.1, 53.1, 46.1.; **HRMS (ESI+):** calcd. for $C_{31}H_{28}F_4N_5O$ (MH^+) 562.2224, found 562.2222.

HPLC Traces of Compounds

Conditions: Agilent Eclipse plus C18 column, 3,5 μm, 4.6×100 mm, 0→15 min, 50% B→100% B(A: 0.1% NH_4OH in H_2O, B: MeOH), 50 ^{o}C.

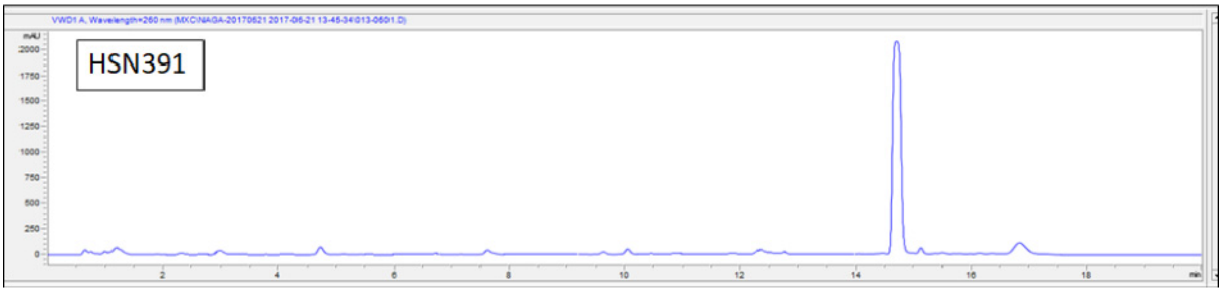

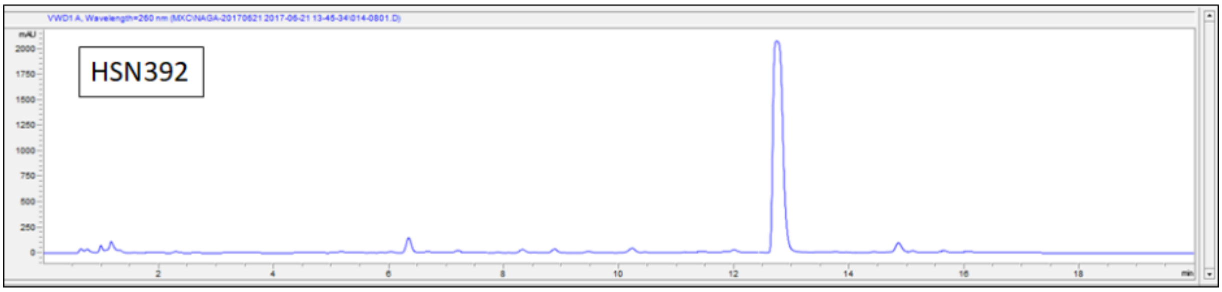

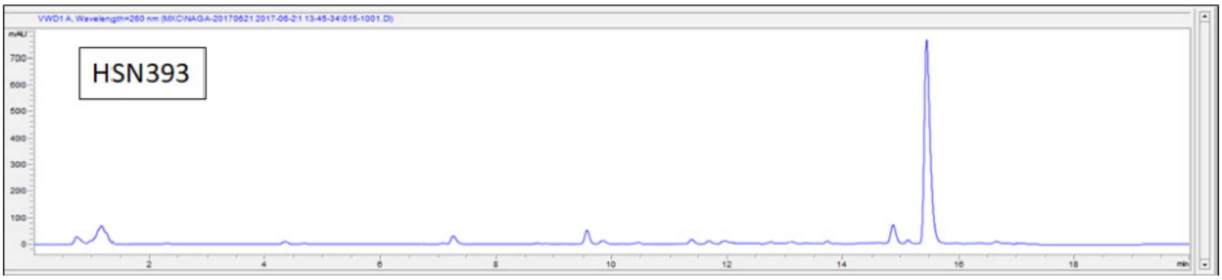

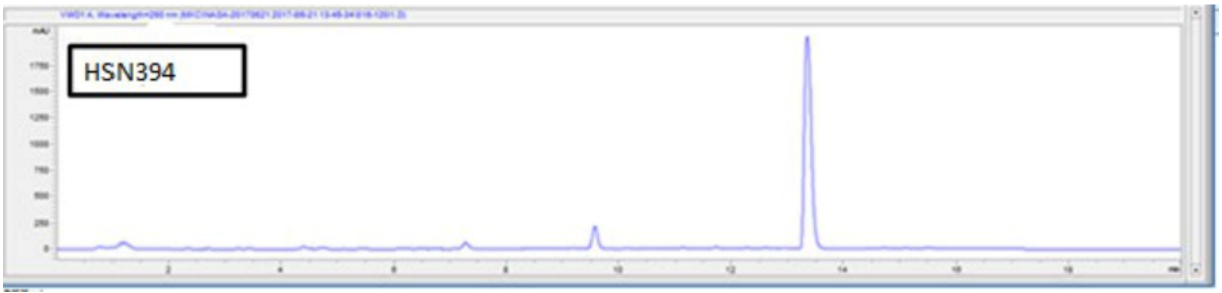
HSN394

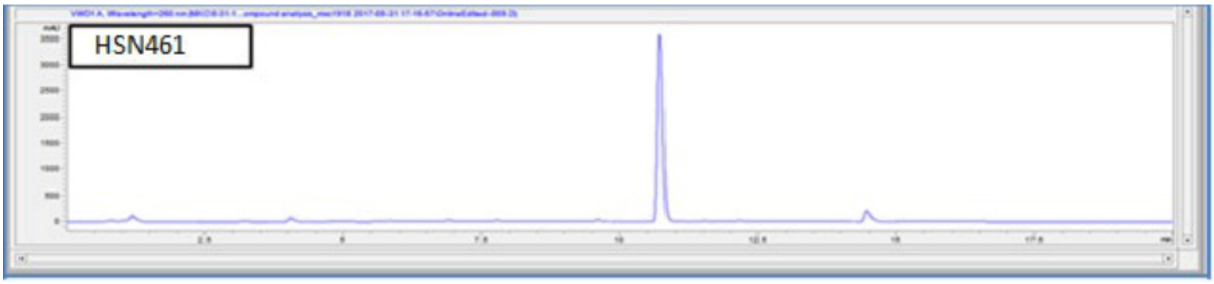
HSN461

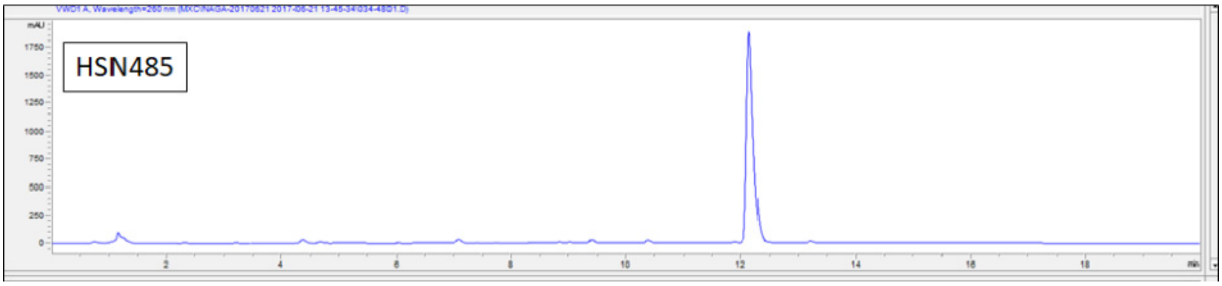
HSN485

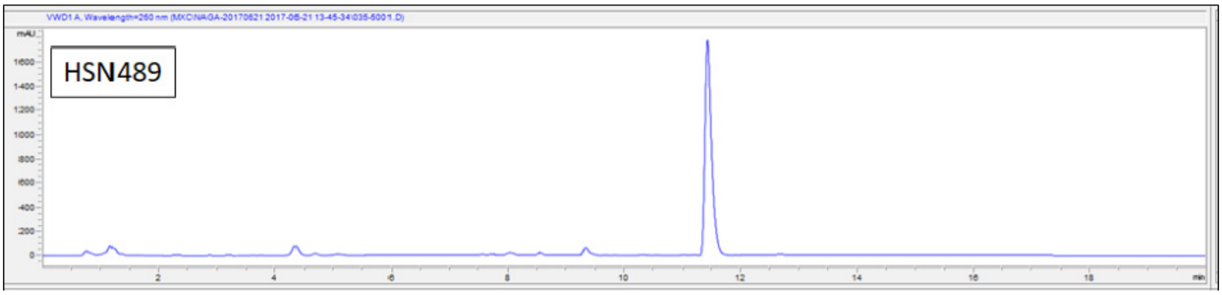
HSN489

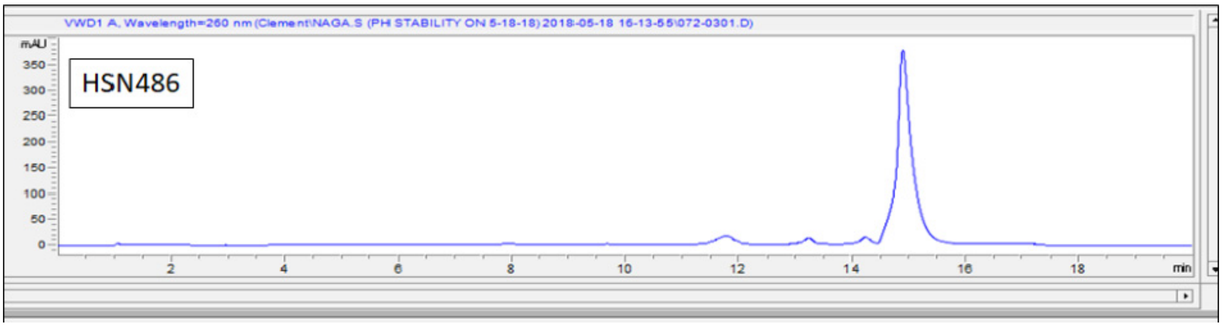
VWD1 A, Wavelength=260 nm (Clement\NAGA.S (PH STABILITY ON 5-18-18) 2018-05-18 16-13-55\072-0301.D)
HSN486

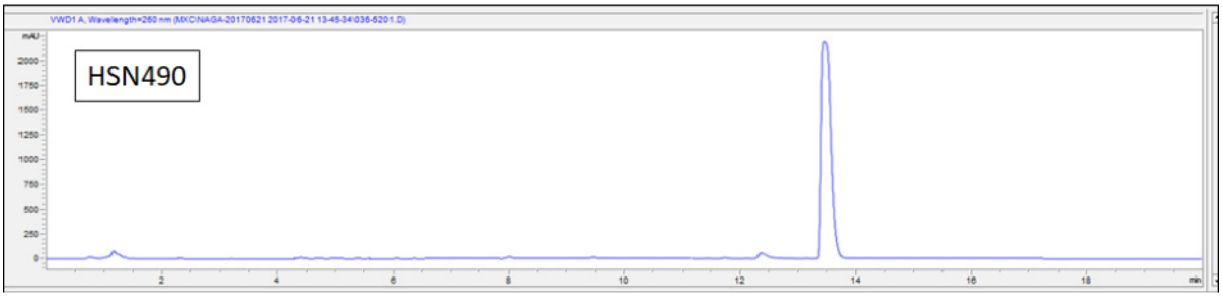
HSN490

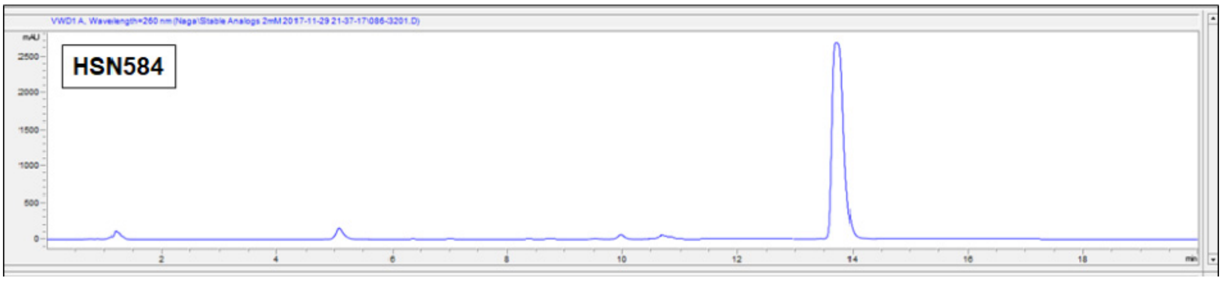
HSN584

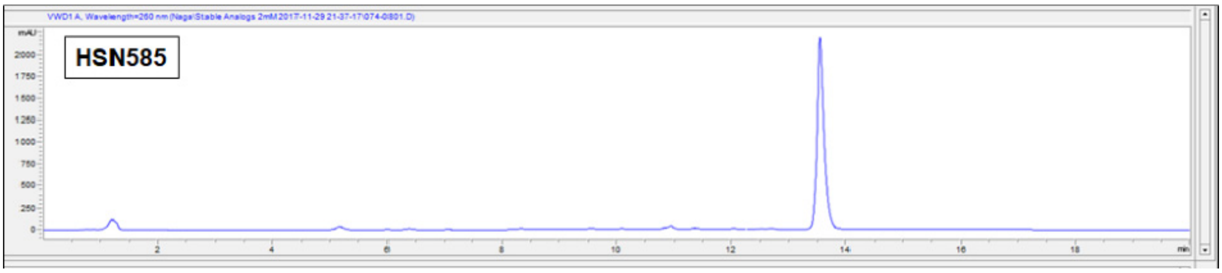
HSN585

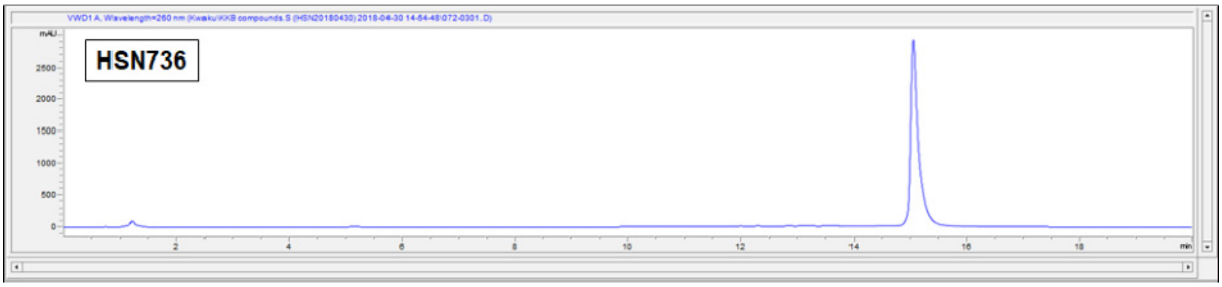
HSN736

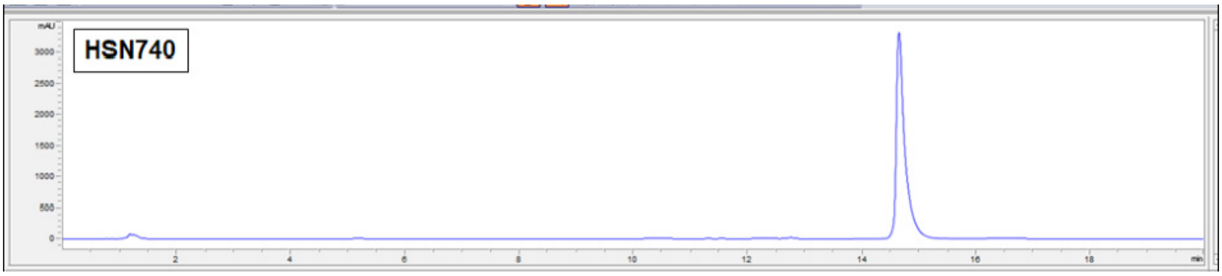
HSN740

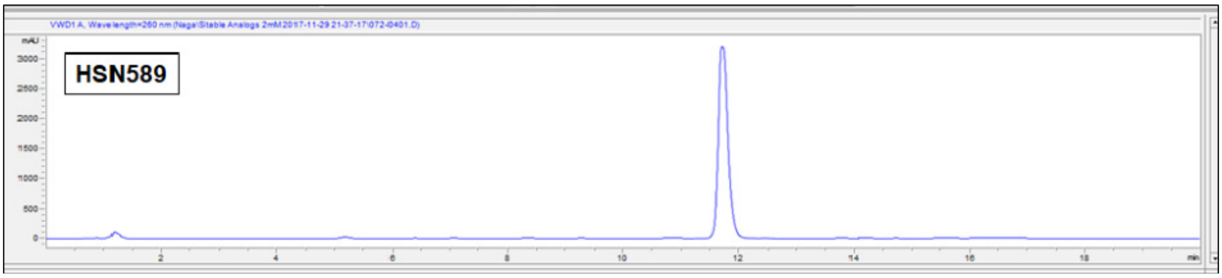
VWD1 A, Wavelength=260 nm (Naga\Stable Analogs 2mM 2017-11-29 21-37-17\072-0401.D)
HSN589
mAU
3000
2500
2000
1500
1000
500
0
2
4
6
8
10
12
14
16
18
min

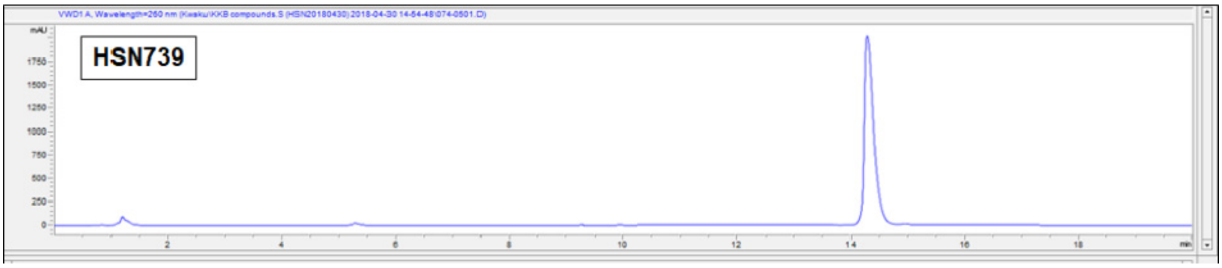
VWD1 A, Wavelength=260 nm (Kwaku\KKB compounds.S (HSN20180430) 2018-04-30 14-54-48\074-0501.D)
HSN739
mAU
1750
1500
1250
1000
750
500
250
0
2
4
6
8
10
12
14
16
18
min

NMR Spectra of Compounds

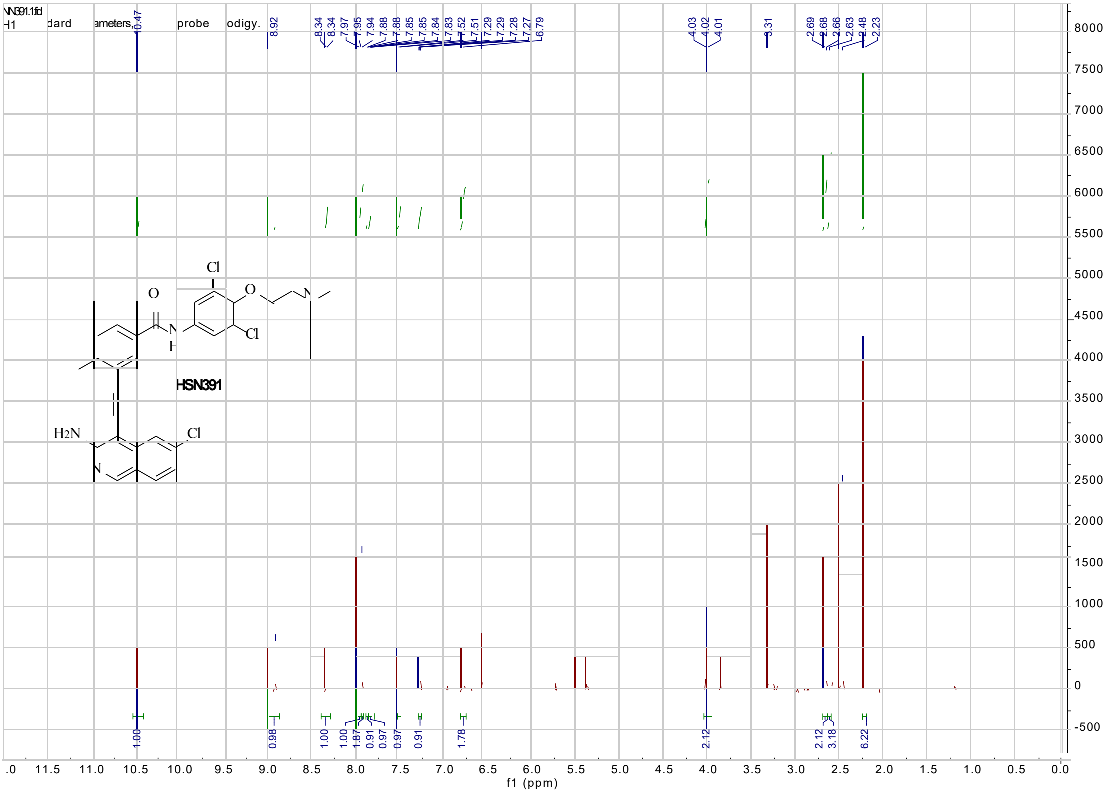

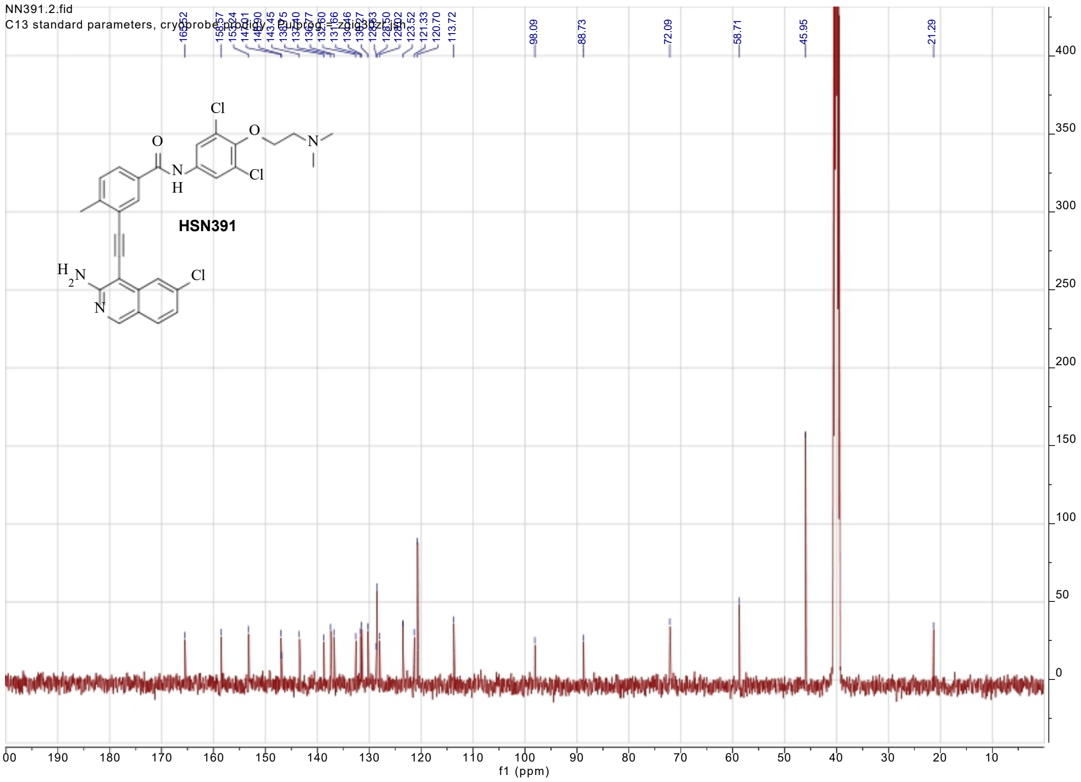

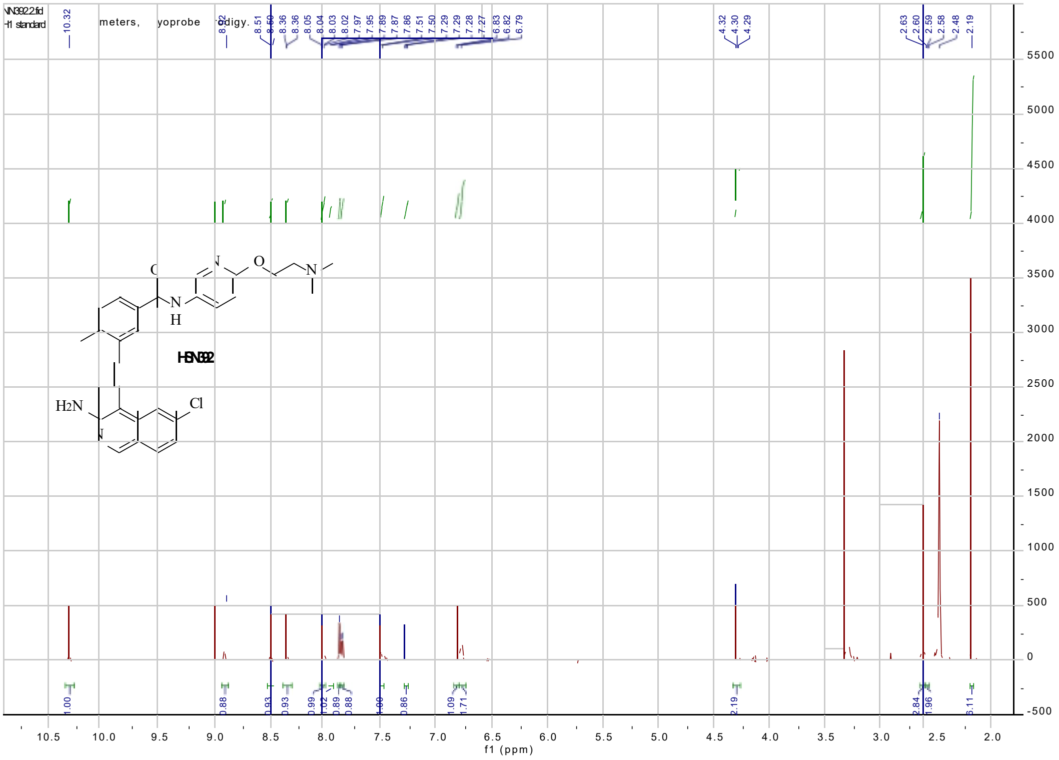
HSN392
H2N
Cl
f1 (ppm)

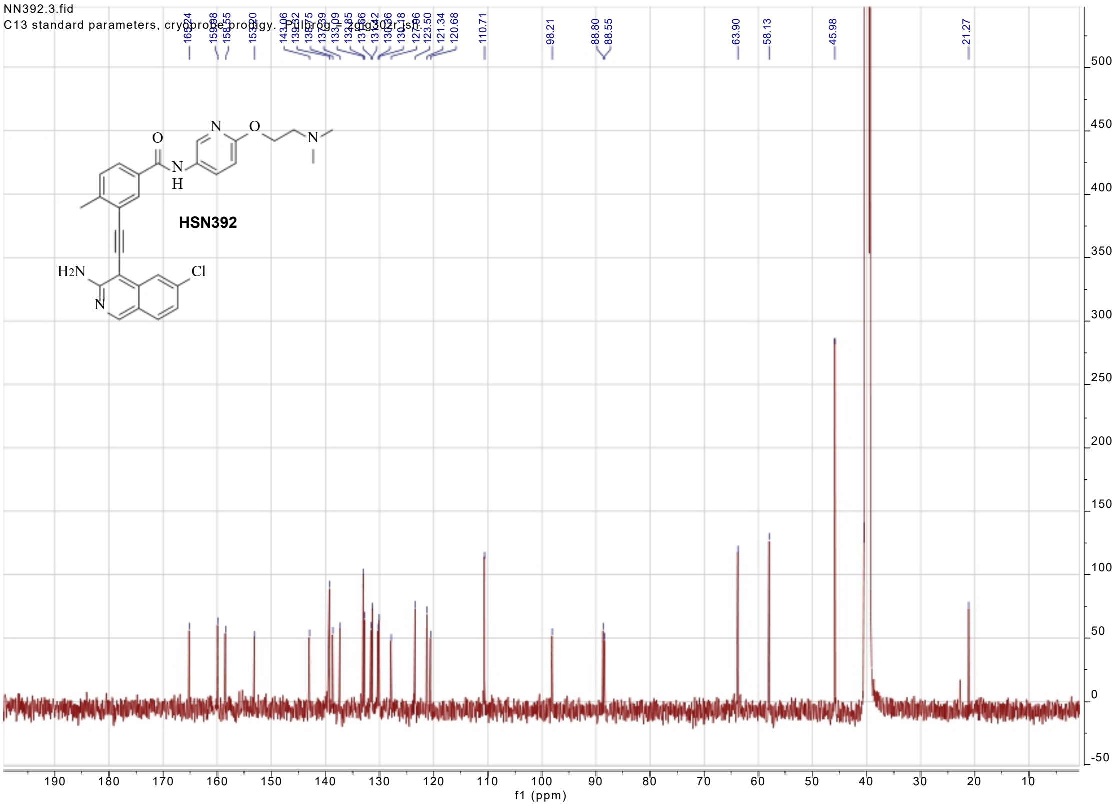
NN392.3.fid
HSN392
H2N
Cl
f1 (ppm)

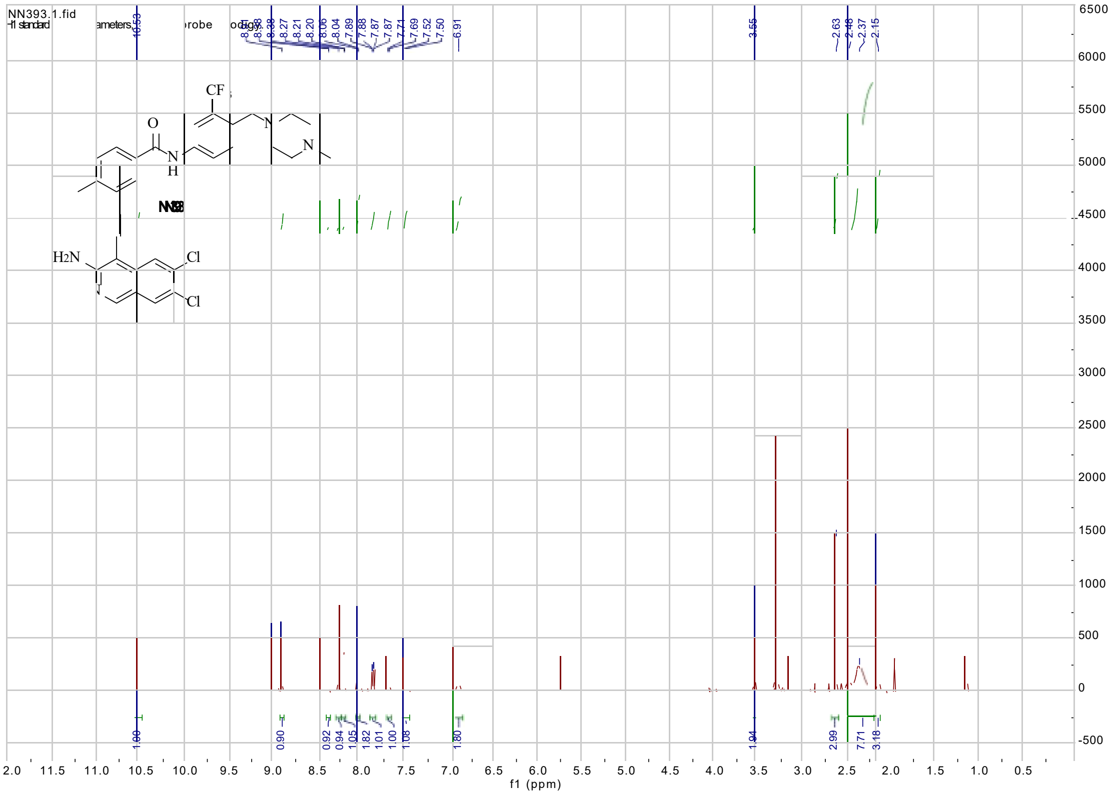
NN393.1.fid
NN393
f1 (ppm)

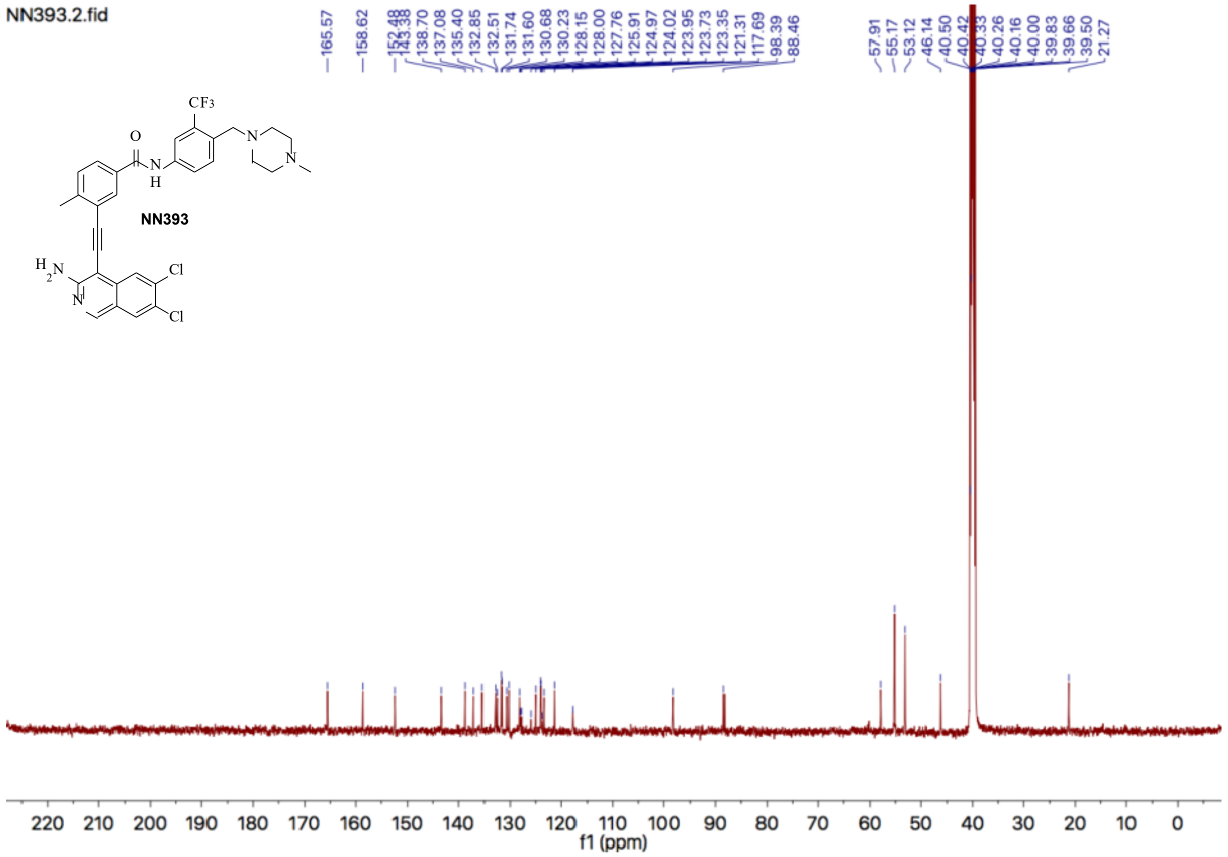
NN393.2.fid
CF3
O
N
H
N
N
NN393
H2N
N
Cl
Cl
f1 (ppm)

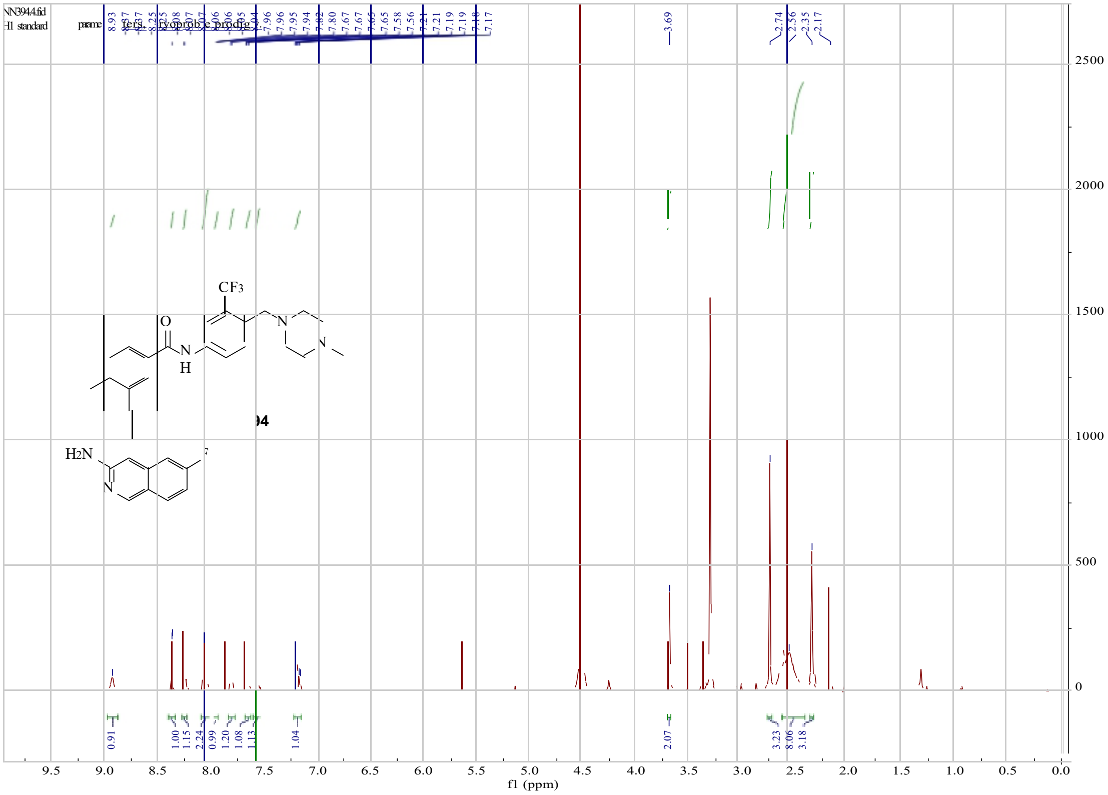
NN3944.fid
H1 standard
CF3
O
N
H
N
N
H2N
N
F
3.69
2.74
2.56
2.35
2.17
0.91
1.04
2.07
3.23
8.06
3.18
2500
2000
1500
1000
500
0
f1 (ppm)

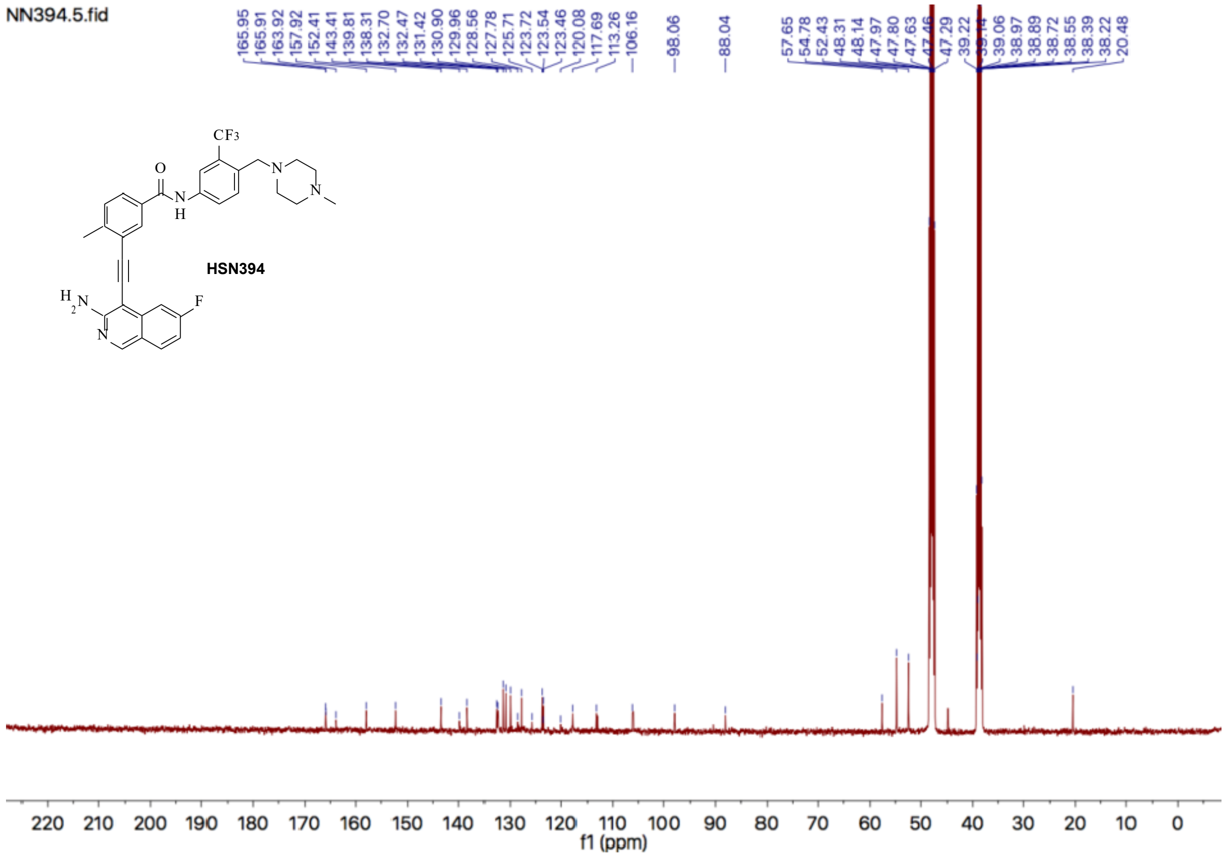
NN394.5.fid
165.95
165.91
163.92
157.92
152.41
143.41
139.81
138.31
132.70
132.47
131.42
130.90
129.96
128.56
127.78
125.71
123.72
123.54
123.46
120.08
117.69
113.26
106.16
98.06
88.04
57.65
54.78
52.43
48.31
48.14
47.97
47.80
47.63
47.29
39.22
39.06
38.97
38.89
38.72
38.55
38.39
38.22
20.48
CF3
O
N
H
N
N
HSN394
H2N
F
N
f1 (ppm)

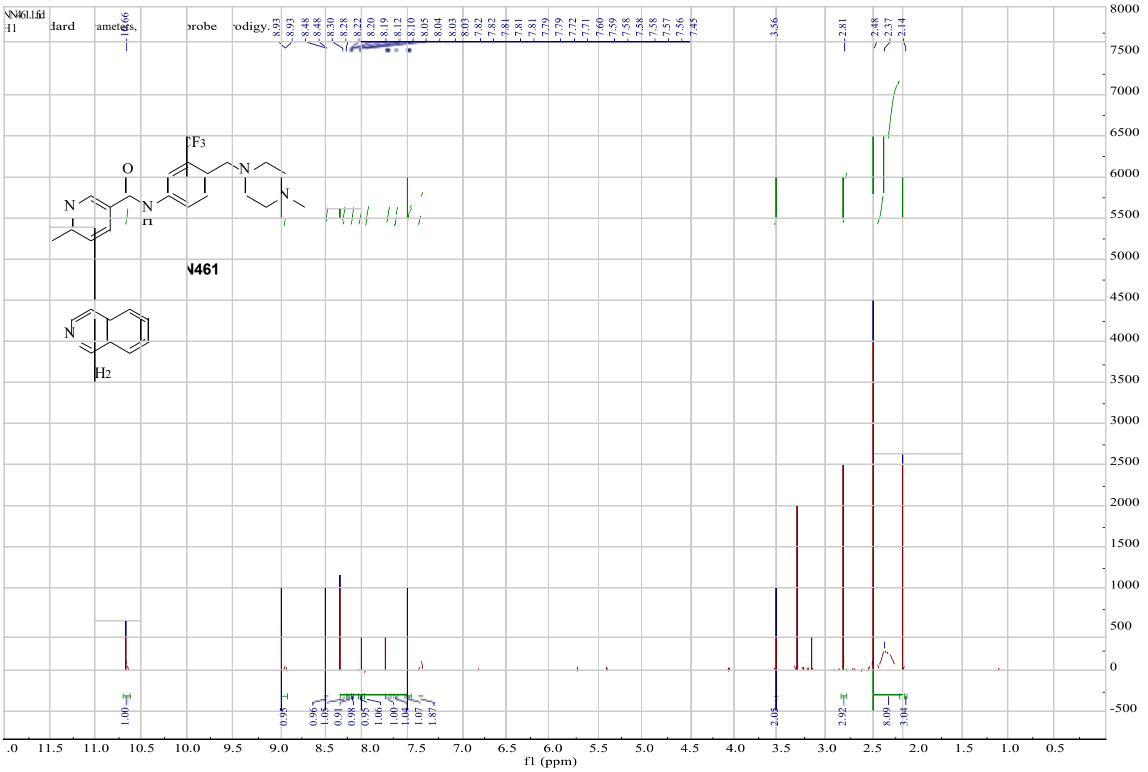
NN461.1.fid
HSN461
f1 (ppm)

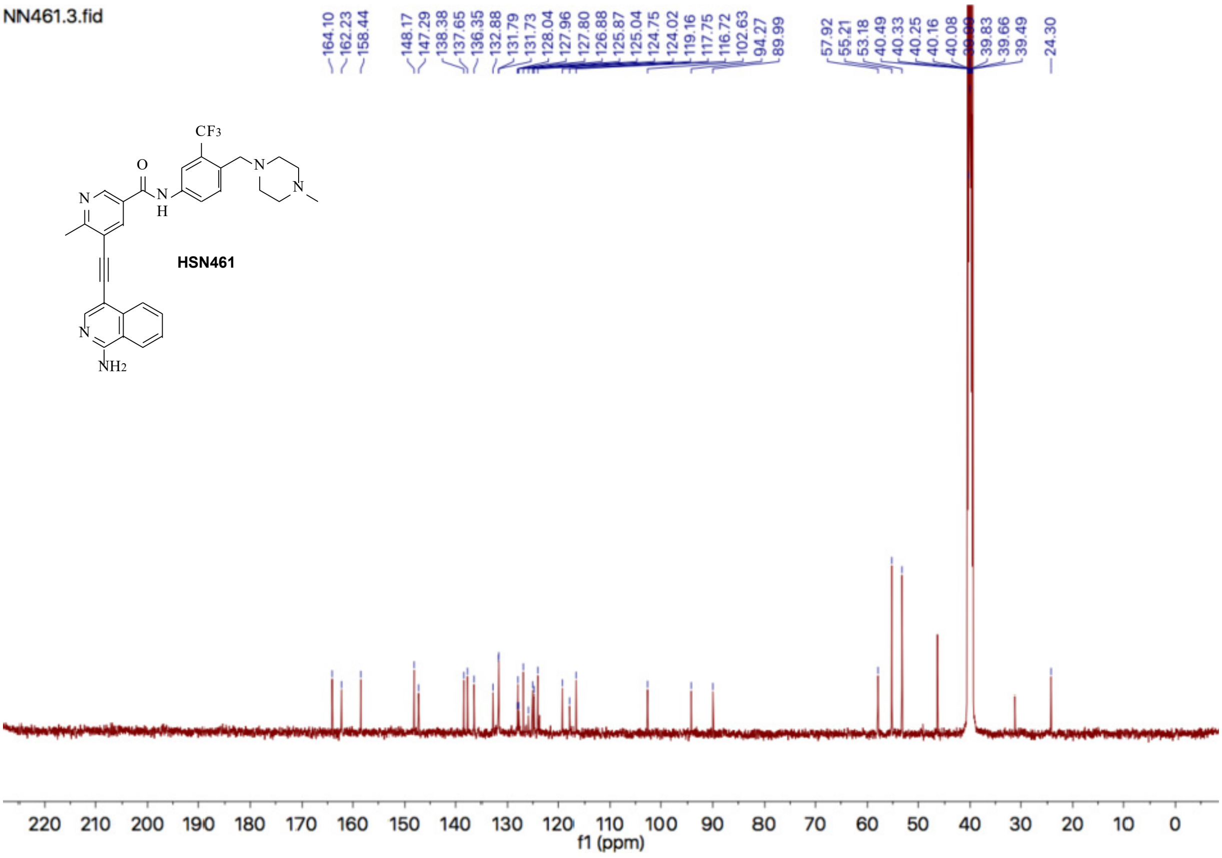
NN461.3.fid
164.10
162.23
158.44
148.17
147.29
138.38
137.65
136.35
132.88
131.79
131.73
128.04
127.96
127.80
126.88
125.87
125.04
124.75
124.02
119.16
117.75
116.72
102.63
94.27
89.99
57.92
55.21
53.18
40.49
40.33
40.25
40.16
40.08
39.83
39.66
39.49
24.30
CF3
O
N
H
N
N
N
N
NH2
HSN461
f1 (ppm)

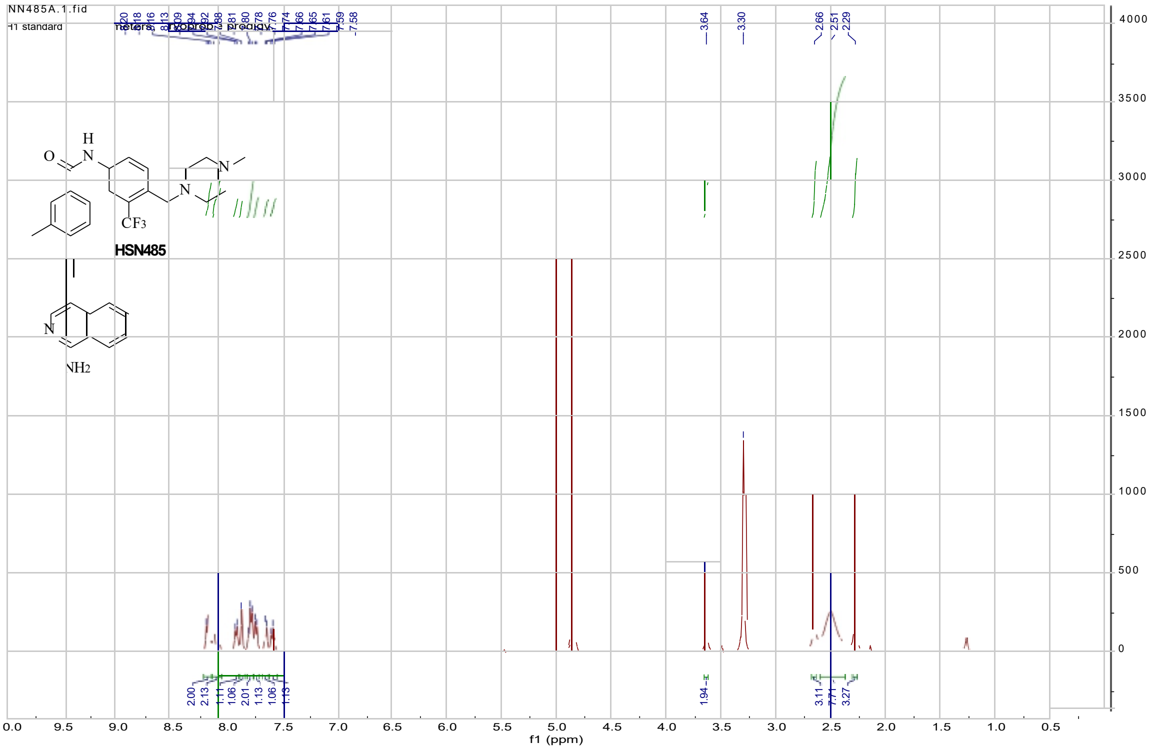
NN485A.1.fid
1H standard
HSN485
f1 (ppm)

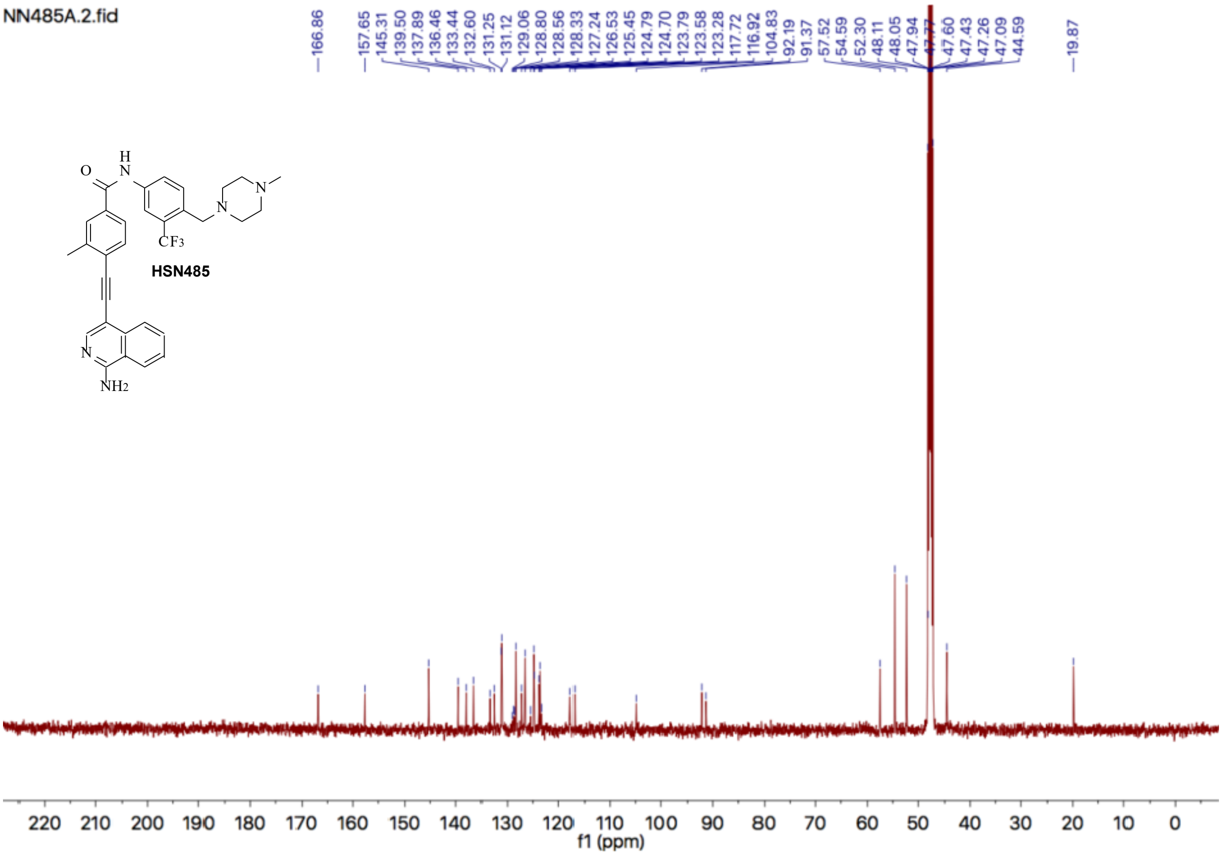
NN485A.2.fid
HSN485
f1 (ppm)

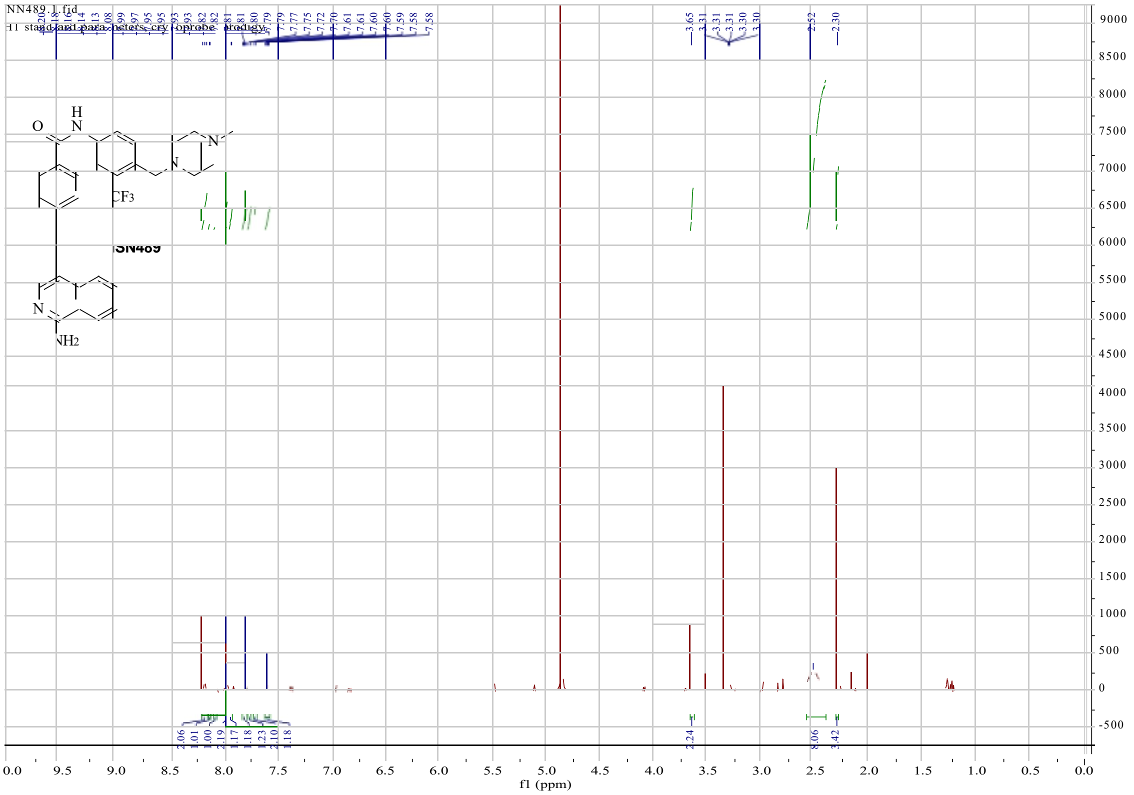
NN489.1.fid
3.65
3.31
3.31
3.31
3.30
3.30
2.52
2.30
f1 (ppm)

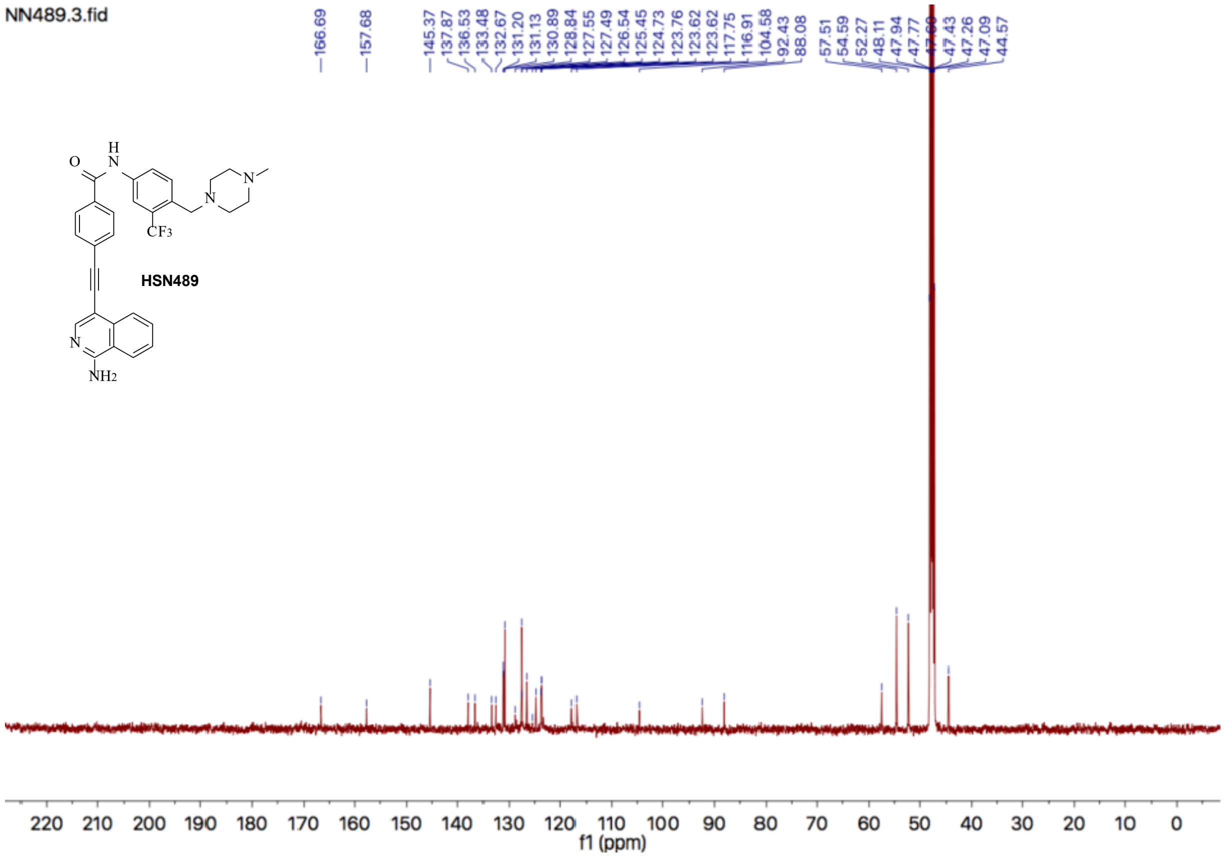
NN489.3.fid
166.69
157.68
145.37
137.87
136.53
133.48
132.67
131.20
131.13
130.89
128.84
127.55
127.49
126.54
125.45
124.73
123.76
123.62
123.62
117.75
116.91
104.58
92.43
88.08
57.51
54.59
52.27
48.11
47.94
47.77
47.43
47.26
47.09
44.57
HSN489
f1 (ppm)

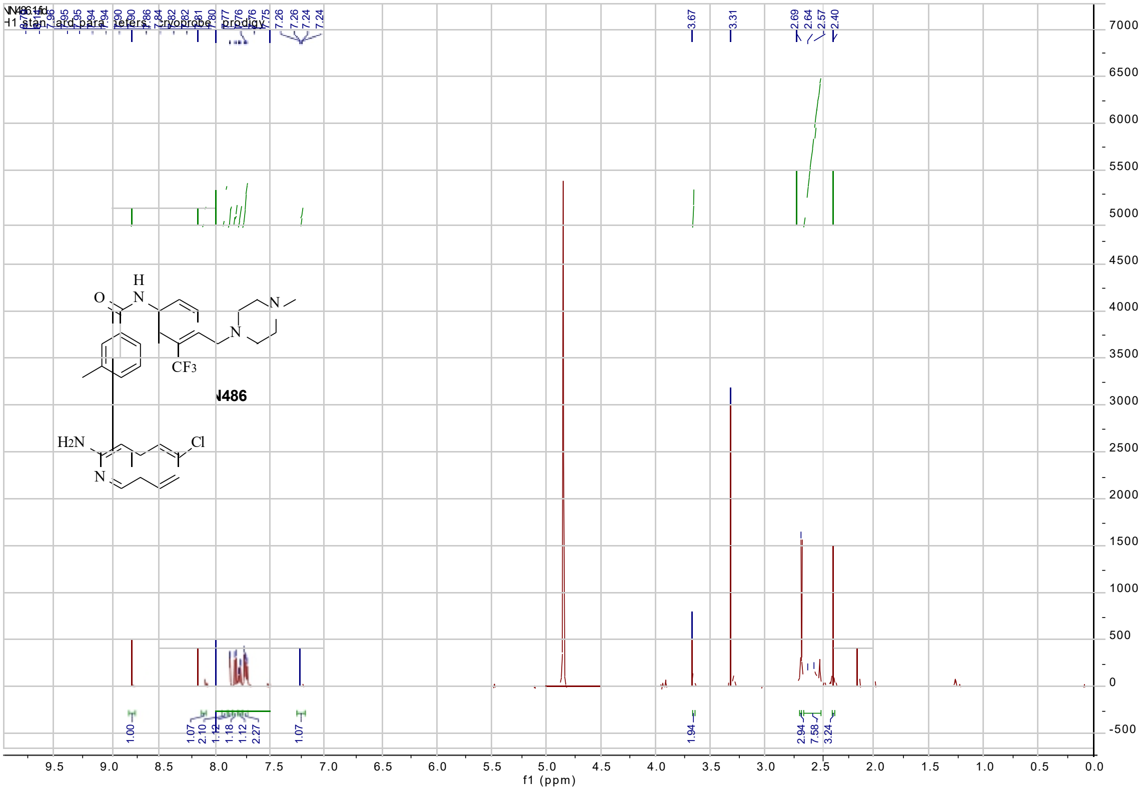
3.67
3.31
2.69
2.64
2.57
2.40
1.00
1.07
2.10
1.12
1.18
1.12
2.27
1.07
1.94
2.94
7.58
3.24
H
O
N
N
N
CF3
H2N
Cl
N
f1 (ppm)

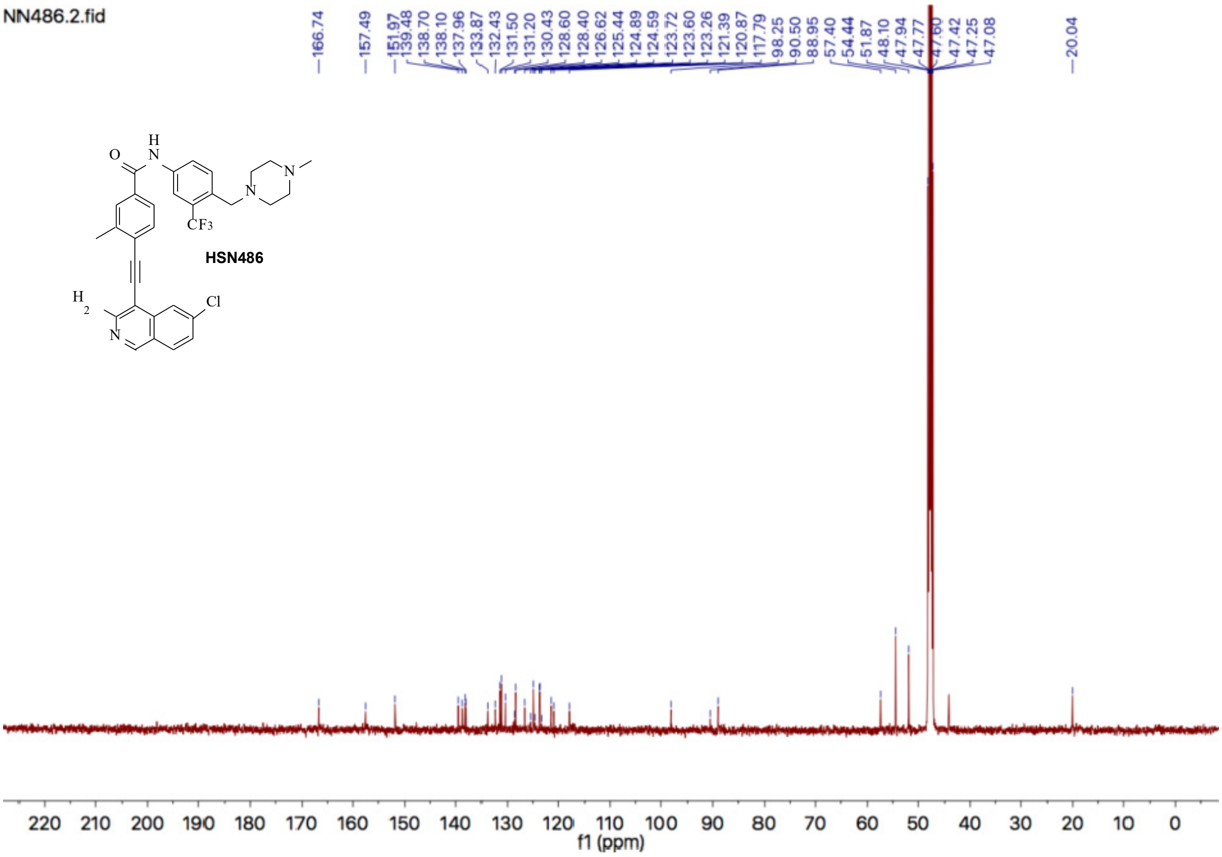
NN486.2.fid
166.74
157.49
151.97
139.48
138.70
138.10
137.96
133.87
132.43
131.50
131.20
130.43
128.60
128.40
126.62
125.44
124.89
124.59
123.72
123.60
123.26
121.39
120.87
117.79
98.25
90.50
88.95
57.40
54.44
51.87
48.10
47.94
47.77
47.60
47.42
47.25
47.08
20.04
H
O
N
N
N
CF3
HSN486
Cl
N
f1 (ppm)

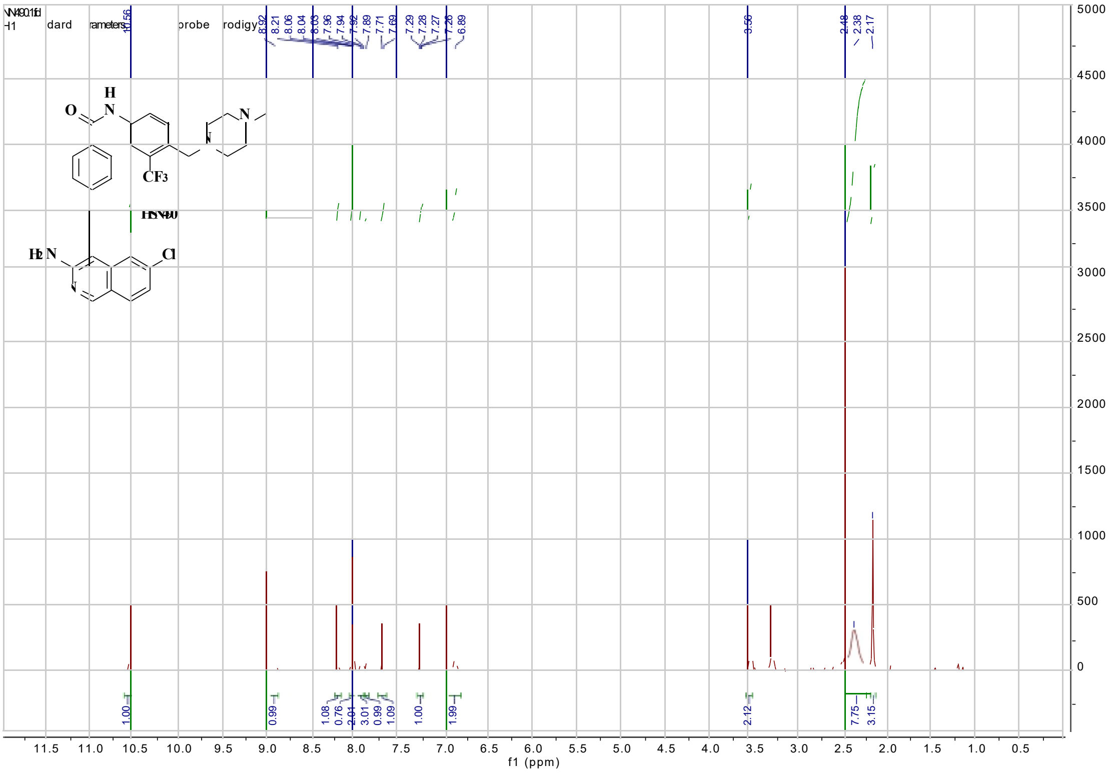
HSN490
10.56
8.92
8.21
8.06
8.04
8.03
7.96
7.94
7.92
7.89
7.71
7.69
7.29
7.28
7.27
7.26
6.89
3.50
2.48
2.38
2.17
f1 (ppm)

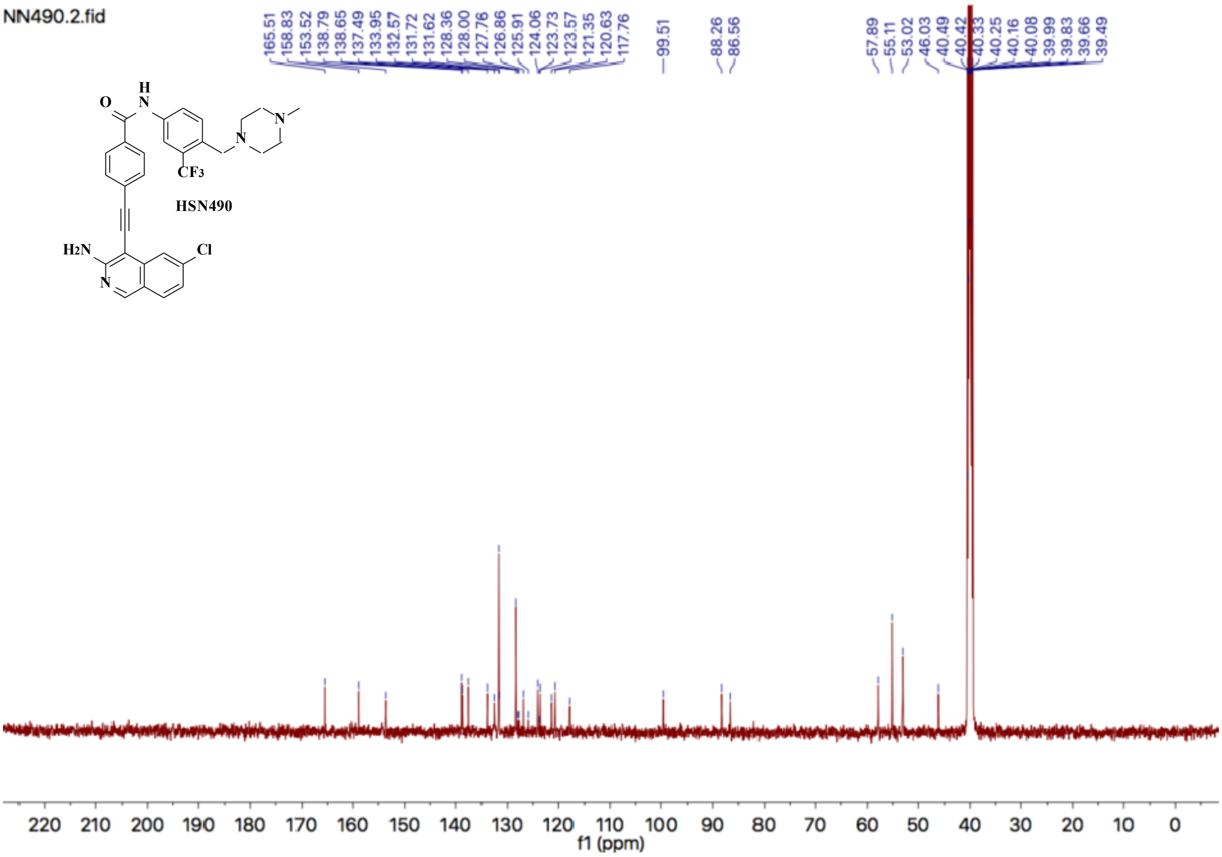
NN490.2.fid
HSN490
165.51
158.83
153.52
138.79
138.65
137.49
133.95
132.57
131.72
131.62
128.36
128.00
127.76
126.86
125.91
124.06
123.73
123.57
121.35
120.63
117.76
99.51
88.26
86.56
57.89
55.11
53.02
46.03
40.49
40.42
40.33
40.25
40.16
40.08
39.99
39.83
39.66
39.49
f1 (ppm)

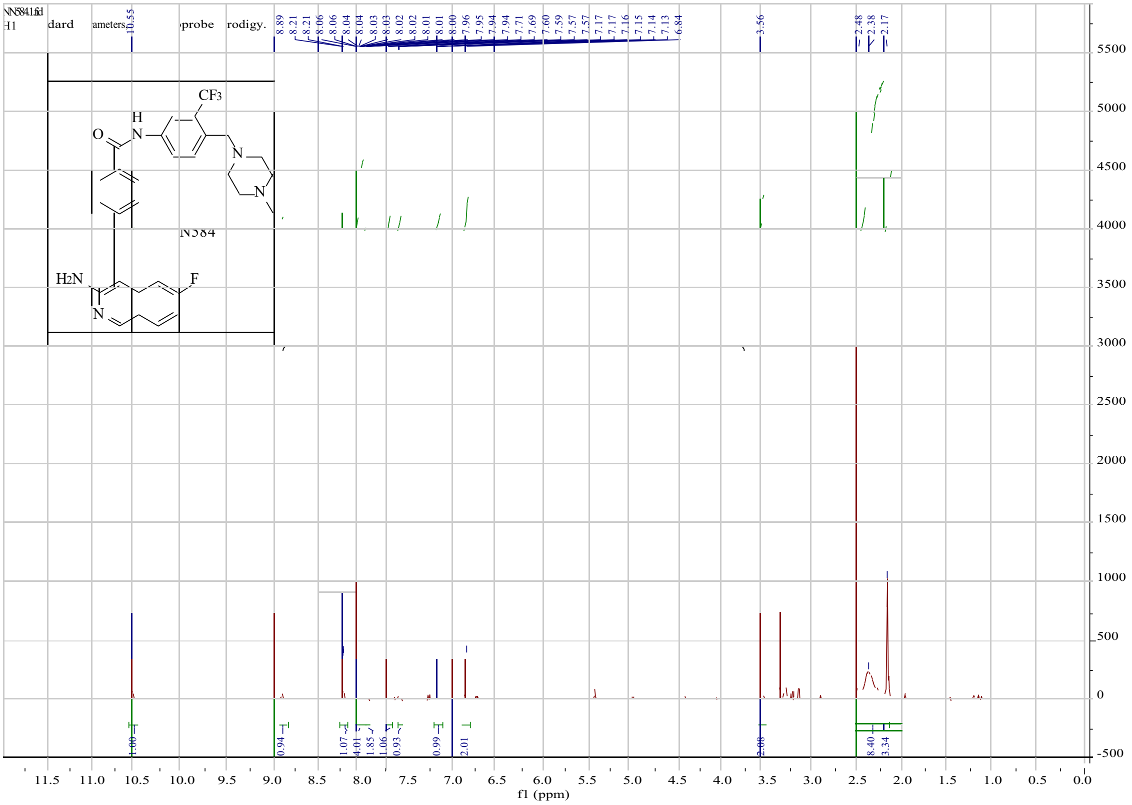

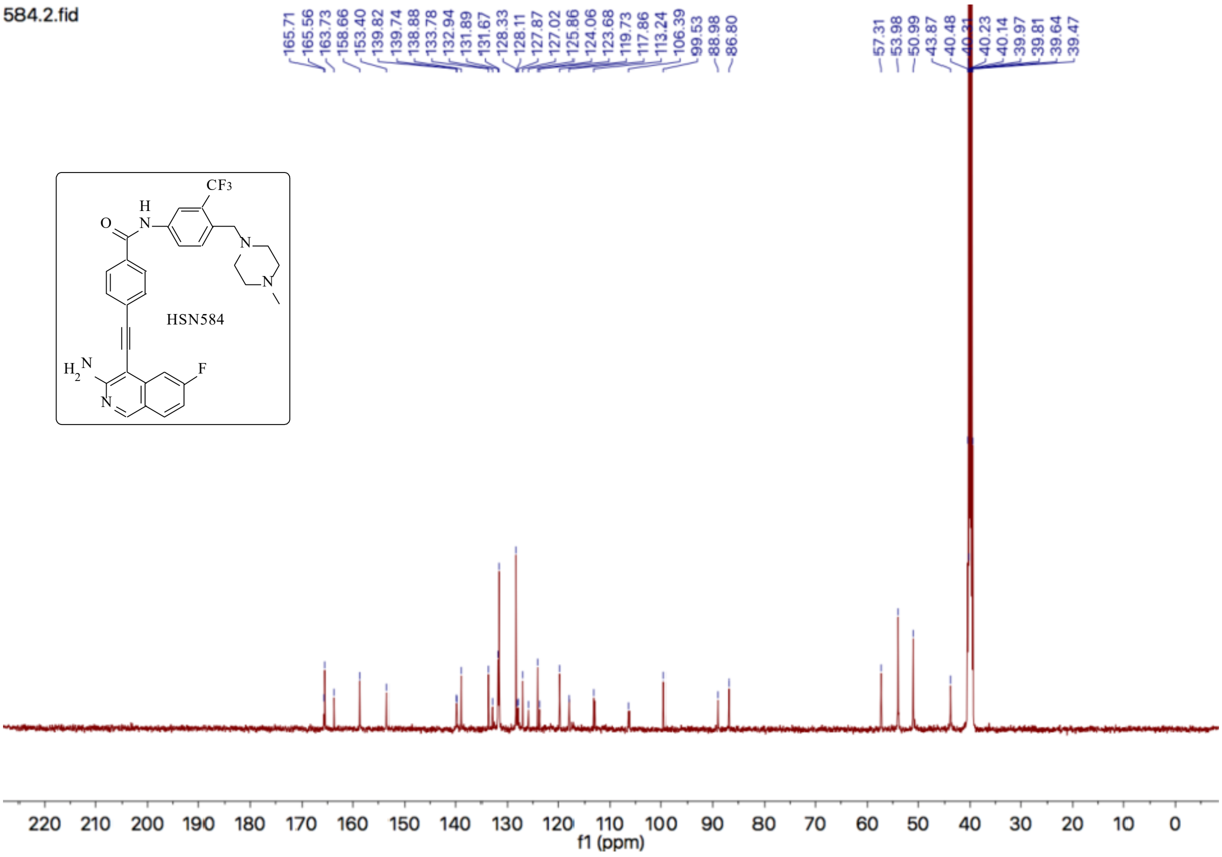

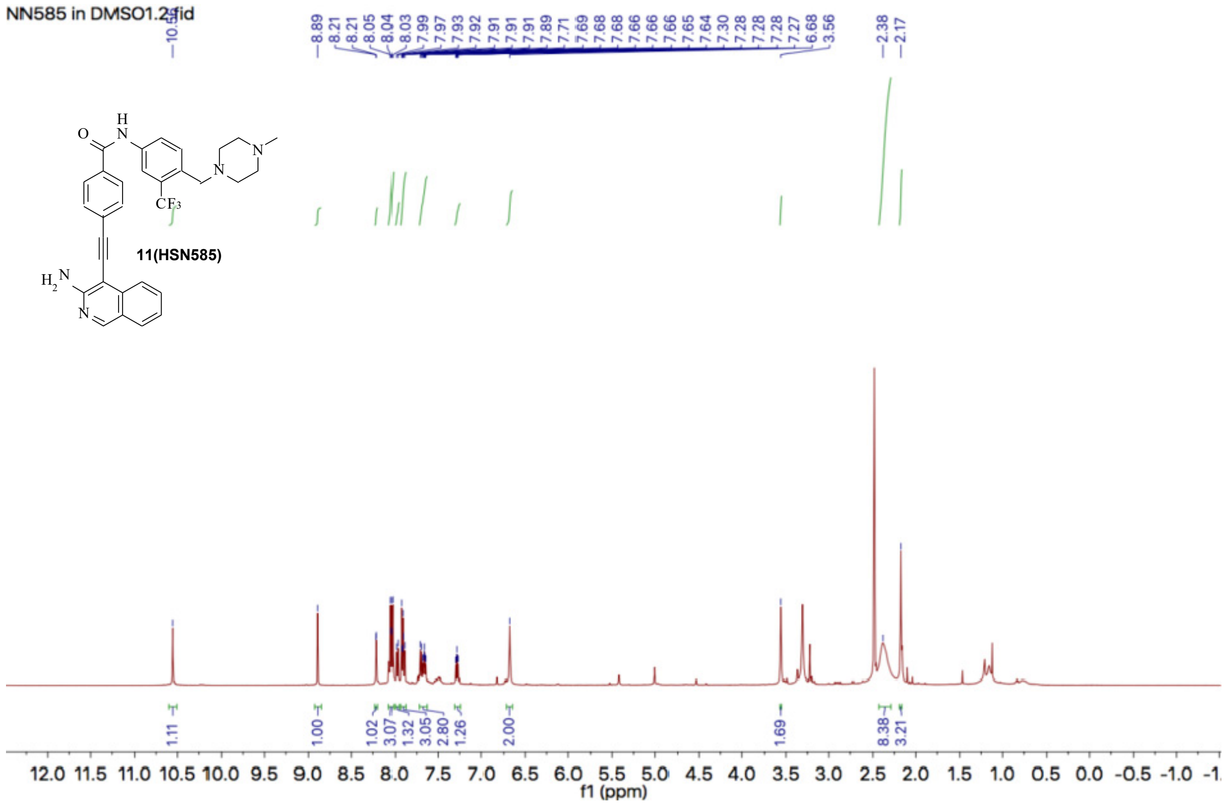
NN585 in DMSO1.2.fid
11(HSN585)
10.56 8.89 8.21 8.05 8.04 8.03 7.99 7.97 7.93 7.92 7.91 7.91 7.91 7.89 7.71 7.69 7.68 7.68 7.66 7.66 7.66 7.65 7.64 7.30 7.28 7.28 7.28 7.27 6.68 3.56 2.38 2.17
1.11 1.00 1.02 3.07 1.32 3.05 2.80 1.26 2.00 1.69 8.38 3.21
f1 (ppm)

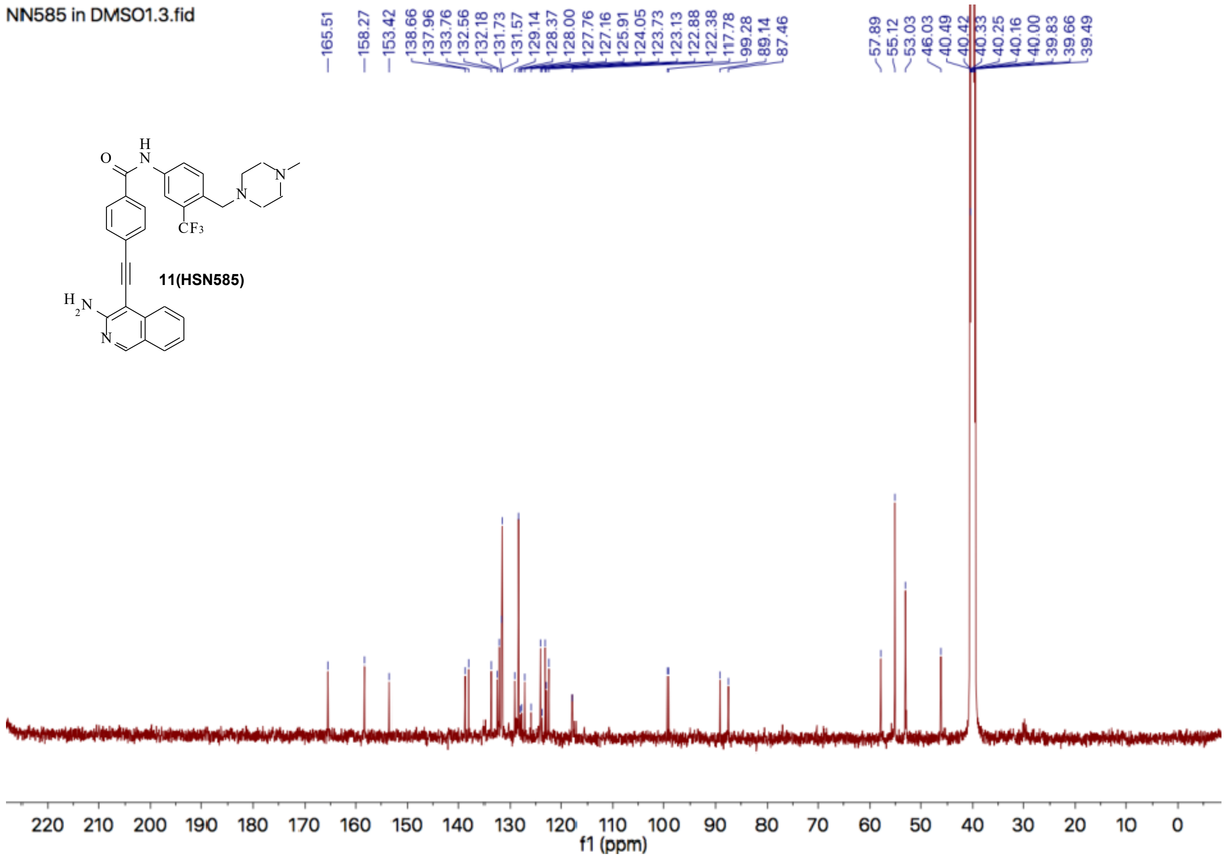
NN585 in DMSO1.3.fid
11(HSN585)
165.51 158.27 153.42 138.66 137.96 133.76 132.56 132.18 131.73 131.57 129.14 128.37 128.00 127.76 127.16 125.91 124.05 123.73 123.13 122.88 122.38 117.78 99.28 89.14 87.46 57.89 55.12 53.03 46.03 40.49 40.42 40.33 40.25 40.16 40.00 39.83 39.66 39.49
f1 (ppm)

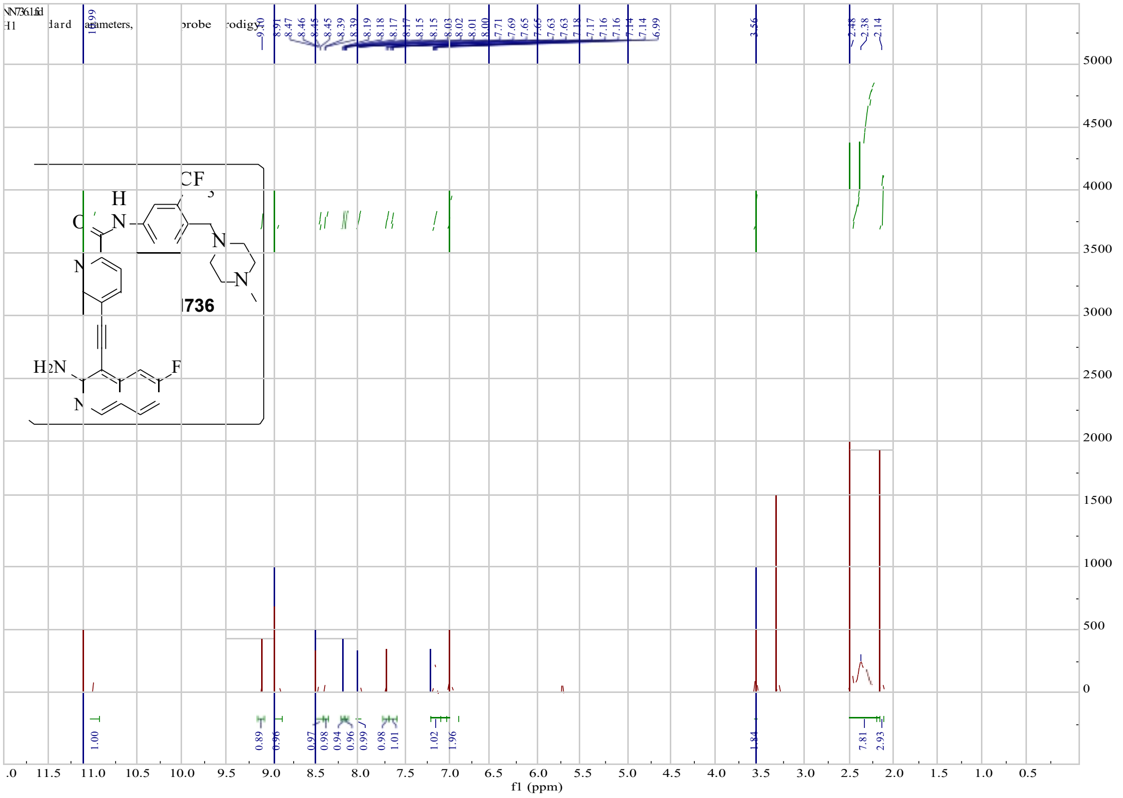
f1 (ppm)

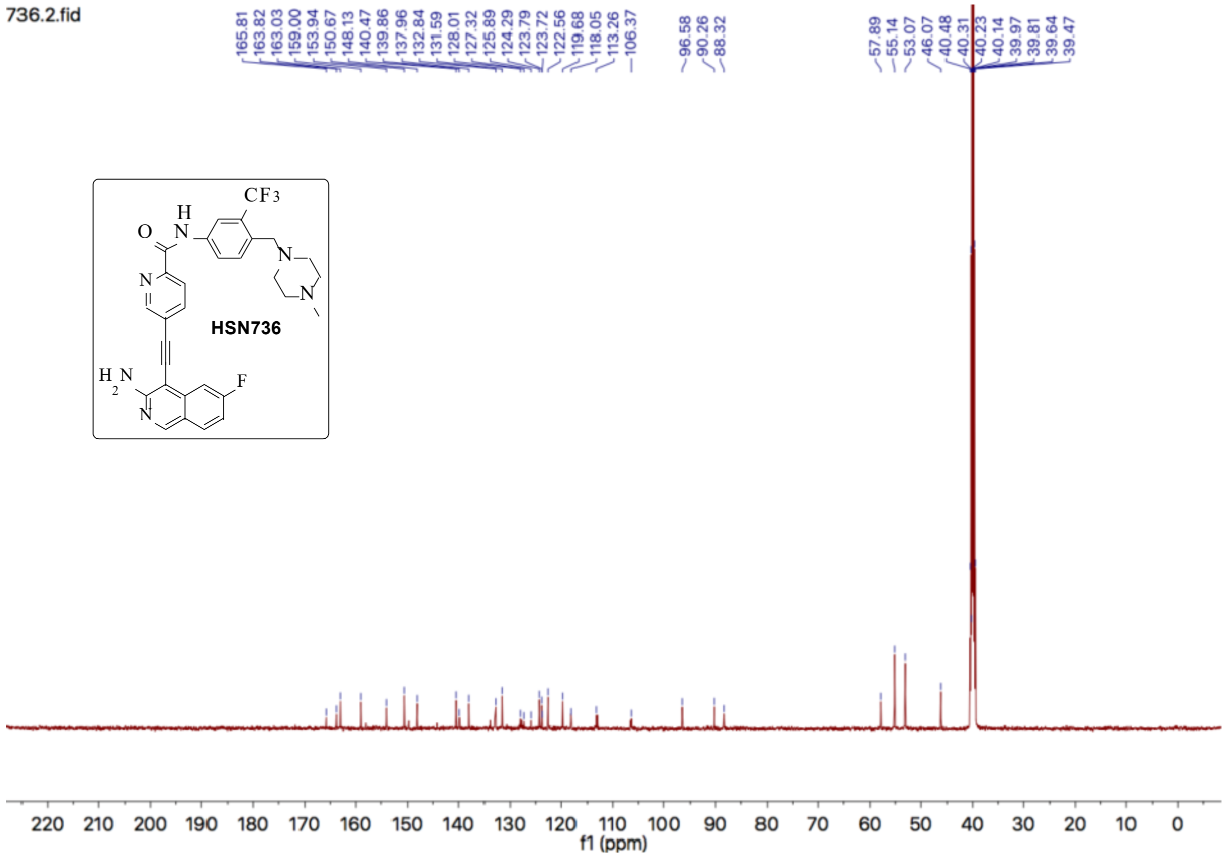
736.2.fid
165.81
163.82
163.03
159.00
153.94
150.67
148.13
140.47
139.86
137.96
132.84
131.59
128.01
127.32
125.89
124.29
123.79
123.72
122.56
119.68
118.05
113.26
106.37
96.58
90.26
88.32
57.89
55.14
53.07
46.07
40.48
40.31
40.23
40.14
39.97
39.81
39.64
39.47
HSN736
f1 (ppm)

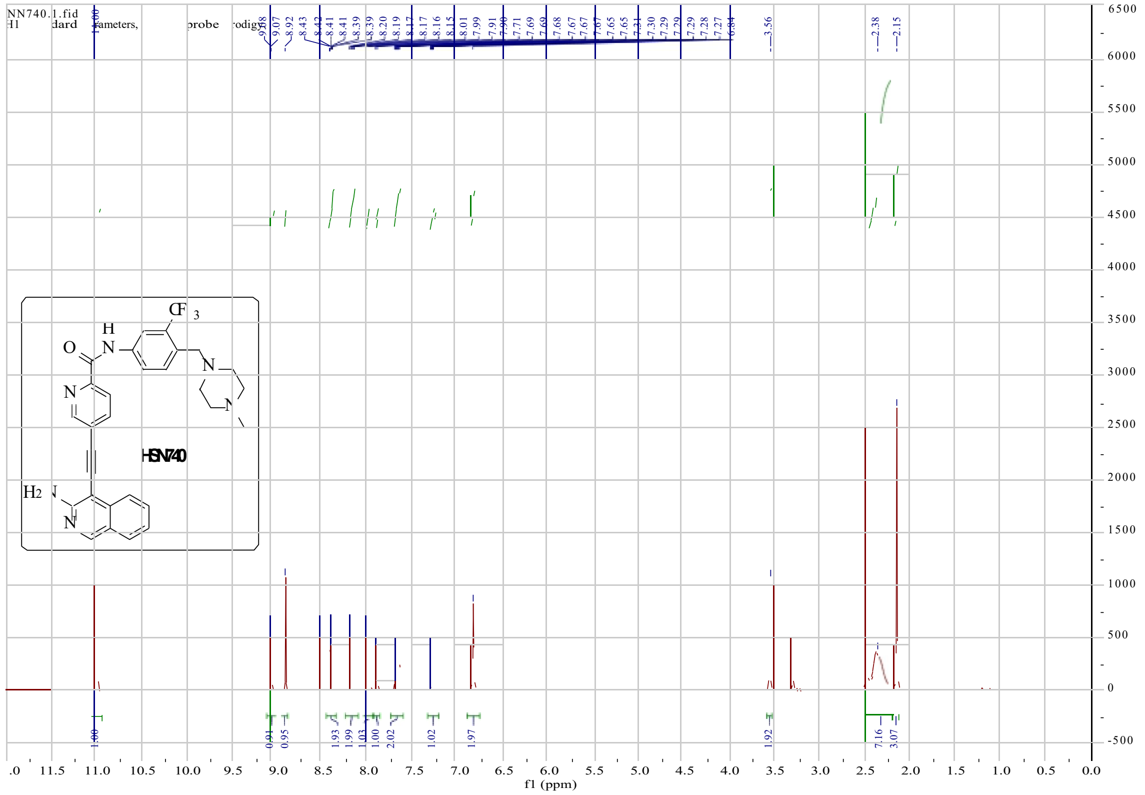
HSN740
f1 (ppm)

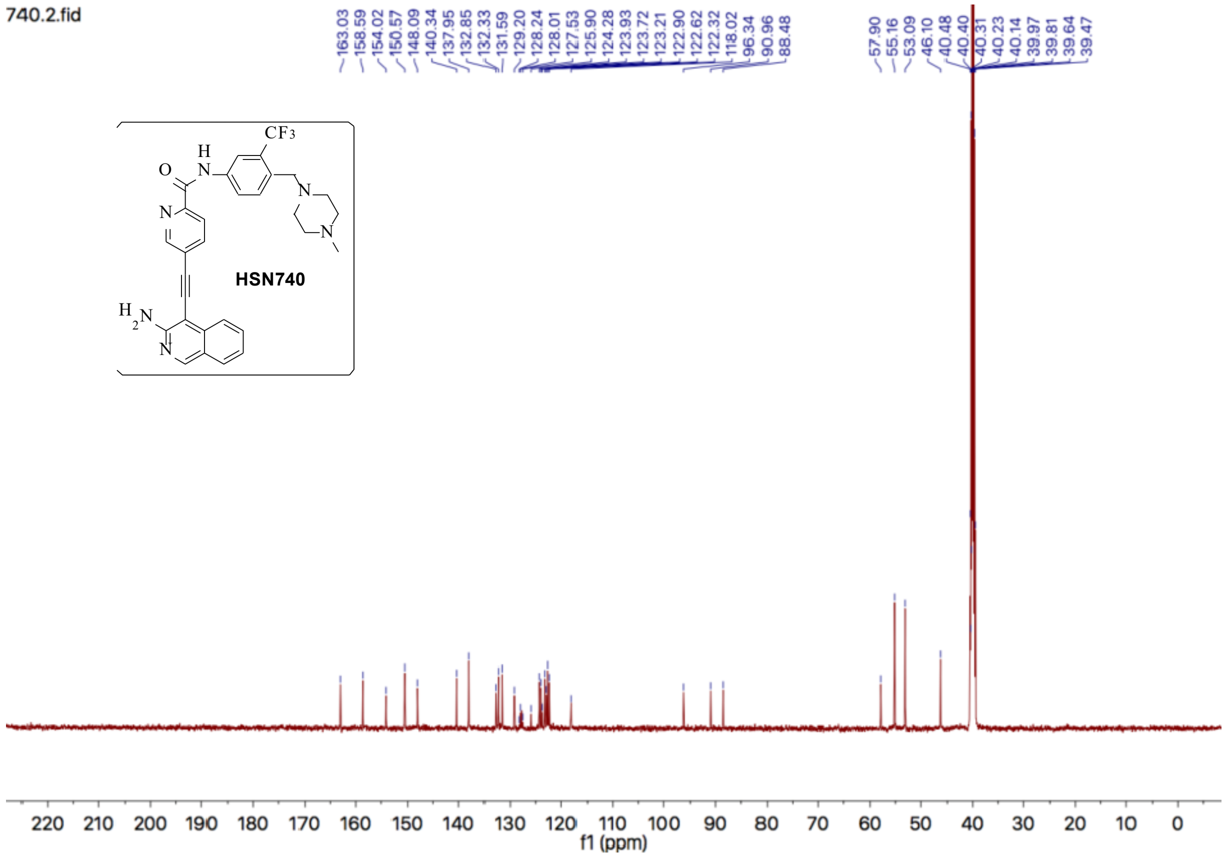
740.2.fid
163.03
158.59
154.02
150.57
148.09
140.34
137.95
132.85
132.33
131.59
129.20
128.24
128.01
127.53
125.90
124.28
123.93
123.72
123.21
122.90
122.62
122.32
118.02
96.34
90.96
88.48
57.90
55.16
53.09
46.10
40.48
40.40
40.31
40.23
40.14
39.97
39.81
39.64
39.47
HSN740
f1 (ppm)

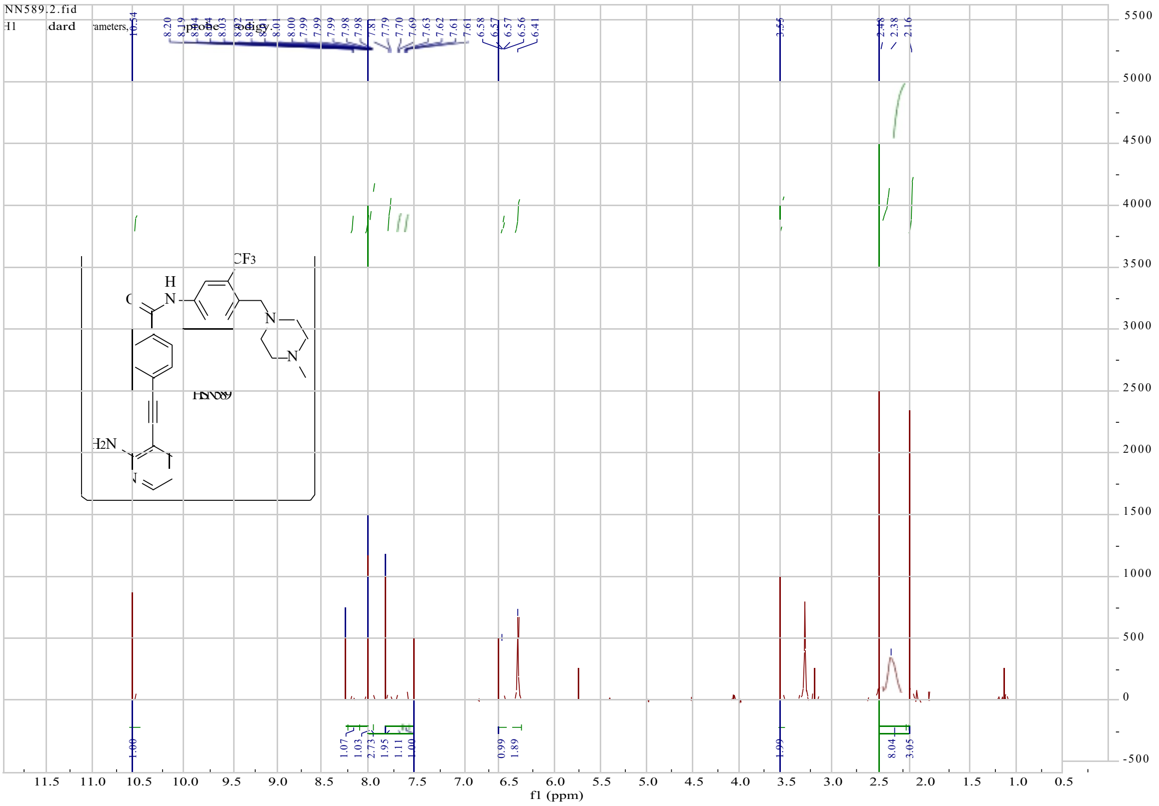
NN589.2.fid
10.54
7.87
7.79
7.70
7.69
7.63
7.62
7.61
7.61
6.58
6.57
6.57
6.56
6.41
3.55
2.48
2.38
2.16
CF3
H
N
N
N
HSN589
H2N
N
1.00
1.07
1.03
2.73
1.95
1.11
1.00
0.99
1.89
1.99
8.04
3.05
f1 (ppm)

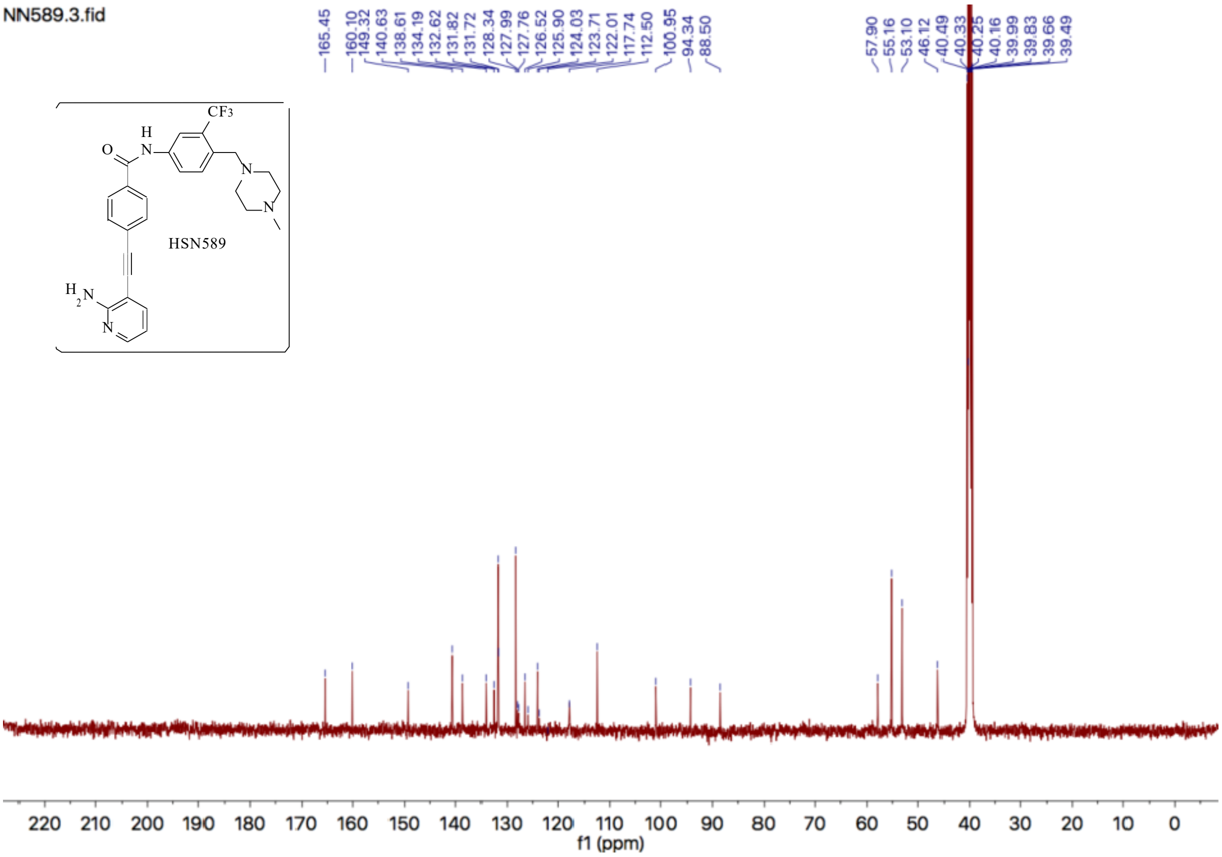
NN589.3.fid
165.45
160.10
149.32
140.63
138.61
134.19
132.62
131.82
131.72
128.34
127.99
127.76
126.52
125.90
124.03
123.71
122.01
117.74
112.50
100.95
94.34
88.50
57.90
55.16
53.10
46.12
40.49
40.33
40.25
40.16
39.99
39.83
39.66
39.49
CF3
H
O
N
N
N
HSN589
H2N
N
f1 (ppm)

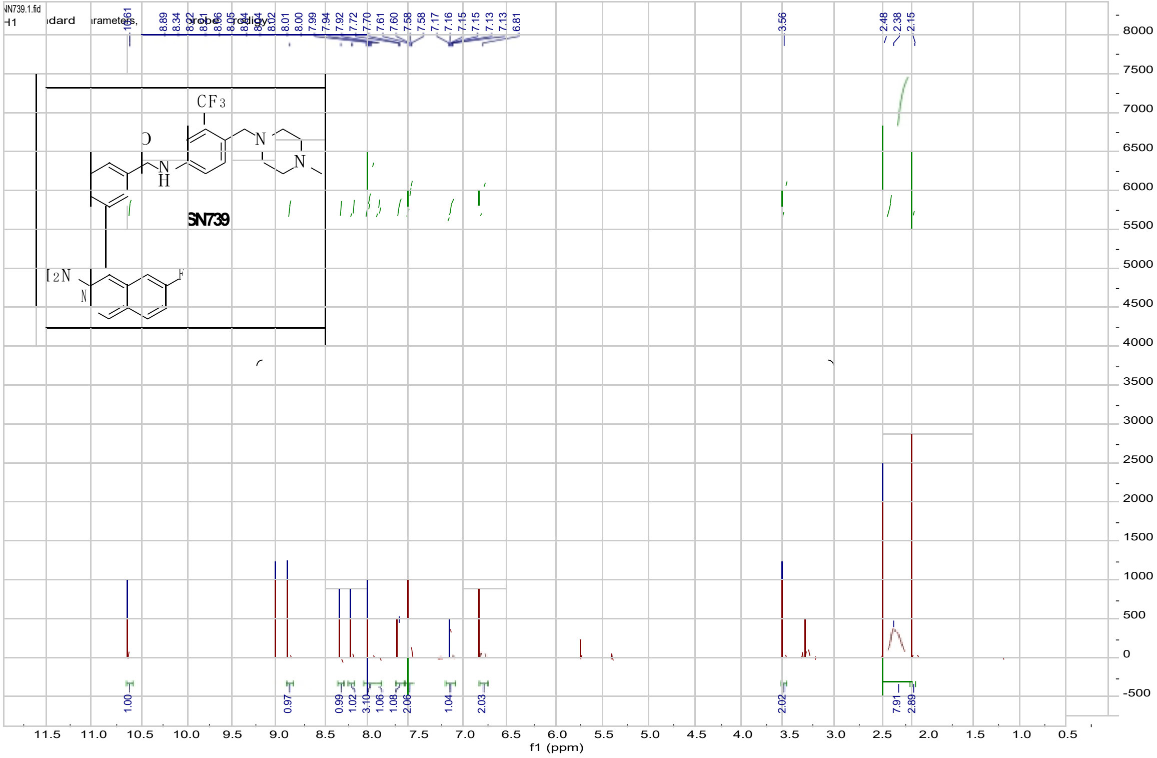

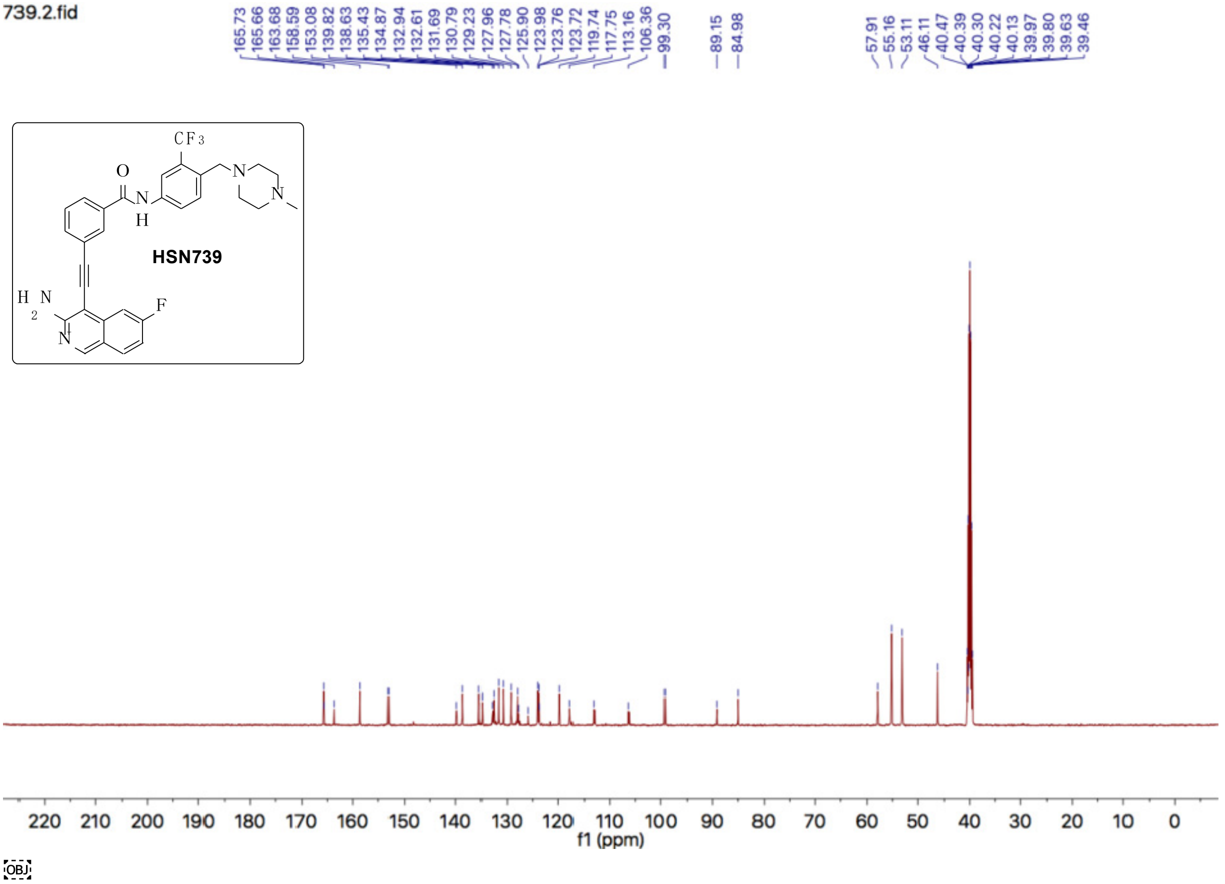

Supplement: Supplementary file 1 [file molecules-27-05085-s001.zip › molecules-1841855-supplementary.pdf]
